# Novel Synthetic Strategies Towards Analogues of Cadaside and Malacidin Antibiotic Peptides

**DOI:** 10.3390/biom15111497

**Published:** 2025-10-23

**Authors:** Katharina Webhofer, Darsha Naidu, Milandip Karak, Stephen A. Cochrane, Christopher J. Morris, Rachael Dickman

**Affiliations:** 1 School of Pharmacy, University College London, 29-39 Brunswick Square, London WC1N 1AX, UKchris.morris@ucl.ac.uk (C.J.M.); 2 Department of Chemistry and Biochemistry, University of California Santa Cruz, Santa Cruz, CA 95064, USA; 3 School of Chemistry and Chemical Engineering, Queen’s University Belfast, Stranmillis Road, Belfast BT9 5AG, UK

**Keywords:** solid-phase peptide synthesis, calcium-dependent antibiotics, structure–activity relationship

## Abstract

With antibiotic resistance becoming an increasingly pressing issue, the search for novel antimicrobial drugs is more important than ever before. The recently discovered calcium-dependent lipopeptides cadaside A/B and malacidin A/B have promising activity against resistant Gram-positive bacteria. With limited reports of synthetic routes towards these peptides available in the literature, especially for cadasides, we herein report a novel on-resin synthesis strategy. We used this strategy to produce fifteen simplified malacidin and cadaside analogues. In addition, both minimum inhibitory concentration and thin layer chromatography assays were conducted to determine antimicrobial activity and advance our understanding of these peptides’ structure–activity relationships.

## 1. Introduction

**Figure 1:**
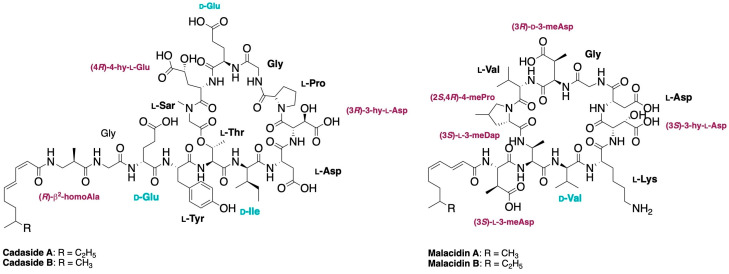
Structures of cadasides A/B (**left**) and malacidins A/B (**right**). Non-canonical amino acids are highlighted in purple and D-amino acids are highlighted in blue. β-substituted residues: (4*R*)-4-hydroxy-L-glutamic acid ((4*R*)-4-hy-L-Glu), (3*R*)-3-hydroxy-L-aspartic acid ((3*R*)-3-hy-L-Asp), (*R*)-β^2^-homo-alanine ((*R*)-β^2^-homoAla), (2*S*,4*R*)-4-methyl-L-proline ((2*S*,4*R*)-4-me-L-Pro), (3R)-D-3-methyl-aspartic acid ((3*R*)-D-3-meAsp), (3*S*)-L-3-methyl-aspartic acid ((3*S*)-L-3-meAsp), (3*S*)-L-3-methyl-diamino propionic acid ((3*S*)-L-3-meDap), and (3*S*)-3-hydroxy-L-aspartic acid ((3*S*)-3-hy-L-Asp).

Each year, more antibiotic resistance-related deaths are reported, with predicted estimates of 10 million annual deaths by 2050 [1]. Finding new drugs to combat this global threat could not only save up to 92 million lives [1] but could also save an estimated $2 to $4.4 trillion in increased healthcare costs and loss of GDP [2]. Calcium-dependent antibiotics (CDAs) have the potential to be part of the solution to this issue, with daptomycin already in clinical use, and further representatives of the class showing promising antimicrobial activities. The first peptide of this class to be identified, isolated in 1983 from *Streptomyces coelicolor*, exerted its antimicrobial effect by forming membrane channels in the presence of Ca^2+^ [3]. This requirement of calcium ions for activity led to the compound being named ‘calcium-dependent antibiotic’ (CDA), a designation later adopted as the eponym for the entire class. It was confirmed through circular dichroism experiments that the activity was dependent on a conformational change induced by binding with calcium [4]. Since then, many representatives of this class have been discovered, most notably daptomycin in 1986, then known as LY 146032 [5]. Not only did daptomycin exhibit potent antimicrobial activity against Gram-positive *Streptococci* and *Enterococci*, but it was also shown to successfully kill oxacillin- and penicillin-resistant *Staphylococcus* strains [6,7], even outperforming vancomycin [8,9]. Most CDAs have a common calcium-binding motif, formed of the amino acids Asp-X-Asp-Gly. In recent years, however, an increasing number of CDAs have been discovered that contain a variation in this motif [10,11,12,13]. This is particularly noteworthy because alanine scanning experiments in daptomycin have shown that altering the calcium-binding motif significantly reduces or completely removes the activity of the drug [14]. With the mechanism of action (MOA) of daptomycin still debated [15,16,17,18], and few crystal structures of CDAs bound to their targets available [19], these varying calcium-binding sequences could be used to further understand the structure–activity relationships (SARs) of this class of compounds. In this work, we focused particularly on two families of CDAs: cadasides and malacidins (Figure 1). Both of these families of peptides are characterised by a lipid tail, a macrocycle comprising nine amino acids, and a non-canonical calcium-binding motif [10,11]. In addition, the cadasides and malacidins contain several non-canonical amino acids, including (4*R*)-4-hy-L-Glu, (3*R*)-3-hy-L-Asp, (*R*)-β^2^-homoAla, (2*S*,4*R*)-4-me-L-Pro, (3*R*)-D-3-meAsp, (3*S*)-L-3-meAsp, (3*S*)-L-3-meDap, (3*S*)-3-hy-L-Asp, several D-amino acids, and the complex lipids (2*E*,4*Z*)-8-methyl-nona-2,4-dienoic acid and (2*E*,4*Z*)-8-methyl-deca-2,4-dienoic acid. Previous research has suggested that the incorporation of non-canonical [20,21] and D-amino acids [22,23,24] can be favourable for peptide stability and efficacy against resistant organisms. These residues therefore represent an intriguing opportunity to understand SARs and improve the properties of other CDAs. The organic synthesis of CDAs remains challenging due to their complexity, not only because of these non-canonical residues, but also their highly anionic nature.

In this work, we established a novel synthetic route towards both cadaside and malacidin analogues. By exploiting the increased efficacy of microwave-assisted reactions, macrocyclisation times and conversions were significantly improved over the reported procedures. Using this route, we synthesised fifteen analogues that were then subjected to minimum inhibitory concentration (MIC) and thin layer chromatography (TLC) assays. MIC profiling showed that simplifying the structure of the peptide macrocycle in the analogues lowered potency by roughly two orders of magnitude, yet activity remained even in the absence of Ca^2+^, implying a change in the mechanism. Complementary TLC assays confirmed this shift: the synthesised malacidin analogues no longer bound lipid II, while results for a subset of the cadaside analogues suggest a possible interaction with the cell wall precursor, bactoprenol (C_55_-P).

## 2. Materials and Methods

### 2.1. General Reagents and Instrumentation

All amino-acid derivatives, unless otherwise stated, were Alloc- or Fmoc-protected, >98% purity (Novabiochem, Merck KGaA, Darmstadt, Germany). (2*R*)-2-methyl*-β*-alanine was purchased from Fluorochem (Fluorochem Limited, Hadfield, UK). Proline preloaded 2-Chlorotrityl chloride (2-CTC) resin (loading: 0.82 mmol/g), *N,N*’-diisopropylcarbodiimide (DIC), 4-dimethylaminopyridine (DMAP), 2-(7-Aza-1*H*-benzotriazole-1-yl)-1,1,3,3-tetramethyluronium hexafluorophosphate (HATU), and *N,N*-diisopropylethylamine (DIPEA) were purchased from Sigma-Aldrich (Merck KGaA, Darmstadt, Germany) or Fluorochem. Lipid II was synthesised according to previously described methods [25]. Undecaprenyl phosphate was synthesised as previously described [26,27]. Anhydrous DMF and dichloromethane were obtained from Sigma-Aldrich. All water used was purified with a Elga Purelab Option-Q deioniser (ELGA LabWater, High Wycombe, UK). DMF used for peptide synthesis was peptide-synthesis grade. The solvents used for HPLC were HPLC-grade and used directly from the bottle. Reactions requiring anhydrous conditions were conducted under a nitrogen atmosphere in oven-dried glassware. Solid-phase peptide synthesis (SPPS) was carried out using a Heidolph Unimax 1010 shaker (Heidolph Scientific Products GmbH, Schwabach, Germany) and either 5 mL or 10 mL Biotage PP-Reactors with PTFE frits (Biotage Sweden AB, Uppsala, Sweden). Samples were processed using a Fisherbrand GT4 Centrifuge (Fisher Scientific, Loughborough, UK) and a Heto PowerDry LL1500 freeze dryer (Thermo Fisher Scientific Inc., Paisley, UK). Microwave reactions were carried out in a Biotage Initiator+ microwave reactor using 0.5–2.0 mL, 2.0–5.0 mL, or 10–20 mL Biotage Microwave Reaction Vials. TLC analysis was on aluminium-backed Sigma-Aldrich TLC plates with visualisation by UV light at 254 nm or by staining with KMnO_4_ or ninhydrin. Flash column chromatography was carried out on a Biotage Selekt with Biotage Sfär Silica Duo 60 μm columns (10–50 g). Peptides were purified by preparative reverse phase HPLC on an Agilent 1260 Infinity II HPLC System with a G7114A VWD detector, a G7111A quaternary pump, and a G1364F fraction collector (Agilent Technologies LDA UK Limited, Stockport, UK). An Agilent Zorbax 300SB-C18 5 μm 250 × 9.4 mm column was used, with detection at 214 and 254 nm. Water (0.1% TFA) and acetonitrile (0.1% TFA) were used as solvents. Chromatograms were analysed using the Agilent CDS OpenLab software, version 2.3.54.

### 2.2. Cadaside Synthetic Procedure

Preloaded resin HN-L-Pro-OCTC (loading = 0.82 mmol/g) was elongated using standard Fmoc SPPS protocols to attach the first 7 amino acid residues. Each reaction step, as outlined in Scheme S1, was followed by a mini cleavage to enable LC-MS analysis prior to progression to the subsequent step. The ester was installed using DIC/DMAP, and the Fmoc group was removed using 2-methylpiperidine. The respective tri-/tetrapeptide (**1**–**10**) was then coupled using standard Fmoc SPPS protocols in an overnight coupling. The Alloc-protecting group was removed, after which the lipid tail was installed. The remaining Fmoc-protecting group was cleaved using 2-methylpiperidine, before cleaving the peptide from the resin. Macrocyclisation was achieved under microwave irradiation, followed by global deprotection in solution. After lyophilisation, the peptides were afforded as a white-to-light yellow solid, which was dissolved in ACN/H_2_O (1/1) and purified by prep-HPLC, on a 5–95% B gradient over 27 min (A = 0.1% TFA in H_2_O, B = 0.1% TFA in ACN).

### 2.3. Malacidin Synthetic Procedure

Preloaded resin HN-L-Pro-OCTC (loading = 0.82 mmol/g) was elongated using standard Fmoc SPPS protocols to attach all amino acid residues and decanoic acid. The Alloc-protecting group was removed, and the peptide was cyclised under microwave irradiation followed by global deprotection in solution. After lyophilisation, the peptides were afforded as a white-to-light yellow solid, which was dissolved in ACN/H_2_O (1/1) and purified by prep-HPLC, on a 5–95% B gradient over 27 min (A = 0.1% TFA in H_2_O, B = 0.1% TFA in ACN).

### 2.4. Analytical Characterisation

Analytical HPLC of peptides was performed on an Agilent 1260 Infinity II system with a Zorbax 300SB-C18 5 µm 250 × 4.6 mm column, with detection at 214 nm using either an Agilent Variable Wavelength Detector or an Agilent Diode Array Detector. A linear solvent gradient of 5–95% MeCN (0.1% TFA) in H_2_O (0.1% TFA) over 55 min was used, at a flow rate of 1 mL/min. LC-MS spectra were recorded on a Shimadzu LC-MS 2020 (Shimadzu UK Limited, Milton Keynes, UK) using a linear gradient 10–95% B over 12 min (A = water 0.1% formic acid; B = acetonitrile, 0.1% formic acid) with a Waters XBridge^®^ MS C18 2.5 μm 3.0 × 50 mm column (Waters Corporation, Milford, MA, USA) at a flow rate of 1 mL/min. UV detection was carried out with a SPD-20A dual wavelength detector at 254 nm and 280 nm. A single quadrupole mass spectrometer was used for mass detection. HRMS spectra were recorded on an Agilent AdvanceBio 6545 XT LC/Q-TOF using a gradient of 3-80-3% B over 12 min (A = water 0.1% formic acid; B = acetonitrile, 0.1% formic acid) with an Agilent PoroShell 120 3.0 × 30 mm column at a flow rate of 0.4 mL/min. Analysis of the chromatograms was conducted using LabSolutions software, version 5.128. Optical rotations were measured at 25 °C, unless otherwise stated, on a Bellingham and Stanley Automatic Digital Polarimeter (Fisher Scientific, Loughborough, UK). Specific rotations are given in 10^−1^ deg cm^2^/g. ^1^H, ^13^C, and all 2D NMR spectra were recorded on a Bruker Avance 400 MHz spectrometer, with chemical shifts (δ) given in ppm relative to the solvent signal and coupling constants (*J*) given in Hz. Carbon signals were assigned from HSQC cross-peaks. Data were processed using TopSpin, version 3.6.3 (Bruker Corporation, Billerica, MA, USA). Where the resonances for symmetric carbon atoms in the Fmoc group could be distinguished, the shift of both is given. Abbreviations used in the ^1^H NMR assignment are as follows: s = singlet, d = doublet, sx = sextet, m = multiplet, and dd = doublet of doublets.

### 2.5. Minimum Inhibitory Concentration (MIC) Assays

MICs were measured in triplicate by microbroth dilution. An overnight culture of *B. subtilis* 168 (DSMZ German Collection of Microorganisms and Cell Cultures GmbH) was inoculated at 5 × 10^5^ CFU/mL in cation-adjusted Mueller–Hinton broth (MHB II) with or without 15 mM or 100 mM CaCl_2_. Serial two-fold peptide dilutions (128 - 1 µg/mL for analogues and 16 - 0.25 µg/mL for daptomycin) were incubated in polypropylene U-bottom 96-well plates (200 µL final volume) at 37 °C for 24 h. MIC was the lowest concentration at which no growth was observed. Optical density measurements at 620 nm were performed using a Thermo Scientific Multiskan FC (Thermo Fisher Scientific Inc., Paisley, UK). MIC was determined from the lowest concentration at which no tubidity was observed, using an untreated positive growth control, and a broth-only sterility control as references.

### 2.6. Thin-Layer Chromatography (TLC) Precursor-Binding Assay

Lipid II and undecaprenyl phosphate (C_55_-P) (1 equiv., 30 µL) in Tris•HCl buffer (0.2% Triton X-100, 26 mM MgCl_2_, and 50 mM CaCl_2_ at pH 7.5) were incubated with the peptides (0.5, 1, 2 equiv.) or nisin (0.1, 0.5, 1 equiv.) in 30 µL water for 1 h. The reaction was then extracted with *^t^*BuOH/6 M PyOAc (2/1 *v/v*, 100 µL) and the organic phase was dried using an Eppendorf vacuum centrifuge 5301 (Fisher Scientific, Loughborough, UK) under reduced pressure. The solid was redissolved in CHCl_3_/MeOH (1/1 *v/v*, 30–60 µL) for spotting onto the TLC plates. In addition to the peptide–lipid mixtures, a control of each peptide/nisin with 30 µL buffer, and a control of each lipid with 30 µL HPLC-grade water were also incubated and subjected to the same workup. Silica TLC plates were run in a CHCl_3_/MeOH/H_2_O/NH_3_ (88/48/10/1 *v/v*) solvent system and stained using iodine vapour, phosphomolybdic acid (PMA), or KMnO_4_ stain, as well as visualisation at 254 nm and 365 nm.

## 3. Results

### 3.1. Establishing the Synthetic Route

#### 3.1.1. Cadaside Synthesis

**Figure 2:**
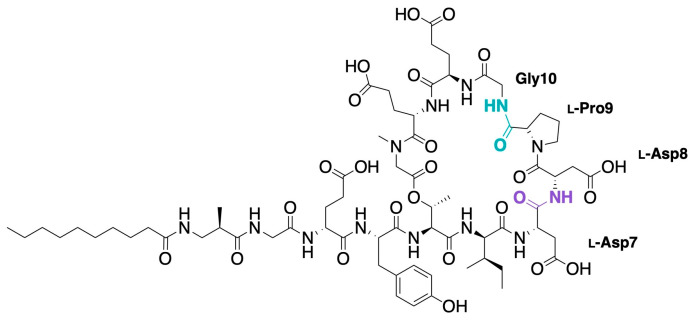
The cyclisation point in a simplified cadaside analogue, described in the literature [28,29] between Asp_7_ and Asp_8_ (purple) and the cyclisation point used in this work’s synthetic approach between Pro_9_ and Gly_10_ (blue).

Given the synthetic complexity of wild-type cadasides, we conducted our method development using simplified analogues. This involved the substitution of non-canonical amino acids for commercially available alternatives, and the substitution of the branched unsaturated lipid tail for decanoic acid. Our planned synthesis of cadaside analogues was developed around the desired cyclisation point. In previously published work on the organic synthesis of cadasides, two aspartic acid residues were chosen as the cyclisation point, possibly contributing to racemisation and the subsequent loss of antimicrobial activity [28,29]. To minimise the possibility of racemisation and other unwanted side reactions and products [30], we chose to head-to-tail-cyclise between proline (*C*-terminus) and glycine (*N*-terminus) (Figure 2). With proline as the *C*-terminal starting residue, we opted to use 2-chlorotrityl chloride resin as its steric bulk helps to prevent aminolysis [31,32].

**Scheme 1:**
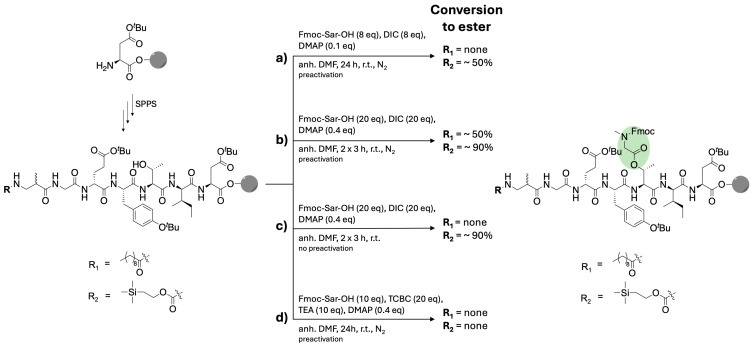
Testing reaction conditions to form the ester between Fmoc-Sar-OH and the unprotected Thr side-chain, yielding the branched peptide (Sar highlighted in green). The starting test peptide contained either a decanoic acid lipid tail (R_1_) or a Teoc-protected beta-amino isobutyric acid (R_2_) at the *N*-terminus. Yields are based on crude conversions as measured by LC-MS (see Supplementary Information Figure S1).

In addition to the cyclisation point, the conditions used for esterification needed to be considered. The incorporation of depsi bonds in lipopeptides has been discussed in previous literature, with opinions varying on the best point to conduct this reaction during the synthesis. While some authors claim better results in the synthesis of daptomycin when the lipid tail is incorporated after esterification [33,34,35], others do not report any influence of the lipid on the esterification conversion [28,29]. To determine the most suitable method for this work, we synthesised a short test peptide and examined four different esterification conditions based on previously reported CDA syntheses (Scheme 1, Figure S1) [28,29,33,36]. Either a decanoyl tail or a 2-(trimethylsilyl)ethoxycarbonyl (Teoc)-protected beta-amino isobutyric acid was included at the *N*-terminus of the test peptide, allowing an examination of whether the presence of the lipid affected conversion to the ester in each case. While most published on-resin esterification procedures report total reaction times of 24 h or longer [28,29,33,35,36], we sought to investigate whether this time could be decreased by repeating the reaction with high molar equivalents of coupling reagent and shorter reaction intervals. We also examined whether preactivation of the amino acid with DIC had any significant influence on conversion. The results of this test showed that incorporation of the lipid tail prior to esterification mostly prevented formation of the depsi bond. Repeated short reactions with high equivalents of amino acid and DIC significantly improved conversion (90%, condition B) compared to one continuous reaction over 24 h with lower molar equivalents (50%, condition A). In the absence of the lipid, no difference was observed between preactivating and not preactivating the sarcosine residue prior to the addition to the resin (conditions B and C). Based on these results, it was decided that the esterification should be conducted prior to the introduction of the lipid tail. The final esterification conditions selected were therefore as follows: Fmoc-Sar-OH (20 equiv.), DIC (20 equiv.), and DMAP (0.4 equiv.) in DMF at room temperature for 2 × 3 h. Additionally, for the next syntheses we opted to orthogonally protect the beta-aminoisobutyric acid residue using an *N*-allyloxycarbonyl (Alloc)-protecting group rather than the Teoc group used in the test peptide. This is because it would allow facile and selective deprotection during subsequent steps of the synthesis route using palladium (0) tetrakis (triphenylphosphine).

**Scheme 2:**
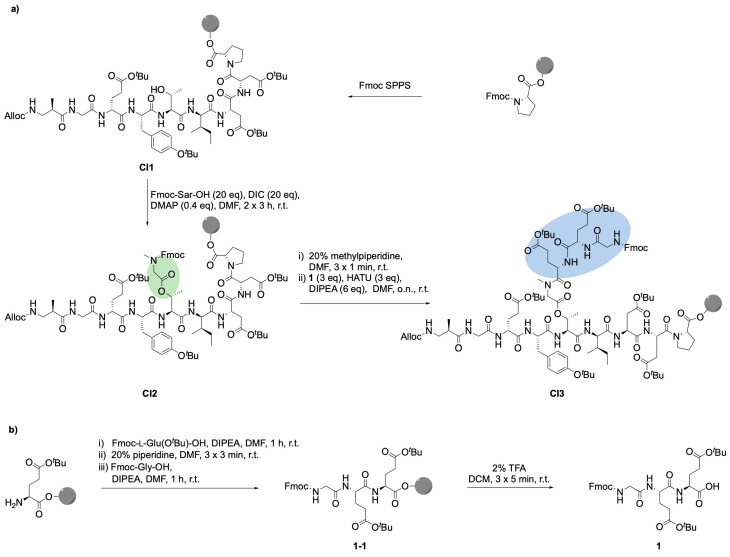
Initial synthetic steps of cadaside analogue synthesis. (**a**) Steps 1–3 of the synthesis towards cadaside analogues. Step 1: Peptide elongation; Fmoc-AA-OH (3 equiv.), HATU (3 equiv.), DIPEA (6 equiv.), DMF (10 mL), 1 h, r.t.; 20% piperidine/DMF (*v/v*, 10 mL), 4 × 3 min, r.t. Step 2: Incorporation of the depsi bond (green) using optimised reaction conditions. Step 3: Incorporation of the remaining amino acids after the branching point as a tripeptide (**1**) (blue). (**b**) SPPS of the tripeptide building block **1** for cadaside analogue synthesis development.

With the esterification conditions established, the synthesis of a full-length cadaside analogue was attempted. The synthesis began with elongation of the peptide on a preloaded HN-Pro-2-CTC resin until the Alloc-beta-aminoisobutyric acid residue, generating linear cadaside intermediate **CI1** (Scheme 2a). The esterification was then conducted using the optimised reaction conditions, producing branched intermediate **CI2**. Following esterification, the remaining three amino acids needed to be installed into the peptide. Unfortunately, incorporation of these amino acids (Glu, D-Glu, and Gly) using standard SPPS conditions frequently resulted in the degradation of the peptide back to its linear form. In a previous attempt to synthesise cadaside B [28], Kovalenko et al. used a dipeptide to elongate the peptide at the branching point. Taking inspiration from this, we tested whether adding the remaining amino acids in our synthesis as a tripeptide would protect the ester from hydrolysis. It was theorised that not only would this minimise the exposure of the depsi bond to base during deprotection steps, but the increased steric bulk could potentially also shield the ester during subsequent reactions. The required tripeptide (**1**) was synthesised using a glutamic acid-preloaded 2-CTC resin according to standard procedures (Scheme 2b). The tripeptide was then cleaved from the resin and used in the coupling reaction with deprotected **CI2**. This generated intermediate **CI3**, with no observable degradation back to linear **CI1** (see Supplementary Information Figure S2).

**Scheme 3:**
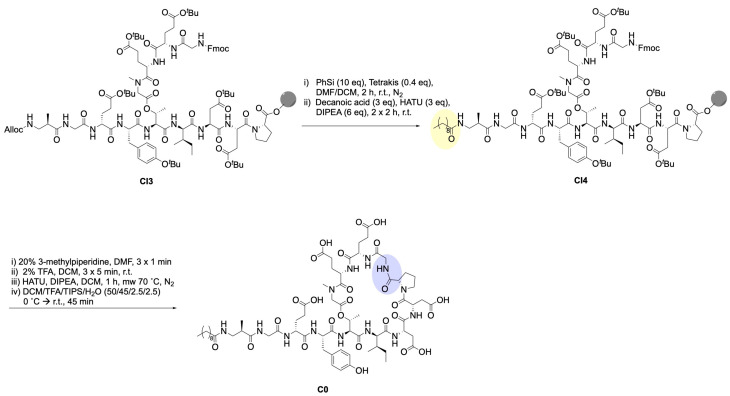
Remaining synthetic steps of cadaside analogue synthesis. Step 1: Deprotecting the Alloc-protected *N*-terminus of β-aminoisobutyric acid and coupling decanoic acid (yellow). Step 2: Fmoc-deprotection, resin cleavage, macrocyclisation between Pro_9_ and Gly_10_ (purple), and global deprotection of the peptide to yield cadaside analogue **C0**.

With all amino acids installed, it was then possible to remove the *N*-terminal Alloc-protecting group and couple the decanoic acid tail to produce lipidated intermediate **CI4** (Scheme 3). The deprotection was achieved using palladium (0) tetrakis(triphenylphosphine) and phenylsilane. After deprotection, the lipid was coupled using standard coupling procedures at 2 × 2 h. This yielded very good conversion as no starting material could be detected upon LC-MS analysis (see Supplementary Information Figure S3) and no noticeable decomposition to the linear peptide could be observed. The *N*-terminus on the peptide branch point was then deprotected using 2-methylpiperidine. This milder base was chosen in order to minimise the chance of degradation of the depsi bond during the reaction, as was reported for a previous synthesis of daptomycin [33].

In previous literature, macrocyclisation has been carried out using various strategies. As the peptide was *C*-terminally linked to the resin in our chosen synthetic strategy, an on-resin cyclisation approach, as used in several daptomycin syntheses [33,34], was not possible. Reported solution-phase cyclisations of depsipeptides used reaction times of 3–48 h [28,29,36]. In this work, we investigated whether a microwave-assisted approach could improve these long reaction times. The peptide was first cleaved from the resin with 2% *v/v* TFA in dichloromethane to produce the protected intermediate ready for cyclisation. We recovered 91% of the theoretical protected peptide yield following the cleavage reaction, verifying that the short cleavage time did not significantly affect the final product yield. To conduct the cyclisation reaction, the peptide was dissolved in dichloromethane to a concentration of 6 mM, basified to pH ~10 with DIPEA, and HATU was added. The reaction was then heated to 70 °C under microwave irradiation for 1 h. Analysis of the crude product by LC-MS revealed no observable degradation to any of the intermediate peptides, and complete conversion of the starting material to the desired cyclic product. The peptide was then globally deprotected to yield simplified cadaside analogue **C0**. A scheme showing the full synthetic procedure is provided in the Supplementary Information (Scheme S1).

During synthesis, the peptide displayed unusual solubility when using standard workup procedures after resin cleavage or global deprotection. When precipitated from ether, especially past the point of esterification, the peptide appeared to form a solid aggregate, likely due to its high hydrophobicity and low pI. All attempts to dissolve these aggregates for analysis using organic solvents, basic or acidic solutions, or chaotropic agents were unsuccessful. To avoid formation of these aggregates, the cleavage procedure was adjusted. After global deprotection, the peptide solution was fully dried under a stream of nitrogen. Once dry, the crude peptide was rinsed with diethyl ether to remove by-products. The peptide could then be dissolved in ACN/H_2_O (1/1) and freeze-dried. This strategy was successfully applied to all malacidin and cadaside analogues in this work.

#### 3.1.2. Malacidin Synthesis

**Scheme 4:**
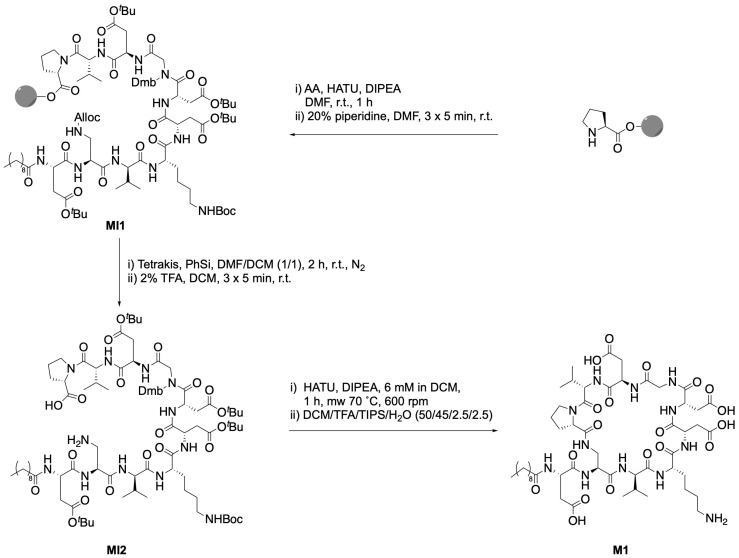
Full synthetic procedure towards malacidin analogues using a microwave-assisted macrocyclisation reaction.

Establishing a synthetic plan for malacidin analogues was significantly facilitated by its simpler structure, as well as two publications on its total organic synthesis [37,38]. As with cadasides, for synthesis development we substituted non-canonical amino acids with commercially available alternatives, and incorporated decanoic acid as the lipid moiety. In contrast to the reported cadaside A/B syntheses, Sun et al. successfully synthesised biologically active malacidins. A range of inactive analogues have also been produced. Our general synthetic approach was modelled after these previous reports. Firstly, we synthesised the linear precursor until the lipid tail from a preloaded proline-2-CTC resin using standard SPPS conditions, generating intermediate **MI1** (Scheme 4). To enable orthogonal deprotection, a side-chain Alloc-protected diamino propionic acid residue was used, later serving as a point for tail-to-side-chain cyclisation. The Alloc-protecting group was removed using palladium (0) tetrakis (triphenylphosphine) and phenylsilane, and the peptide was then cleaved from the resin with 2% *v/v* TFA in dichloromethane. As with the cadaside analogues, macrocyclisation to produce cyclic peptide **MI2** was carried out under microwave irradiation with the aim of decreasing reaction times and increasing reaction efficiency. Following the reaction, no starting material could be observed by LC-MS (see Supplementary Information Figure S4). Following global deprotection and purification by HPLC, simplified malacidin analogue **M1** was afforded in a 1.3% yield.

### 3.2. Synthesis of Analogues

In order to investigate the MOA and SARs of cadasides and malacidins, a range of analogues were synthesised using the novel procedures described above. With the MOA of CDAs generally poorly understood [39], there were several potential sites of interest for modifications. Several studies have shown that moderate modifications to the lipid tail do not negatively affect activity in daptomycin [14,40]. Therefore, a decanoic acid tail was chosen for all analogues. The main focus for our modifications for both peptides was the macrocycle, specifically the regions that resemble the known calcium-binding motif, Asp-X-Asp-Gly. The crystal structure of laspartomycin C bound to calcium demonstrated the importance of the negatively charged side-chains of the Asp residues, as well as the Gly backbone, for calcium binding [19]. This would suggest that the region (4*R*)-hyGlu^12^-D-Glu^11^-Gly^10^ in cadasides and (3*R*)-meAsp^6^-Asp^5^-Gly^4^-(3*R*)-D-hyAsp^3^ in malacidins could be of similar relevance. Another aspect of note was the macrocycle size observed in CDAs with and without the canonical calcium-binding motif. While the macrocycle size of CDAs with the canonical calcium-binding motif is exclusively ten residues [10,11,41,42,43,44], that of cadasides and malacidins comprises only nine residues. Interestingly, this is also the case for other CDAs with non-canonical binding motifs, such as dilarmycins [12] and ambocidins [13] (see Supplementary Information Table S1). With this in mind, we designed malacidin and cadaside analogues that contained ten residues within the macrocycle. With the importance of Gly residues demonstrated through the laspartomycin C crystal structure, Gly was chosen as the additional amino acid. The specific position of insertion was chosen in order to yield structures as similar to the canonical calcium-binding motif as possible.

To assess the influence of stereochemistry on biological activity, we also designed analogues with either the D- or L-configurations in key acidic residues. The reason for this is that in the native cadasides, stereochemistry appears to be critical for activity. For example, the only published organic synthesis of wild-type cadaside B failed to produce a bioactive cadaside, with epimerisation and stereochemical misassignment cited as likely reasons for the loss of function [28]. This underscores the importance of maintaining precise chiral configurations in the molecule. However, the role of stereochemistry in simplified analogues remains unclear. Because these analogues lack many of the structural features of the native molecule, including *β*-substituted residues and more complex lipids, it is uncertain whether chirality at a single residue (such as Glu11) would have the same level of influence on activity. By including both D- and L-variants in our analogue series, we aimed to probe how sensitive the simplified scaffold is to stereochemical changes, and whether this parameter is as tightly linked to function outside the full native context. As native cadasides are enriched in Glu/hyGlu rather than Asp, we also varied the identity of the acidic residues to investigate whether side-chain length influences activity in this minimal context. Specifically, we created cadaside analogues with Glu-Asp substitutions to test whether a Glu-rich sequence remains functionally relevant when other native features are removed.

**Figure 3:**
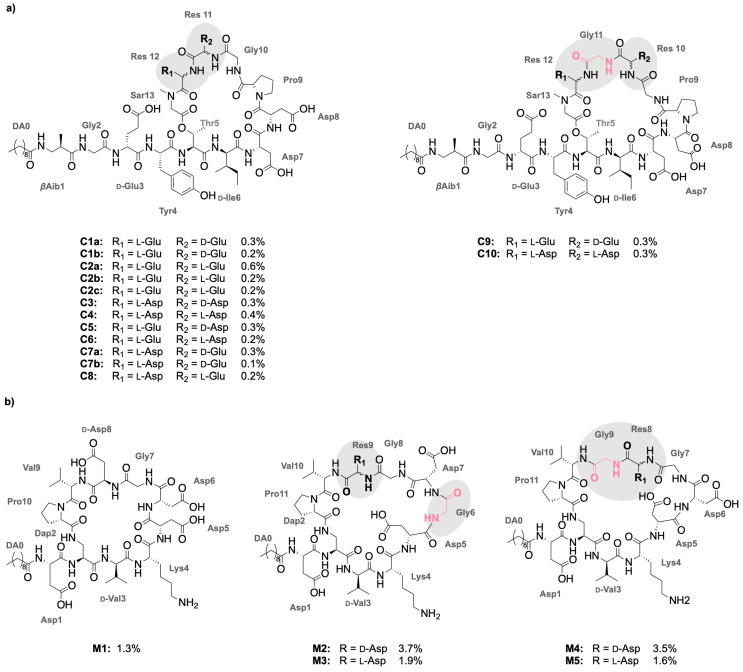
Structures of cadaside and malacidin analogues prepared in this work. Percentage yields for isolated peptides are indicated next to each compound. (**a**) Cadaside analogues with nine-membered macrocycle (**left**) and with ring expansion (pink) with glycine (**right**). Positions which vary between analogues are indicated in grey. a/b/c indicate multiple isolates with the same monoisotopic mass from the synthesis. (**b**) Malacidin analogues with nine-membered macrocycle (**left**) and with ring expansion (pink) with glycine at position six (**middle**) and position nine (**right**). Positions which vary between analogues are indicated in grey.

Ten cadaside analogues and five malacidin analogues were designed and synthesised with these strategies in mind. For cadaside analogue syntheses, the required tripeptides (for 9-membered macrocycles) and tetrapeptides (for 10-membered macrocycles) were first prepared (see Supplementary Information Scheme S2). All analogue peptides were then synthesised according to the novel procedures outlined above (Figure 3). Cadaside ring-expanded analogue **C10** contains the canonical calcium-binding motif (Asp^13^-Gly^12^-Asp^11^-Gly^10^). For the ring expansion in malacidins, two positions, three and six, were deemed suitable for the insertion of an additional glycine residue. This yielded two different analogue topologies, both bearing the canonical calcium-binding motif (**M2** and **M3** Asp^9^-Gly^8^-Asp^7^-Gly^6^, **M4** and **M5** and Asp^6^-Gly^7^-Asp^8^-Gly^9^). For several of the cadaside analogues, more than one peak containing the target mass was isolated during HPLC purification, suggesting the formation of isomers during the synthesis and impacting the isolated yields. Although the exact nature of the isomers was not determined, all peaks containing the target mass were isolated and purified for biological testing. Acceptable yields could be achieved for all malacidin analogues, with no additional isomers observed during purification and analysis. Reported yields for previously published syntheses of wild-type and analogue cadasides and malacidins ranged between 0.4–16% [37] and 5–17% [28,29], respectively, for comparable strategies. Though the yields obtained in this work were lower, 0.1–0.6% and 1.3–3.7%, respectively, the overall reaction times are significantly shorter than for previous syntheses, and sufficient peptide was isolated to enable an initial assessment of bioactivity (Figure 3).

### 3.3. Assessment of Activity via MIC and TLC Assays

With the purified analogues in hand, we moved to evaluating the activity of each compound through both MIC assays against *B. subtilis* 168 as a model Gram-positive bacterium, and TLC assays (Table 1). While MIC testing provides a measure of antimicrobial activity, TLC assays allow investigation of whether the peptides bind to specific lipids. Where possible, the peptides were tested both in the presence and absence of calcium ions to observe any changes in calcium dependence. Daptomycin was used as a positive control as wild-type malacidins and cadasides were unavailable. We found that all of our analogues exhibited activity at 128 µg/mL, with some also showing decreased turbidity at 64 µg/mL (**C2a**, **C5**, **C7b**, **C8**, and elongated **C9**). In addition, while the daptomycin positive control showed a 16- to 32-fold decrease in activity in the absence of calcium, no change in activity was observed for any of our analogue peptides in the absence of calcium. Calcium-dependence was therefore eliminated by the structural changes introduced in the analogues, albeit at the expense of potency. Furthermore, this suggested that the MOA is different for the analogue peptides compared to their wild-type counterparts, which we sought to clarify through TLC binding assays.

While the molecular target of malacidins was identified as lipid II in previous studies [10], no target has been identified for cadasides. Based on the known mechanisms for other CDAs (see Supplementary Information Table S2), we theorised that the cadasides are likely to target an undecaprenol-anchored cell wall precursor. Given the known affinity of laspartomycin C to C_55_-P [45], we opted to examine the binding of our analogues to both C_55_-P and lipid II. The lantibiotic nisin, a known lipid II binder, was used as a positive control for lipid II binding. Positive binding of nisin to lipid II was indicated by the decreased intensity of lipid II observed on the TLC plate. None of our analogue peptides showed affinity towards lipid II. In previous studies using TLC assays, complex formation between C_55_-P and laspartomycin C was characterised by the emergence of an additional spot on the plate, corresponding to a peptide–lipid–calcium complex. We did not observe this for any of our analogue peptides. However, a similar behaviour to that of lipid II in the presence of nisin could be seen for some cadaside analogues, with the corresponding C_55_-P spot no longer visible on the plate with peptides **C1b**, **C2a**, **C2b**, **C2c**, **C3**, and **C6**. While this is not a definitive indicator for binding, it could indicate a potential interaction between these analogues and C_55_-P.

## 4. Discussion

The goal of this study was to establish a rapid, reproducible route to malacidin- and cadaside-type macrocyclic lipopeptides, while simultaneously probing how structural simplification reshapes their antimicrobial profile. By implementing our new synthetic design, we reduced total bench time for synthesis of ten cadaside and five malacidin analogues to <1 week per peptide, despite the use of manual SPPS. The critical advance was an on-resin esterification protocol employed in cadaside syntheses: DIC/DMAP-mediated esterification reached near-quantitative conversion in 2 × 3 h, equalling a >75% reduction versus published on-resin methods using these reagents [33], and removing the need for pre-activation. Initial experiments showed that the presence of a decanoyl lipid tail severely retards ester formation. This is a valuable insight for future studies of lipo-depsipeptide SARs. Protecting the fragile ester during chain extension also required a shift from sequential SPPS to block coupling. Installing the final residues as tri- or tetrapeptides eliminated cleavage to the linear precursor and further shortened overall synthesis duration. Microwave-assisted macrocyclisation (HATU/DIPEA, 70 °C, 1 h) proved broadly applicable for both families of peptides, including for depsipeptides, and delivered clean conversions.

Biological testing of the synthesised peptides revealed that replacing *β*-modified residues with proteinogenic analogues diminished peptide activity against *B. subtilis* 168 by ~100-fold (MIC 128 µg/mL) compared to reported activities for the wild-type peptides [10,11], and abolished Ca^2+^ dependence. This finding of a decrease in activity compared to the wild-type is consistent with all previous reports of malacidin and cadaside analogues, for which no antimicrobial activity was observed within the tested concentration ranges [28,29,37]. Together with the results reported here, this underscores the importance of the presence and configuration of the β-modified residues for antimicrobial activity in malacidins and cadasides. Thin-layer chromatography assays with cell wall precursors verified that none of the simplified malacidins bound to lipid II, though results for several cadaside analogues appeared to indicate a potential interaction with C_55_-P. This suggests that the *β*-modified residues tune both potency and calcium binding. Loss of precursor binding is not entirely unexpected: in other calcium-dependent antibiotics, Ca^2+^ coordination is required for lipid engagement [19,45], whereas our analogues retain activity without Ca^2+^. Selective Asp/Glu rearrangements and macrocycle enlargement may partially restore bactericidal efficiency, as indicated by a decrease in turbidity at 64 µg/mL for select analogues. This observation suggests that stereochemical fidelity is central to potency and that charge patterning and ring size could influence calcium dependence and target engagement. Further testing will be required to substantiate these hypotheses. Together with their amphipathic architecture, we hypothesise a more generic membrane-active mechanism, such as surface disruption or pore formation, rather than high-affinity precursor recognition. Limitations however remain. The production of by-products reduced cadaside isolated yields in this work, possibly the result of proline *cis*/*trans* isomerism or racemisation. Additionally, active wild-type cadasides are still yet to be accessed via total synthesis. Finally, while the new analogues reported here retained measurable antimicrobial activity, further optimisation is needed to regain the potency of the wild-type peptides. If analogues with improved antimicrobial potency can be identified, it will be important to conduct toxicology testing to provide a complete assessment of therapeutic relevance, especially given the potential for analogues to operate via an altered mechanism of action.

## 5. Conclusions

In summary, we report a concise total synthesis of cadaside analogues and a streamlined route towards malacidin-type macrocyclic peptides. Key innovations, namely a fast on-resin esterification and microwave-accelerated macrocyclisation, reduced total synthesis time to ≤7 days per peptide. This method is expected to be readily transferrable to other acidic macrocyclic peptides. While the analogues produced here are not promising lead compounds in themselves due to their high MIC, our work provided the methodology necessary to efficiently synthesise a library of 5 malacidin and 10 cadaside analogues. Testing the resulting analogue library in MIC and TLC assays established that the *β*-modified residues in wild-type malacidins and cadasides are responsible for their potency and Ca^2+^ dependence. Our synthesised analogues operate via a calcium-independent MOA, and results of the TLC assays suggested that some of the cadaside analogues possibly interact with the cell wall precursor C_55_-P. Further verification via measurement of binding affinity between C_55_-P and the peptides is required to provide conclusive evidence of this interaction. Although the potency of our analogues is lower than that of wild-type peptides, our work provides the methodology necessary for future SAR exploration and lays the groundwork for the rational design of next-generation agents against Gram-positive pathogens.

## Figures and Tables

**Table 1:** Results of MIC assay against *B. subtilis* 168 and TLC assay with C_55_-P and lipid II. MIC assays were performed in polypropylene U-bottom plates in cation-adjusted Mueller–Hinton broth II (MHB II), with 0, 15, or 100 mM CaCl_2_. Daptomycin was the positive control. MIC values are the lowest concentration (two-fold dilution series, triplicate) showing no visible growth. TLC assays were performed on silica TLC plates using a solvent system of CH_3_Cl/MeOH/H_2_O/NH_3_ (88/48/10/1 *v/v*). Lipid II was the positive control for lipid II binding. Daptomycin was the negative control for C_55_-P binding. “+” indicates binding confirmed, “-” indicates no binding, “~” indicates potential binding with absence of precursor band, "n.a." indicates not applicable, and “n.d.” indicates MIC or binding was not determined. Examples of assay results are given in the Supplementary Information Figures S5–S7.

Peptide	Ring Size	Ca^2+^ Concentration [mM]	MIC + Ca^2+^ [µg/mL]	MIC − Ca^2+^ [µg/mL]	Lipid II	C_55_-P
Daptomycin	10	15	1	16	-	-
Daptomycin	10	100	0.5	16	-	-
Nisin	n.a.	n.a.	n.d.	n.d.	+	n.d.
M1	9	15	128	128	-	-
M2	10	15	128	128	-	-
M3	10	15	128	128	-	-
M4	10	15	128	128	-	-
M5	10	15	128	128	-	-
C1a	9	100	128	128	-	-
C1b	9	100	128	n.d.	-	~
C2a	9	100	128	128	-	~
C2b	9	100	128	n.d.	-	~
C2c	9	100	128	n.d.	-	~
C3	9	100	128	128	-	~
C4	9	100	128	128	-	-
C5	9	100	128	128	-	-
C6	9	100	128	128	-	~
C7a	9	100	128	128	-	-
C7b	9	100	128	128	-	-
C8	9	100	128	128	-	-
C9	10	100	128	128	-	-
C10	10	-	n.d.	n.d.	n.d.	n.d.

## Data Availability

The data supporting this article have been included in the article/Supplementary Information. Further inquiries can be directed to the corresponding author.

## Supplementary Materials

The following Supplementary Information can be downloaded at: https://www.mdpi.com/article/10.3390/biom15111497/s1, Supplementary Figures, Schemes, and Tables: Figures S1–S7, Schemes S1 and S2, Table S1 and Table S2; supplementary methods and characterisation data; supplementary references [10,11,12,13,17,41,44,45,46,47,48,49,50,51,52,53].
