# Supplementary material for: Novel Synthetic Strategies Towards Analogues of Cadaside and Malacidin Antibiotic Peptides
Source: Biomolecules. 2025 Oct 23;15(11):1497. doi: 10.3390/biom15111497 (PMC12650660; doi:10.3390/biom15111497)
Supplement: Supplementary file 1 [file biomolecules-15-01497-s001.zip › Biomolecules_3919500_SI.pdf]

SUPPLEMENTARY INFORMATION

Novel Synthetic Strategies Towards Analogues of Cadaside and Malacidin Antibiotic Peptides

Katharina Webhofer,[1] Darsha Naidu,[1,2] Milandip Karak,[3] Stephen A. Cochrane,[3] Christopher J. Morris,[1] Rachael Dickman[1*]

[1] School of Pharmacy, University College London, 29-39 Brunswick Square, London, WC1N 1AX, UK
[2] Department of Chemistry and Biochemistry, University of California Santa Cruz, Santa Cruz, CA 95064, USA
[3] School of Chemistry and Chemical Engineering, Queen's University Belfast, Stranmillis Road, Belfast, Northern Ireland, BT9 5AG, UK
[*] Correspondence: rachael.dickman.13@ucl.ac.uk

Table of Contents

1. SUPPLEMENTARY FIGURES, SCHEMES AND TABLES 4

2. GENERAL PROCEDURES FOR PEPTIDE SYNTHESIS 15

GENERAL PROCEDURE 1 15
GENERAL PROCEDURE 2 15
GENERAL PROCEDURE 3 15
GENERAL PROCEDURE 4 15
GENERAL PROCEDURE 5 15
GENERAL PROCEDURE 6 15
GENERAL PROCEDURE 7 15
GENERAL PROCEDURE 8 16
GENERAL PROCEDURE 9 16
GENERAL PROCEDURE 10 16
MASS CALCULATION VIA TYROSINE ABSORPTION AT 280 NM 17

3. SYNTHESIS OF BUILDING BLOCKS 19

SPPS OF FMOC-GLY-L-GLU(O^{T}BU)-L-GLU(O^{T}BU)-OH (1) 19
SPPS OF FMOC-GLY-D-GLU(O^{T}BU)-L-GLU(O^{T}BU)-OH (2) 20
SPPS OF FMOC-GLY-D-ASP(O^{T}BU)-L-ASP(O^{T}BU)-OH (3) 21
SPPS OF FMOC-GLY-L-ASP(O^{T}BU)-L-ASP(O^{T}BU)-OH (4) 22
SPPS OF FMOC-GLY-D-ASP(O^{T}BU)-L-GLU(O^{T}BU)-OH (5) 23
SPPS OF FMOC-GLY-L-ASP(O^{T}BU)-L-GLU(O^{T}BU)-OH (6) 24
SPPS OF FMOC-GLY-D-GLU(O^{T}BU)-L-ASP(O^{T}BU)-OH (7) 25
SPPS OF FMOC-GLY-L-GLU(O^{T}BU)-L-ASP(O^{T}BU)-OH (8) 26
SPPS OF FMOC-GLY-D-GLU(O^{T}BU)-GLY-L-GLU(O^{T}BU)-OH (9) 27
SPPS OF FMOC-GLY-L-ASP(O^{T}BU)-(DMB)GLY-L-ASP(O^{T}BU)-OH (10) 28
(3*S*)-3-(ALLYLOXYCARBONYL)AMINO-2-METHYLPROPANOIC ACID (11) (ALLOC-L-BAIBA-OH) 29
3-[(2-(TRIMETHYLSILYL)ETHOXY)CARBONYL]AMINO-2-METHYLPROPANOIC ACID (12) (TEOC-L-BAIBA-OH) 30

4. TOTAL SYNTHESIS OF CADASIDE ANALOGUES 31

CADASIDE TEST ANALOGUE C0 32
PREPARATION OF BRANCHED PRECURSOR CI2 FOR CADASIDE ANALOGUES C1-C10 **33**
SYNTHESIS OF ANALOGUES **35**
SPPS OF C1 (SIMPLIFIED CADASIDE) 35
SPPS OF C2 (SIMPLIFIED CADASIDE, D-GLU11 → L-GLU11) 36
SPPS OF C3 (SIMPLIFIED CADASIDE, D-GLU11 → D-ASP11, L-GLU12 → L-ASP12) 37
SPPS OF C4 (SIMPLIFIED CADASIDE, D-GLU11 → L-ASP11, L-GLU12 → L-ASP12) 38
SPPS OF C5 (SIMPLIFIED CADASIDE, D-GLU11 →D-ASP11) 39
SPPS OF C6 (SIMPLIFIED CADASIDE, D-GLU11 → L-ASP11) 40
SPPS OF C7 (SIMPLIFIED CADASIDE, L-GLU12 → L-ASP12) 41
SPPS OF C8 (SIMPLIFIED CADASIDE, D-GLU11 → L-GLU11, L-GLU12 → L-ASP12) 42
SPPS OF C9 (SIMPLIFIED CADASIDE, GLY INSERTION POS12) 43

SPPS OF C10 (SIMPLIFIED CADASIDE, GLY INSERTION POS12, D-GLU11 → L-ASP11, L-GLU13 → L-ASP13) 44

5. TOTAL SYNTHESIS OF MALACIDIN ANALOGUES M1-M5 **45**

M1 (SIMPLIFIED MALACIDIN) 46
M2 (SIMPLIFIED MALACIDIN, GLY6 INSERTION) 47
M3 (SIMPLIFIED MALACIDIN, GLY6 INSERTION, D-ASP9 → L-ASP9) 48
M4 (SIMPLIFIED MALACIDIN, GLY9 INSERTION) 49
M5 (SIMPLIFIED MALACIDIN, GLY9 INSERTION, D-ASP8 → L-ASP8) 50

6. ANALYTICAL DATA **51**

7. REFERENCES **81**

1. Supplementary Figures, Schemes and Tables

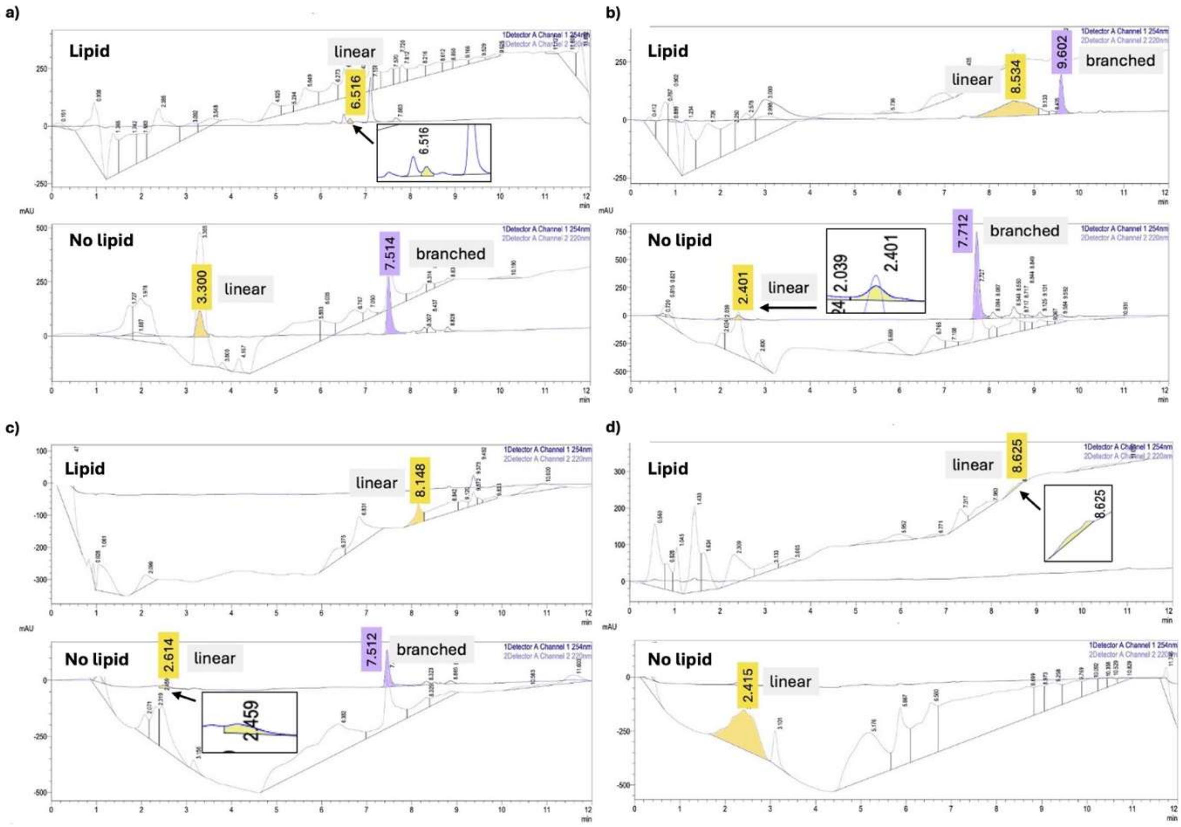

Figure S1: LC-MS UV chromatograms showing the conversion after the reaction of linear, lipidated peptides (top) and linear, non-lipidated peptides (bottom) to branched peptides; zoomed in sections for small peaks inset. Other peaks visible in the chromatogram correspond to impurities as crude cleaved peptides were analysed. **a)** Conversion using method **a** (including preactivation of amino acid) with Fmoc-Sar-OH (8 equiv.), DIC (8 equiv.), DMAP (0.1 equiv.) over 24 h, r.t., N_2. **b)** Conversion using method **b** (including preactivation of amino acid) with Fmoc-Sar-OH (20 equiv.), DIC (20 equiv.), DMAP (0.4 equiv.) over 2 x 3 h, r.t., N_2. **c)** Conversion using method **c** (without preactivation of amino acid) with Fmoc-Sar-OH (20 equiv.), DIC (20 equiv.), DMAP (0.4 equiv.) over 2 x 3 h, r.t.. **d)** Conversion using method **d** (including preactivation of amino acid) with Fmoc-Sar-OH (10 equiv.), TCBC (10 equiv.), TEA (10 equiv.), DMAP (0.4 equiv.) over 24 h, r.t., N_2.

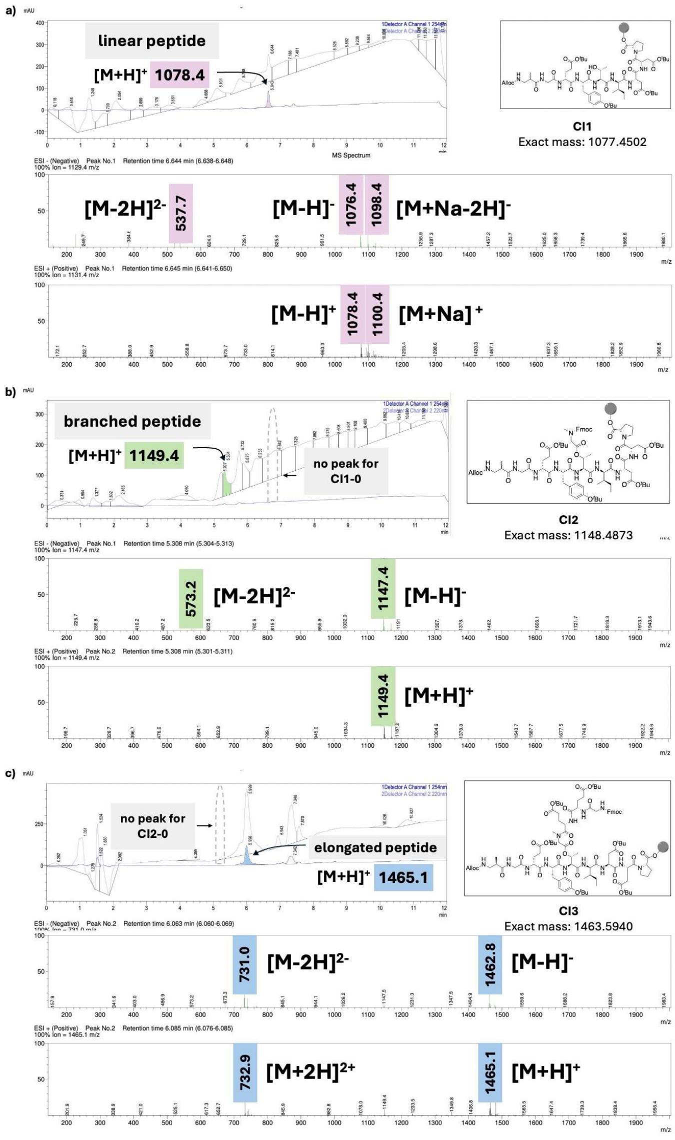

Figure S2: Initial steps of cadaside synthetic route development, from H-Pro-2-CTC resin to **CI3** (highlighted in blue). Progression through the synthesis as observed by LC-MS of crude isolated intermediates. No degradation back to linear **CI1** (highlighted in pink) or **CI2** (highlighted in green) was observed when coupling the final 3 residues as a tripeptide. Other peaks visible in the chromatogram correspond to impurities as crude cleaved peptides were analysed. **a)** LC-MS chromatogram and spectra (ESI+, ESI-) of **CI1** linear intermediate (Alloc-protected, resin cleaved, side-chain deprotected) at an exact mass of 1077.4502 for $C_{47}H_{67}N_9O_{20}$; Expected: $[M+H]^+$ 1078.5 *m/z,* $[M+Na]^+$ 1100.4 *m/z,* $[M-H]^-$ 1076.4 *m/z,* $[M+Na-2H]^-$ 1098.4 *m/z,* $[M-2H]^{2-}$ 537.7 *m/z;* Found: $[M+H]^+$ 1078.4 *m/z,* $[M+Na]^+$ 1100.4 *m/z,* $[M-H]^-$ 1076.4 *m/z,* $[M+Na-2H]^-$ 1098.4 *m/z,* $[M-2H]^{2-}$ 537.7 *m/z*. **b)** LC-MS chromatogram and spectra (ESI+, ESI-) of **CI2** branched intermediate (Alloc-protected, Fmoc-deprotected, side-chain deprotected, resin cleaved) at an exact mass of 1148.4873 for $C_{50}H_{72}N_{10}O_{21}$; Expected: $[M+H]^+$ 1149.5 *m/z*, $[M-H]^-$ 1147.5 *m/z*, $[M-2H]^{2-}$ 573.2 *m/z*; Found: $[M+H]^+$ 1149.4 *m/z*, $[M-H]^-$ 1147.4 *m/z*, $[M-2H]^{2-}$ 573.2 *m/z*. **c)** LC-MS chromatogram and spectra (ESI+, ESI-) of **CI3** branched intermediate (Alloc-protected, Fmoc-deprotected, side-chain deprotected, resin cleaved) at an exact mass of 1463.5940 for $C_{62}H_{89}N_{13}O_{28}$; Expected: $[M+H]^+$ 1464.6 *m/z,* $[M+2H]^{2+}$ 732.8 *m/z,* $[M-H]^-$ 1462.6 *m/z,* $[M-2H]^{2-}$ 730.8 *m/z;* Found: $[M+H]^+$ 1465.1 *m/z,* $[M+2H]^{2+}$ 732.9 *m/z,* $[M-H]^-$ 1462.8 *m/z,* $[M-2H]^{2-}$ 731.0 *m/z*.

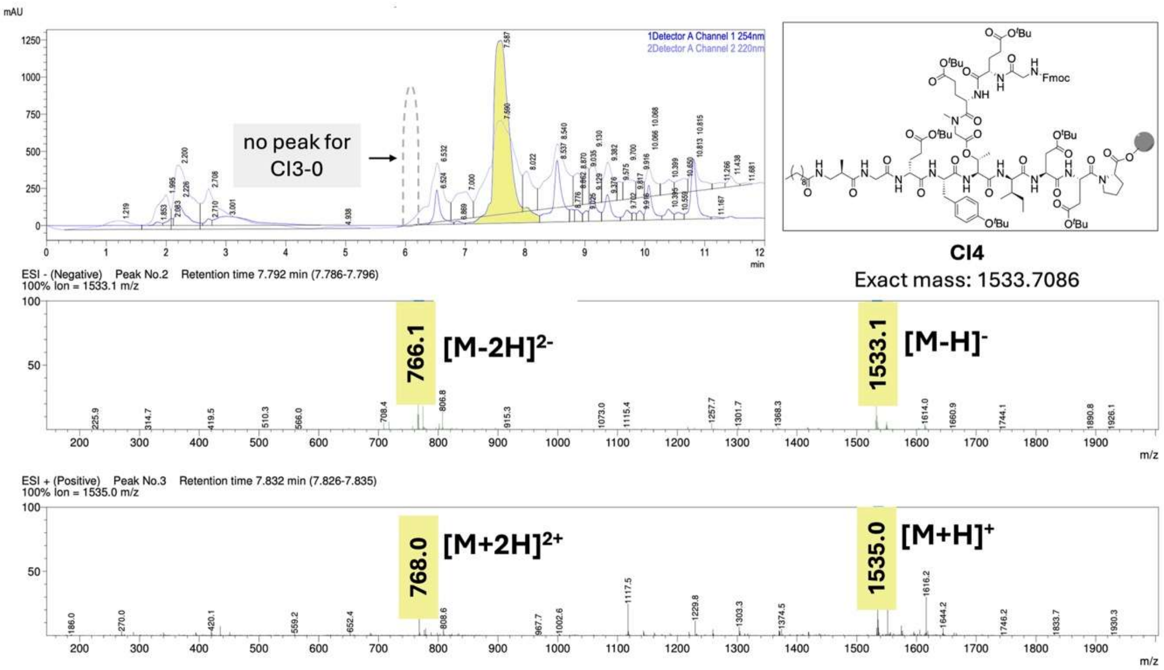

Figure S3: Progression through cadaside synthetic route development, from resin-bound **Cl3** to **Cl4**, as observed by LC-MS. No unreacted **Cl3** was observed. Other peaks visible in the chromatogram correspond to side products from the synthesis and were not determined in detail. LC-MS and spectra (ESI+, ESI-) of lipidated **Cl4** (highlighted in yellow), non-cyclised intermediate (Fmoc-deprotected) at an exact mass of 1533.7086 for $C_{68}H_{103}N_{13}O_{27}$; Expected: $[M+H]^+$ 1534.7 *m/z*, $[M+2H]^{2+}$ 767.9 *m/z*, $[M-H]^-$ 1532.7 *m/z*, $[M-2H]^{2-}$ 765.8 *m/z*; Found: $[M+H]^+$ 1535.0 *m/z*, $[M+2H]^{2+}$ 768.0 *m/z*, $[M-H]^-$ 1533.1 *m/z*, $[M-2H]^{2-}$ 766.1 *m/z*.

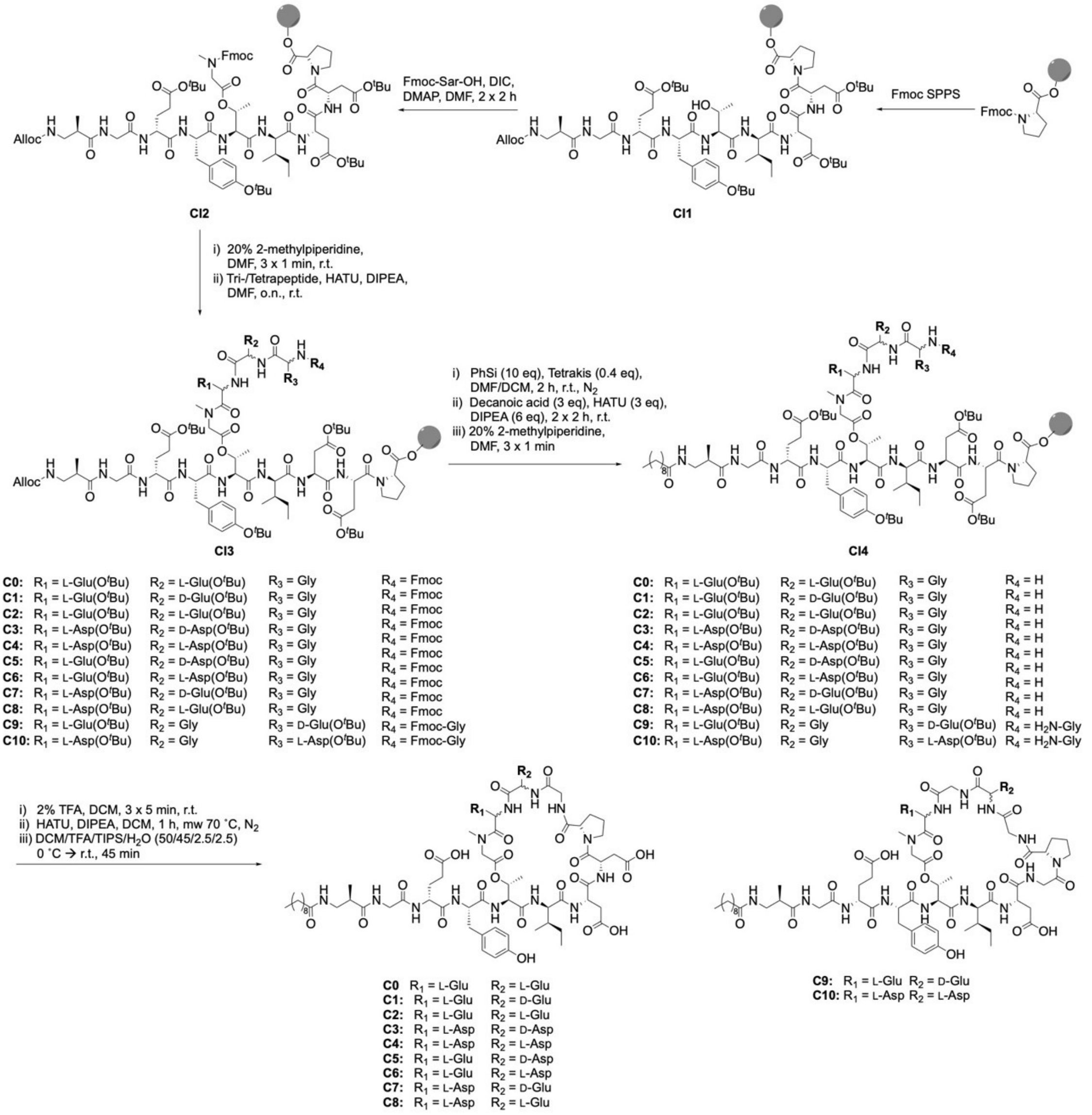

Scheme S1: Total synthesis of cadaside analogues in this work. **C0**-**C8** contain 9-membered macrocycles, **C9** and **C10** are ring-expanded analogues with 10-membered macrocycles.

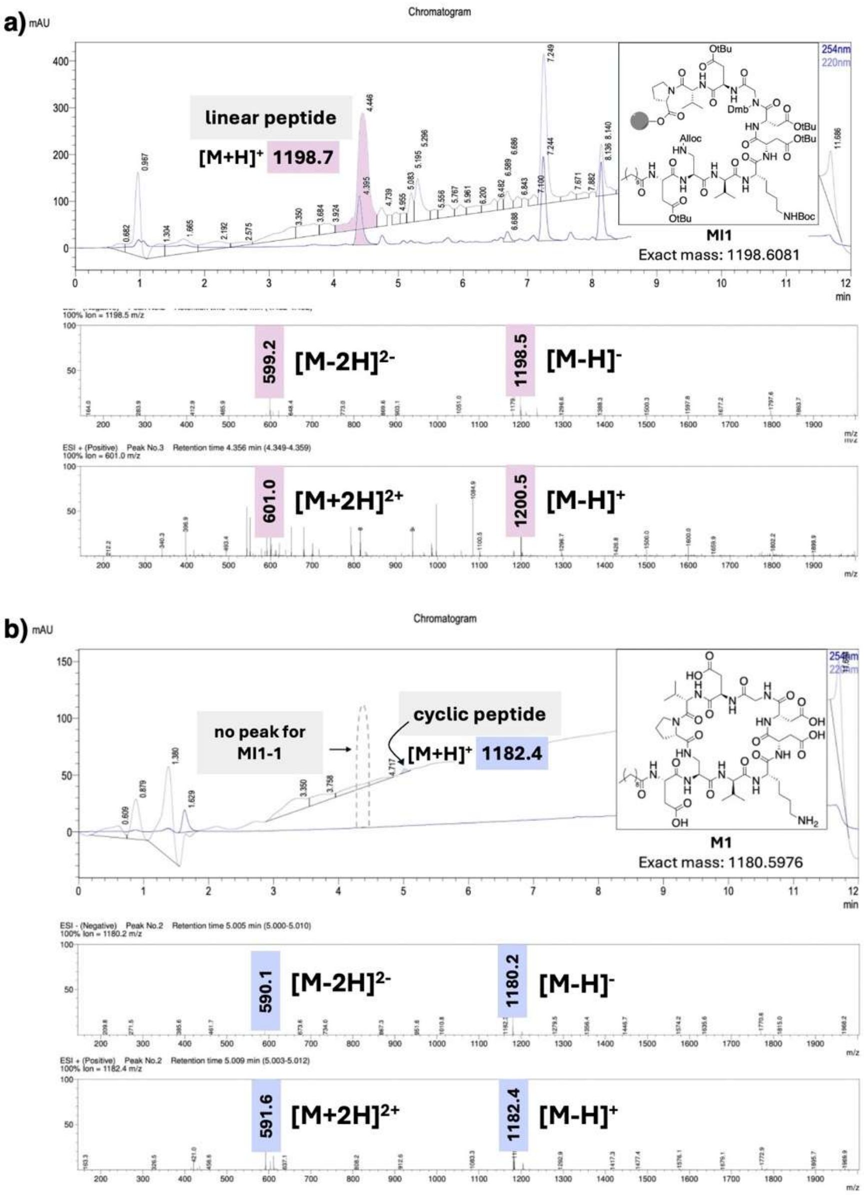

Figure S4: Malacidin synthetic route development, from H-Pro-2-CTC resin to **M1** (highlighted in blue) via **MI1** (highlighted in pink). Progression through the synthesis as observed by LC-MS. Other peaks visible in the chromatogram correspond to side products from the synthesis and were not determined in detail. **a)** LC-MS chromatogram and mass spectra (ESI+, ESI-) of **MI1** linear intermediate pre-cyclisation (Alloc-protected, resin cleaved, side-chain deprotected) at an exact mass of 1198.6081 for $C_{52}H_{86}N_{12}O_{20}$; Expected: $[M+H]^+$ 1999.6 *m/z*, $[M+2H]^{2+}$ at 600.3 *m/z*, $[M-H]^-$ at 1197.6 *m/z* and $[M-2H]^{2-}$ at 598.3 *m/z*; Found: $[M+H]^+$ 1200.5 *m/z*, $[M+2H]^{2+}$ at 601.0 *m/z*, $[M-H]^-$ at 1198.5 *m/z* and $[M-2H]^{2-}$ at 599.2 *m/z*. **b)** LC-MS chromatogram mass spectra (ESI+, ESI-) of cyclised product **M1** at an exact mass of 1180.5976 for $C_{52}H_{84}N_{12}O_{19}$; Expected: $[M+H]^+$ 1181.6 *m/z*, $[M+2H]^{2+}$ 591.3, $[M-H]^-$ 1179.6 *m/z*, $[M-2H]^{2-}$ 589.3 *m/z*; Found: $[M+H]^+$ 1182.4 *m/z*, $[M+2H]^{2+}$ 591.6 *m/z*, $[M-H]^-$ 1180.2 *m/z*, $[M-2H]^{2-}$ 590.1 *m/z*.

Table S1: Overview of the structural features of selected CDAs, including numbers of amino acids within the macrocycle, number of glycine residues within the macrocycle, whether the peptide bears the canonical calcium-binding motif, and the reported MIC.

CDA	Ring size (nr. of AA)	Nr. of Gly in ring	Binding Motif	MIC [µg/mL]
Daptomycin	10	2	Canonical	0.5 [1]–[4]
A54145	10	1	Canonical	1-2 [5]
Tsushimycin	10	2	Canonical	1-4 [4]
Laspartomycin	10	2	Canonical	2 [6]
Friulimicin	10	2	Canonical	0.078 [7]
Taromycin	10	2	Canonical	3.1 [8]
Amphomycin	10	2	Canonical	2 [9], [10]
Dilarmycin	9	3	Modified	3-6 [11]
Ambocidin	9	3	Modified	0.5-1 [12]
Olikomycin	9	3	Modified	2 [13]
Viennamycin	7	2	Modified	1 [14]
Malacidin	9	1	Modified	0.2-0.8 [15]
Cadaside	9	1	Modified	1-4 [16]

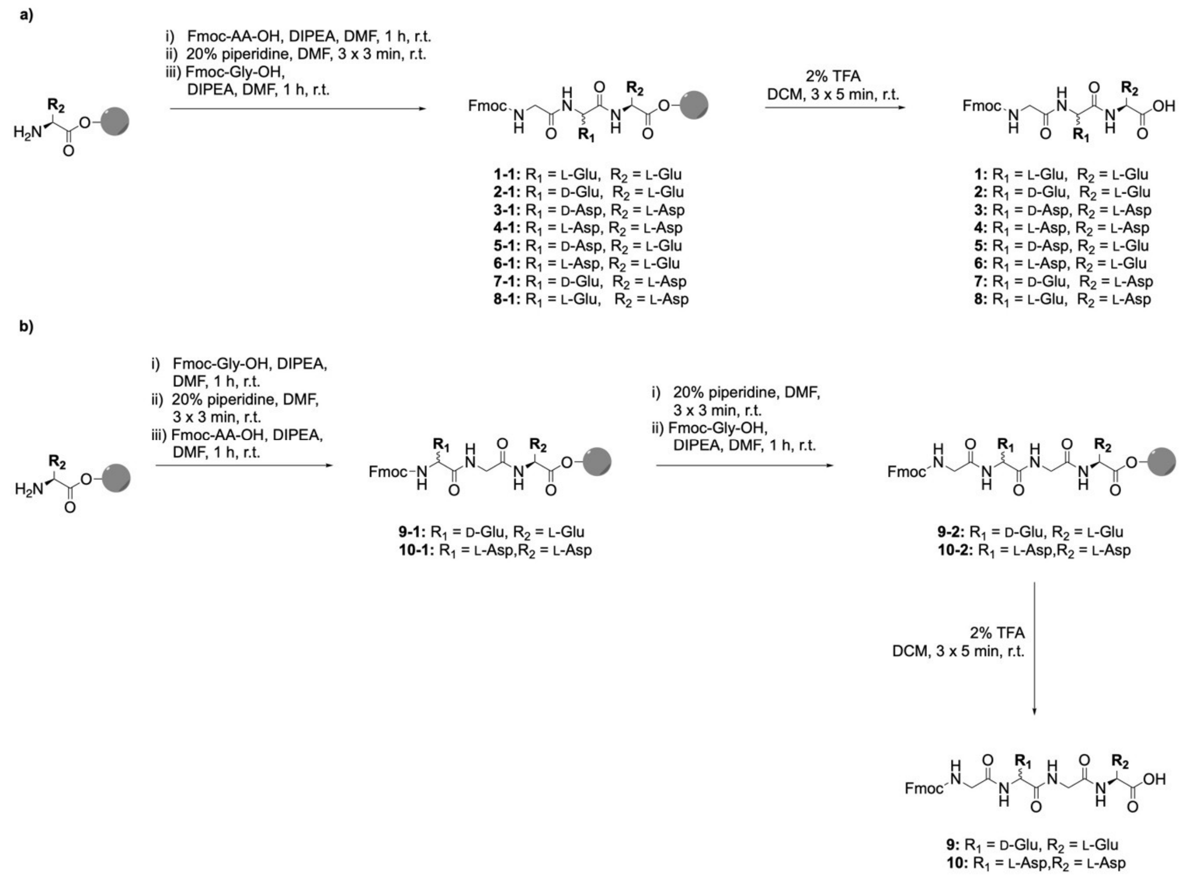

Scheme S2: Preparation of tri- and tetrapeptides required for synthesis of cadaside analogues in this work. **a)** SPPS of tripeptide building blocks **1-8** for synthesis of cadaside analogues **C0-C8**. **b)** SPPS of tetrapeptide building blocks **9-10** for synthesis of cadaside analogues **C9-C10**.

Table S2: Summary of the ability of CDAs to bind cell wall synthesis precursors, as well as tendency to induce membrane depolarisation. "+" indicates binding, "-" indicates no binding, "n.d." indicates binding has not been determined. * indicates binding occurs in the presence of phosphatidylglycerol.

Peptide	C_{55}-P	C_{55}-PP	Lipid I	Lipid II	Membrane Depolarisation
Daptomycin [1]–[4]	+ *	+ *	n.d.	+ *	+
A54145 [5]	n.d.	n.d.	n.d.	n.d.	+
Tsushimycin [4]	+	n.d.	n.d.	n.d.	n.d.
Laspartomycin [6]	+	n.d.	n.d.	n.d.	-
Friulimicin [7]	+	-	-	-	-
Taromycin	n.d.	n.d.	n.d.	n.d.	n.d.
Amphomycin [9], [10]	+	-	-	-	-
Dilarmycin	n.d.	n.d.	n.d.	n.d.	n.d.
Ambocidin [12]	-	-	n.d.	+	-
Olikomycin	n.d.	n.d.	n.d.	n.d.	n.d.
Viennamycin	n.d.	n.d.	n.d.	n.d.	n.d.
Malacidin [15]	+	n.d.	n.d.	+	-
Cadaside [16]	n.d.	n.d.	n.d.	n.d.	-

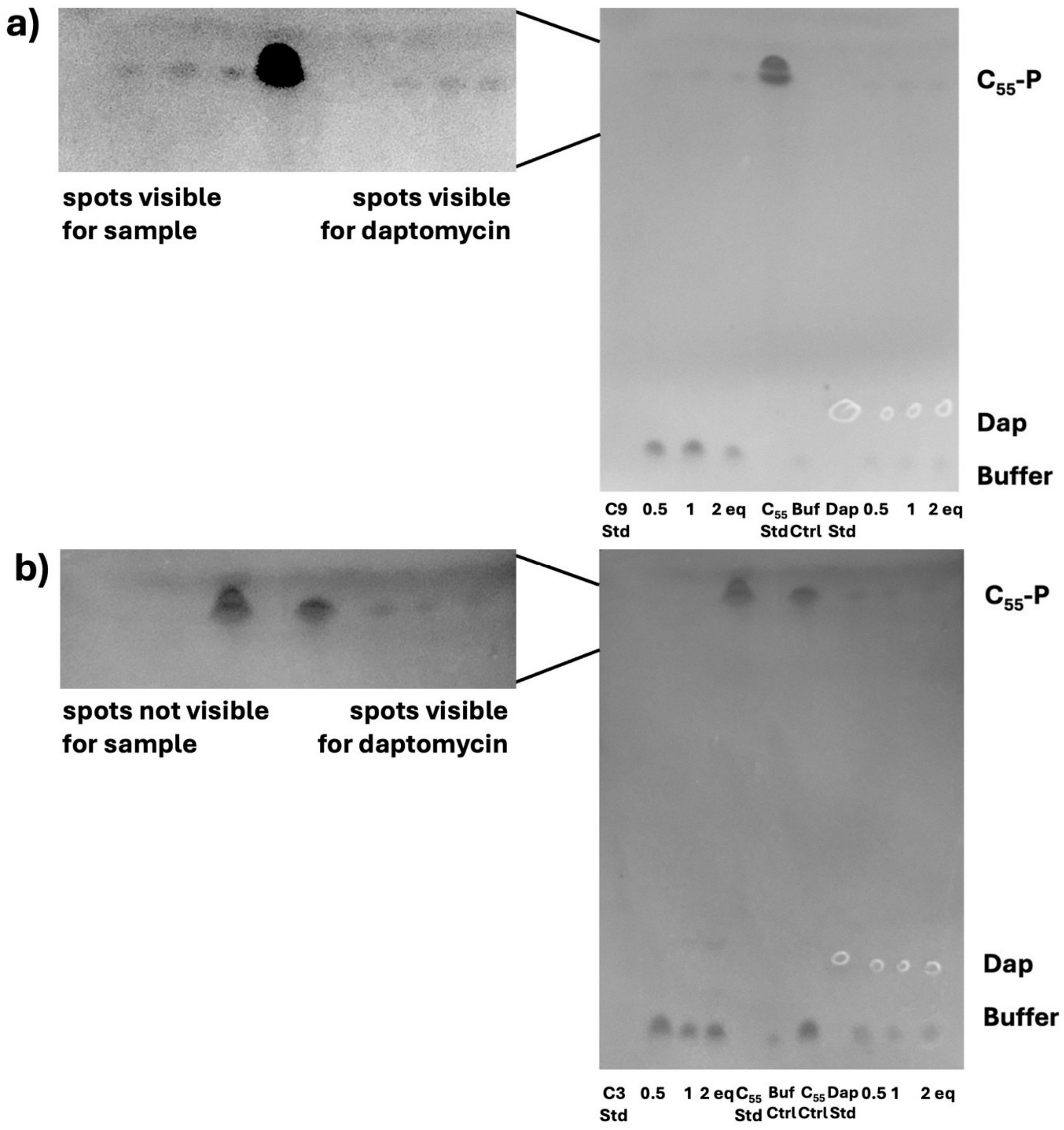

Figure S5: TLC binding assays of selected cadaside and malacidin analogues, showcasing retention times and staining on silica plates in a $CHCl_3$/MeOH/H_2O/NH_3 (88/48/10/1 *v/v*) solvent system. The solvent front and origin are at the top (cropped) and bottom of the images, respectively. A zoomed in section of binding spots is inset. TLC binding assays against C_{55}-P include a C_{55}-P standard (C_{55}-P in methanol) and control (C_{55}-P extracted from ^{t}BuOH/6 M PyOAc), as well as the peptide and daptomycin standards (peptide or daptomycin in water) and incubated samples with the precursor at 0.5, 1 and 2 equiv.. All plates also include a buffer control standard (buffer containing no peptide/lipid extracted from ^{t}BuOH/6 M PyOAc). **a)** Negative example **C9** against C_{55}-P with $KMnO_4$ staining (colour inverted), showcasing spots corresponding to C_{55}-P at Rf 0.8 after incubation with **C9** and daptomycin, indicating a lack of binding. **b)** Positive example **C3** against C_{55}-P with $KMnO_4$ staining (colour inverted), showcasing spots corresponding to C_{55}-P at Rf 0.82 after incubation with daptomycin, indicating a lack of binding, but no spots for C_{55}-P after incubation with the peptide, a potential indicator for binding.

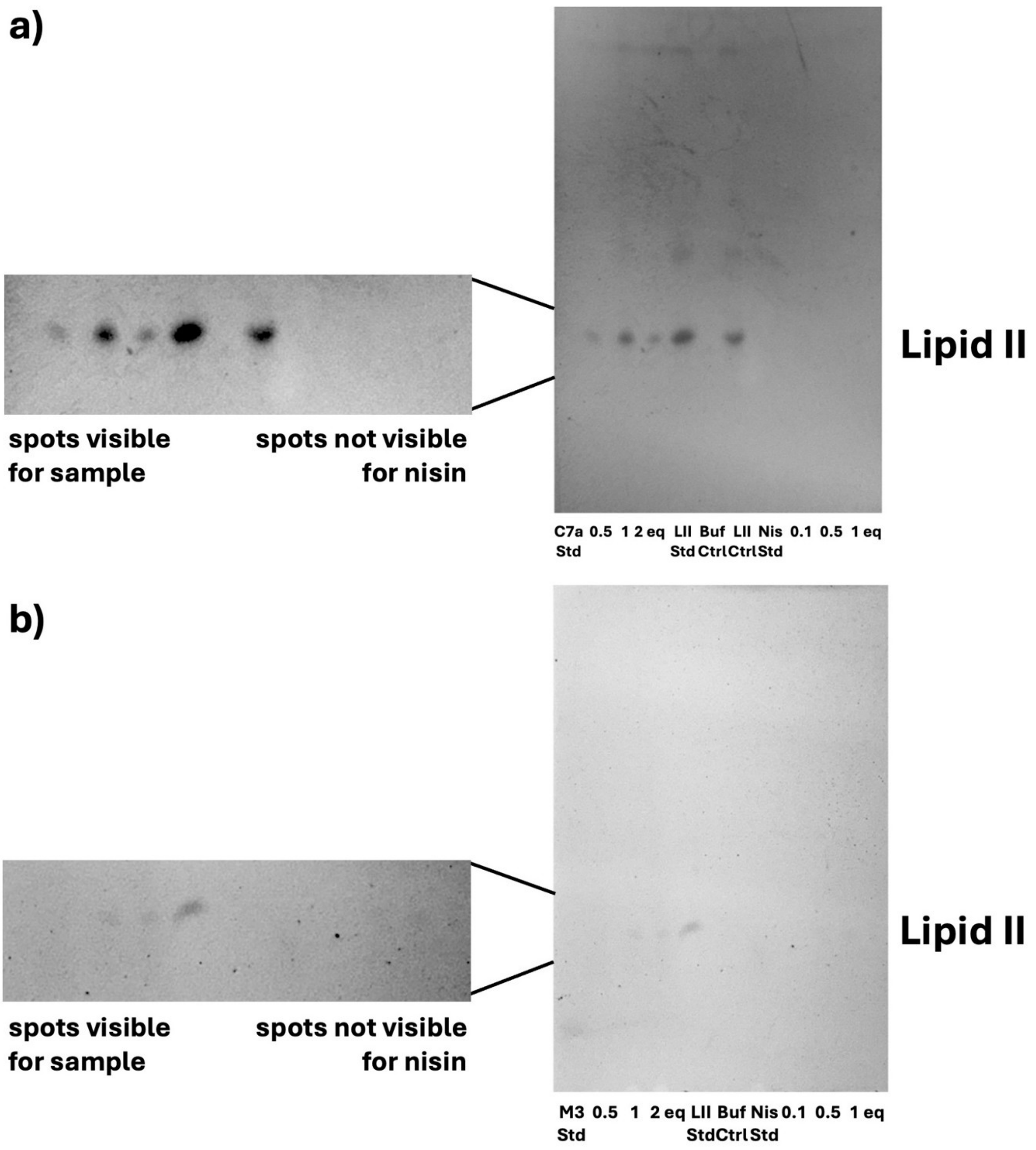

Figure S6: TLC binding assays of selected cadaside and malacidin analogues, showcasing retention times and staining on silica plates in a $CHCl_3$/MeOH/H_2O/NH_3 (88/48/10/1 *v/v*) solvent system. The solvent front and origin are at the top (cropped) and bottom of the images, respectively. A zoomed in section of binding spots is inset. TLC binding assays against lipid II include a lipid II standard (lipid II in methanol) and control (lipid II extracted from ^{t}BuOH/6 M PyOAc), as well as the peptide and nisin standard (peptide or nisin in water) and incubated samples with the precursor at 0.1, 0.5, 1 and 2 equiv.. All plates also include a buffer control standard (buffer containing no peptide/lipid extracted from ^{t}BuOH/6 M PyOAc). **a)** Negative example **C7a** against lipid II with PMA staining, showcasing spots corresponding to lipid II at Rf 0.31, indicating a lack of binding to the peptide, while no lipid II spots were observed for the nisin positive control, indicating nisin-lipid II binding. **b)** Negative example **M3** against lipid II with PMA staining, showcasing spots corresponding to lipid II at Rf 0.31, indicating a lack of binding to the peptide, while no spots were observed for the nisin positive control, indicating nisin-lipid II binding.

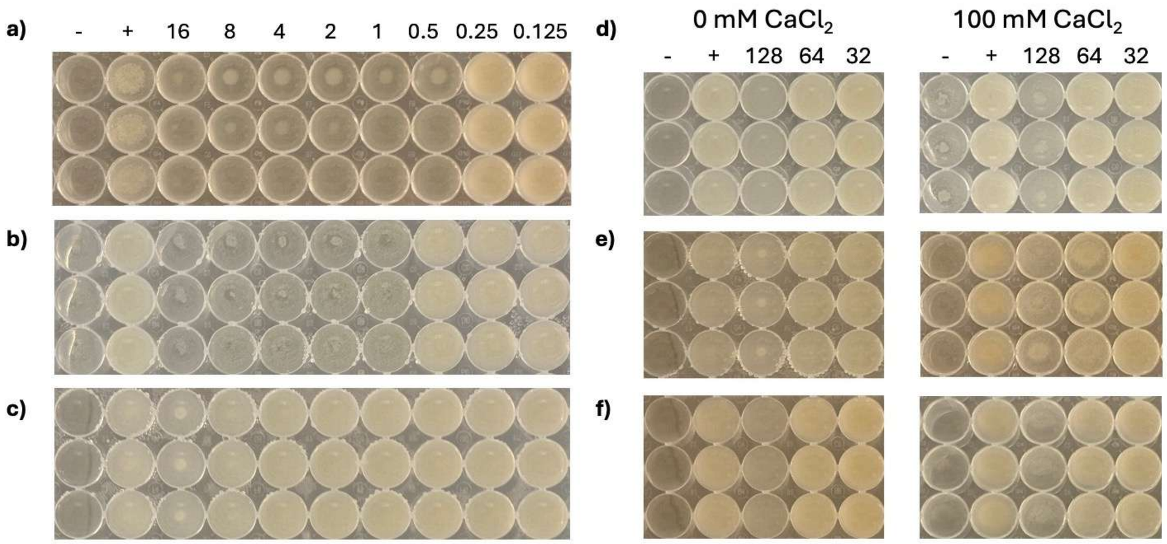

Figure S7: Visual MIC assay results against *B. subtilis* 168 in MHB II broth in triplicate, incubated over 24 h at 37 ˚C and 230 rpm. Each image includes at minimum three concentrations (128 µg/mL, 64 µg/mL, 32 µg/mL), a sterility control ("-"), and a growth control ("+"). **a)** Daptomycin reference in the presence of 100 mM Ca^{2+}. **b)** Daptomycin reference in the presence of 15 mM Ca^{2+}. **c)** Daptomycin reference in the absence of Ca^{2+}. **d)** Malacidin analogue **M5** in the absence and presence of 15 mM Ca^{2+}. **e)** Cadaside analogue **C5** in the absence and presence of 100 mM Ca^{2+}, showing decreased turbidity at the 64 µg/mL dilution step. **f)** Cadaside analogue **C6** in the absence presence of 100 mM Ca^{2+}, not showing decreased turbidity at the 64 µg/mL dilution step.

2. General procedures for peptide synthesis

General procedure 1 – Manual coupling of amino acids (standard and non-standard)
The resin (0.50-0.75 mmol, 1 equiv.) was washed with DMF (3 x 10 mL) and swelled in DMF (10 mL) for 15 min. To a solution of Fmoc-AA-OH (3 equiv.) and HATU (3 equiv.) in DMF (10 mL), DIPEA (6 equiv.) was added and activated for 2 min before adding it to the resin. The reaction was placed on a shaker for 1 h. For double couplings, this procedure was repeated once more. The resin was filtered and washed with DMF (4 x 10 mL).

General procedure 2 – Fmoc removal with piperidine
A solution of 20 *v/v*% piperidine in DMF (4 x 10 mL x 3 min) was added to the resin and agitated on a shaker. The resin was then filtered and washed with DMF (6 x 10 mL).

General procedure 3 – Fmoc removal with 2-methylpiperidine
A solution of 20 *v/v*% piperidine in DMF (4 x 10 mL x 3 min) was added to the resin and agitated on a shaker. The resin was then filtered and washed with DMF (6 x 10 mL).

General procedure 4 – On resin esterification with Fmoc-Sar-OH
A solution of Fmoc-Sar-OH (20 equiv.) in DMF (10 mL) and DIC (20 eq). was added to the resin without prior activation. A stock solution of DMAP (0.4 equiv.) in DMF was then added to the resin and the reaction was agitated on a shaker for 3 h. The resin was then filtered and the reaction repeated with the same conditions. The resin was the filtered and washed with methanol (3 x 10 mL) and DMF (3 x 10 mL).

General procedure 5 – Manual coupling of tri- or tetrapeptides
The on-resin esterified peptide was split into equal amounts of ~55 µmol to create cadaside analogues using the synthesised tri- or tetrapeptides (**1**-**10**). To a solution of the Fmoc-protected tri- or tetrapeptide (2-3 eq). and HATU (2-3 equiv.) in DMF (1 mL), DIPEA (4-6 equiv.) was added and activated for 2 min before adding it to the resin. The reaction was placed on a shaker overnight. The resin was filtered and washed with DMF (4 x 1 mL).

General procedure 6 – Alloc removal
The dried resin was transferred into a dry microwave tube, flushed with nitrogen and swelled in DMF/dichloromethane (1/1, 2 mL) for 15 min. Tetrakis(triphenylphosphine)palladium(0) (0.4 equiv.) was dissolved in DMF (2 mL) in a dry microwave tube, and flushed with nitrogen under a stream of nitrogen in the dark. Phenylsilane (10 equiv.) was added to the solution and mixed utilising a stream of nitrogen. The reagents were then added to the swelled resin and agitated on a shaker under nitrogen for 2 h. The resin was then filtered, washed with DMF (6 x 2 mL), sodium diethyldithiocarbamate (0.5 *w/v*% in DMF), DIPEA (0.5 *v/v*% in DMF) and DMF (6 x 2 mL).

General procedure 7 – Manual coupling of decanoic acid
To a solution of decanoic acid (3 equiv.) and HATU (3 equiv.) in DMF (2 mL), DIPEA (6 equiv.) was added and activated for 2 min before adding it to the resin. The reaction was placed on a shaker for 2 h. The resin was filtered and the reaction repeated under the same conditions. The resin was filtered and washed with DMF (4 x 2 mL).

General procedure 8 – Resin cleavage

The dry resin was washed with dichloromethane (3 x 2 mL) and swelled in dichloromethane (2 mL) for 15 min. A solution of 2 *v/v*% TFA in dichloromethane (3 x 2 mL x 5 min) was added to the resin and the reaction agitated on the shaker. The supernatant was collected and the peptide dried fully under a stream of nitrogen.

General procedure 9 – Microwave assisted cyclisation

The peptide was dissolved in anhydrous dichloromethane to a final peptide concentration of 6 mM in a dry microwave vial. HATU (1.2 equiv.) and DIPEA (6 equiv.) were added to the solution and the pH checked with indicator paper. On a Biotage® Initiator+ microwave system, the reaction was stirred at 600 pm at 70 ˚C for 1 h. The solution was then fully dried under a stream of nitrogen.

General procedure 10 – Side chain deprotection

The peptide was dissolved in dichloromethane (50 *v/v*% of total cleavage solution) and cooled to 0 ˚C. The cleavage cocktail TFA/TIPS/H_2O (45/2.5/2.5) was then added to the peptide solution and stirred for 5 min at 0 ˚C. The reaction was then allowed to cool to room temperature and stirred for an additional 45 min. The solution was then fully dried under a stream of nitrogen, before washing with cold diethyl ether (3 x 2 mL). The peptide was then dissolved in ACN/H_2O (1/1) and lyophilised.

General procedure 11 – Synthesis of cadaside analogues C1-C10

Preloaded resin HN-L-Pro-OCTC (loading = 0.82 mmol/g) was elongated using **General procedure 1** for coupling and **General Procedure 2** for Fmoc-deprotection reactions, to attach the first 7 amino acid residues. Each reaction step as outlined **Scheme S1** was followed by a mini cleavage using **General procedure 10** to enable LC-MS analysis prior to progression to the subsequent step. The ester was installed using **General procedure 4**, and the Fmoc group was removed via **General procedure 3**. The respective tri-/tetrapeptide (**01-10**) was then coupled using **General procedure 1**, with shaking overnight rather than for 1 h. The Alloc-protecting group was removed, following **General procedure 6**, after which the lipid tail was installed via **General procedure 7**. The remaining Fmoc-protecting group was cleaved using **General procedure 3**, before cleaving the peptide from the resin via **General procedure 8**. Macrocyclisation was achieved using the novel **General procedure 9**, followed by global deprotection in solution via **General procedure 10**. After lyophilisation, the peptides were afforded as a white to light yellow solid, which was dissolved in ACN/H_2O (1/1) and purified by prep-HPLC, on a 5-95% B gradient over 27 min (A = 0.1% TFA in H_2O, B = 0.1% TFA in ACN).

General procedure 12 – Synthesis of malacidin analogues M1-M5

Preloaded resin HN-L-Pro-OCTC (loading = 0.82 mmol/g) was elongated using **General procedure 1** for coupling and **General Procedure 2** for Fmoc-deprotection reactions, to attach all 9 or 10 amino acid residues. Decanoic acid was installed using **General Procedure 7**. The Alloc-protecting group was removed using **General procedure 6** and the peptide was cyclised according to **General procedure 9**. The final product was deprotected using **General procedure 10**. After lyophilisation, the peptides were afforded as a white to light yellow solid, which was dissolved in ACN/H_2O (1/1) and

purified by prep-HPLC, on a 5-95% B gradient over 27 min (A = 0.1% TFA in H_2O, B = 0.1% TFA in ACN).

Mass Calculation via Tyrosine Absorption at 280 nm

Masses obtained for low yield cadaside analogues were experimentally determined through measurement of tyrosine absorption at 280 nm. Dry samples were dissolved in 1 mL HPLC-grade H_2O. Where dissolution was not instant, the sample was treated in the sonicator for 10 seconds at a time until dissolved. Absorption for each sample was blanked against HPLC-grade H_2O and measured in duplicate. Concentration (μmol/mL) and subsequent absolute mass was calculated using the average of a previously established tyrosine calibration curve (0.05-1.5 μmol/mL, **Table S3** and **Figure S8**), as well as the result from the Beer Lambert law (Abs_{280} = absorption at 280 nm, l = path length) with the tyrosine extinction coefficient (ε) *at 1493* M^{-1} cm^{-1} [17].

Table S3: Measured absorbance values for tyrosine calibration curve.

c_{Tyr} [μmol/mL]	Abs_{280-1}	Abs_{280-2}	Abs_{280-av}
0.05	0.066	0.067	0.067
0.10	0.158	0.129	0.144
0.15	0.255	0.273	0.264
0.20	0.295	0.297	0.296
0.30	0.442	0.454	0.448
0.40	0.589	0.59	0.590
0.50	0.760	0.758	0.759
0.60	0.924	0.896	0.910
0.70	1.047	1.073	1.060
0.80	1.187	1.193	1.190
0.90	1.368	1.372	1.370
1.00	1.594	1.574	1.584
1.30	1.881	1.931	1.906
1.50	2.169	2.156	2.163

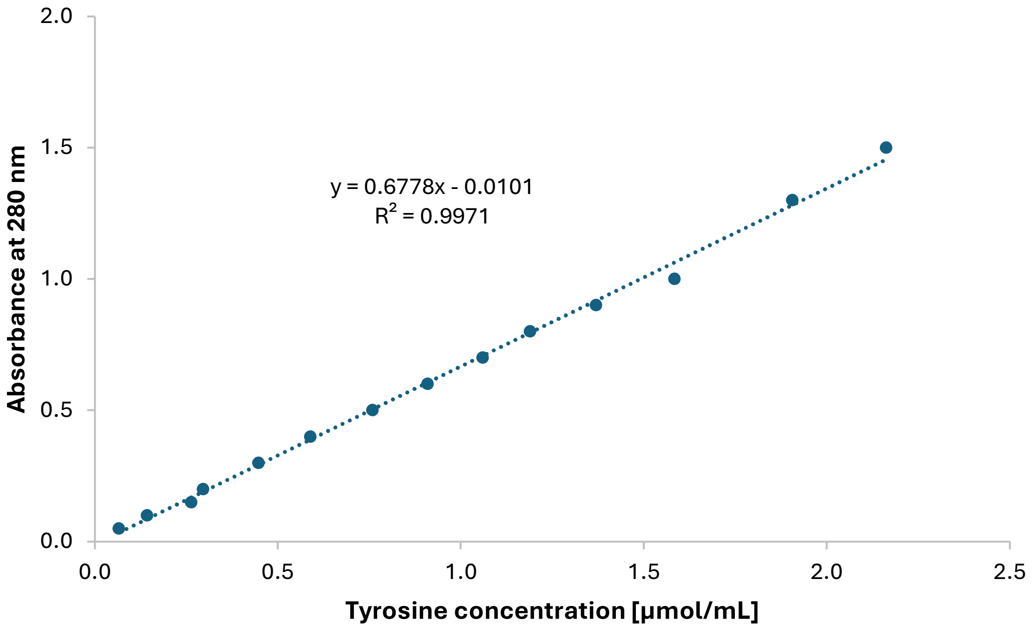

Figure S8: Tyrosine calibration curve.

Table S4: Concentration calculation of cadaside analogues **C1-C9** via absorbance measurement at 280 nm.

Peptide	Abs_{280}	*c* (Beer Lambert) [µmol/mL]	*c* (Calibration curve) [µmol/mL]	c_{av} [µmol/mL]	*V* [mL]	*n* [µmol]	*M* [mmol/mg]	*m* [mg]
C1a	0.437	0.2927	0.2861	0.29	1	0.29	1516.6210	0.19
C1b	0.308	0.2063	0.1987	0.20	1	0.20	1516.6210	0.13
C2a	0.845	0.5660	0.5626	0.56	1	0.56	1516.6210	0.37
C2b	0.367	0.2458	0.2387	0.24	1	0.24	1516.6210	0.16
C2c	0.353	0.2364	0.2292	0.23	1	0.23	1516.6210	0.15
C3	0.777	0.5204	0.5166	0.52	1	0.52	1488.5670	0.35
C4	0.945	0.6330	0.6304	0.63	1	0.63	1488.5670	0.42
C5	0.766	0.5131	0.5091	0.51	1	0.51	1502.5940	0.34
C6	0.478	0.3202	0.3139	0.32	1	0.32	1502.5940	0.21
C7a	0.849	0.5687	0.5654	0.57	1	0.57	1502.5940	0.38
C7b	0.355	0.2378	0.2305	0.23	1	0.23	1502.5940	0.15
C8	0.541	0.3624	0.3566	0.36	1	0.36	1502.5940	0.24
C9	0.478	0.3202	0.3139	0.32	1	0.32	1502.5940	0.21

3. Synthesis of Building Blocks

SPPS of Fmoc-Gly-L-Glu(O^{t}Bu)-L-Glu(O^{t}Bu)-OH (1)

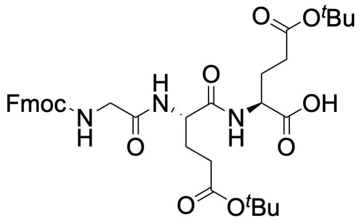

Tripeptide **1** was prepared using a preloaded H_2N-L-Glu(O^{t}Bu)-OCTC resin.

Table S5: Table of resin and amino acid quantities used per coupling step to yield tripeptide **1**.

**Amino acid**	***M* [g/mol]**	***m* [g]**	***n* [mmol]**	***V* [mL]**	***c* [M]**
**H_2N-L-Glu(O^{t}Bu)-OCTC**	0.79 mmol/g	0.34	0.26	-	-
**Fmoc-L-Glu(O^{t}Bu)-OH**	425.47	0.34	0.78	3.8	0.22
**Fmoc-Gly-OH**	297.31	0.22	0.78	3.8	0.22

The peptide was assembled using **General procedure 1** for coupling and **General procedure 2** for Fmoc-deprotection reactions, without *N*-terminal or side chain final deprotection. The peptide was cleaved off the resin following **General procedure 8**. The product was dried under a stream of nitrogen and washed with water and brine, before being dried over $MgSO_4$, filtered and the solvent evaporated under reduced pressure. The corresponding *m/z* in both LC-MS and HRMS analysis (mass error <1 ppm) confirmed the formation of the desired product (**Figure S10**). The final product, a white solid, was used without further purification (0.13 g, 0.20 mmol, crude 76%).

SPPS of Fmoc-Gly-D-Glu(O^{t}Bu)-L-Glu(O^{t}Bu)-OH (2)

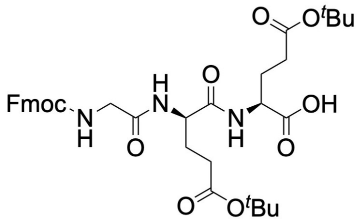

Tripeptide **2** was prepared using a preloaded H_2N-L-Glu(O^{t}Bu)-OCTC resin.

Table S6: Table of resin and amino acid quantities used per coupling step to yield tripeptide **2**.

**Amino acid**	***M* [g/mol]**	***m* [g]**	***n* [mmol]**	***V* [mL]**	***c* [M]**
**H_2N-L-Glu(O^{t}Bu)-OCTC**	0.79 mmol/g	0.33	0.26	-	-
**Fmoc-D-Glu(O^{t}Bu)-OH**	425.47	0.33	0.78	3.8	0.22
**Fmoc-Gly-OH**	297.31	0.23	0.78	3.8	0.22

The peptide was assembled using **General procedure 1** for coupling and **General procedure 2** for Fmoc-deprotection reactions, without *N*-terminal or side chain final deprotection. The peptide was cleaved off the resin following **General procedure 8**. The product was dried under a stream of nitrogen and washed with water and brine, before being dried over $MgSO_4$, filtered and the solvent evaporated under reduced pressure. The corresponding *m/z* in both LC-MS and HRMS analysis (mass error <1 ppm) confirmed the formation of the desired product (**Figure S11**). The final product, a white solid, was used without further purification (0.15 g, 0.22 mmol, crude 87%).

SPPS of Fmoc-Gly-D-Asp(O^{t}Bu)-L-Asp(O^{t}Bu)-OH (3)

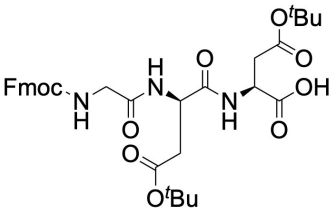

Tripeptide **3** was prepared using a preloaded H_2N-L-Asp(O^{t}Bu)-OCTC resin.

Table S7: Table of resin and amino acid quantities used per coupling step to yield tripeptide **3**.

Amino acid	*M* [g/mol]	*m* [g]	*n* [mmol]	*V* [mL]	*c* [M]
**H_2N-L-Asp(O^{t}Bu)-OCTC**	0.58 mmol/g	0.29	0.17	-	-
**Fmoc-D-Asp(O^{t}Bu)-OH**	411.45	0.21	0.51	2.6	0.22
**Fmoc-Gly-OH**	297.31	0.15	0.51	2.6	0.22

The peptide was assembled using **General procedure 1** for coupling and **General procedure 2** for Fmoc-deprotection reactions, without *N*-terminal or side chain final deprotection. The peptide was cleaved off the resin following **General procedure 8**. The product was dried under a stream of nitrogen and washed with water and brine, before being dried over $MgSO_4$, filtered and the solvent evaporated under reduced pressure. The corresponding *m/z* in both LC-MS and HRMS analysis (mass error <1 ppm) confirmed the formation of the desired product (**Figure S12**). The final product, a white solid, was used without further purification (89.1 mg, 0.14 mmol, crude 81%).

SPPS of Fmoc-Gly-L-Asp(O^{t}Bu)-L-Asp(O^{t}Bu)-OH (4)

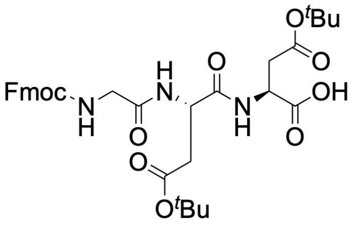

Tripeptide **4** was prepared using a preloaded H_2N-L-Asp(O^{t}Bu)-OCTC resin.

Table S8: Table of resin and amino acid quantities used per coupling step to yield tripeptide **4**.

**Amino acid**	***M* [g/mol]**	***m* [g]**	***n* [mmol]**	***V* [mL]**	***c* [M]**
**H_2N-L-Asp(O^{t}Bu)-OCTC**	0.58 mmol/g	0.30	0.17	-	-
**Fmoc-L-Asp(O^{t}Bu)-OH**	411.45	0.21	0.51	2.6	0.22
**Fmoc-Gly-OH**	297.31	0.16	0.51	2.6	0.22

The peptide was assembled using **General procedure 1** for coupling and **General procedure 2** for Fmoc-deprotection reactions, without *N*-terminal or side chain final deprotection. The peptide was cleaved off the resin following **General procedure 8**. The product was dried under a stream of nitrogen and washed with water and brine, before being dried over $MgSO_4$, filtered and the solvent evaporated under reduced pressure. The corresponding *m/z* in both LC-MS and HRMS analysis (mass error <1 ppm) confirmed the formation of the desired product (**Figure S13**). The final product, a white solid, was used without further purification (91.1 mg, 0.14 mmol, crude 83%).

SPPS of Fmoc-Gly-D-Asp(O^{t}Bu)-L-Glu(O^{t}Bu)-OH (5)

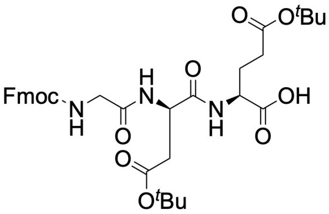

Tripeptide **5** was prepared using a preloaded H_2N-L-Glu(O^{t}Bu)-OCTC resin.

Table S9: Table of resin and amino acid quantities used per coupling step to yield tripeptide **5**.

**Amino acid**	***M* [g/mol]**	***m* [g]**	***n* [mmol]**	***V* [mL]**	***c* [M]**
**H_2N-L-Glu(O^{t}Bu)-OCTC**	0.79 mmol/g	0.22	0.17	-	-
**Fmoc-D-Asp(O^{t}Bu)-OH**	411.45	0.22	0.52	2.6	0.22
**Fmoc-Gly-OH**	297.31	0.16	0.51	2.6	0.22

The peptide was assembled using **General procedure 1** for coupling and **General procedure 2** for Fmoc-deprotection reactions, without *N*-terminal or side chain final deprotection. The peptide was cleaved off the resin following **General procedure 8**. The product was dried under a stream of nitrogen and washed with water and brine, before being dried over $MgSO_4$, filtered and the solvent evaporated under reduced pressure. The corresponding *m/z* in both LC-MS and HRMS analysis (mass error <1 ppm) confirmed the formation of the desired product (**Figure S14**). The final product, a white solid, was used without further purification (59.1 mg, 0.10 mmol, crude 77%).

SPPS of Fmoc-Gly-L-Asp(O^{t}Bu)-L-Glu(O^{t}Bu)-OH (6)

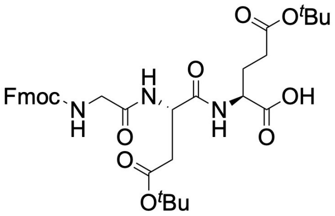

Tripeptide **6** was prepared using a preloaded H_2N-L-Glu(O^{t}Bu)-OCTC resin.

Table S10: Table of resin and amino acid quantities used per coupling step to yield tripeptide **6**.

**Amino acid**	***M* [g/mol]**	***m* [g]**	***n* [mmol]**	***V* [mL]**	***c* [M]**
**H_2N-L-Glu(O^{t}Bu)-OCTC**	0.79 mmol/g	0.23	0.18	-	-
**Fmoc-L-Asp(O^{t}Bu)-OH**	411.45	0.25	0.56	3.0	0.22
**Fmoc-Gly-OH**	297.31	0.18	0.56	3.0	0.22

The peptide was assembled using **General procedure 1** for coupling and **General procedure 2** for Fmoc-deprotection reactions, without *N*-terminal or side chain final deprotection. The peptide was cleaved off the resin following **General procedure 8**. The product was dried under a stream of nitrogen and washed with water and brine, before being dried over $MgSO_4$, filtered and the solvent evaporated under reduced pressure. The corresponding *m/z* in both LC-MS and HRMS analysis (mass error <1 ppm) confirmed the formation of the desired product (**Figure S15**). The final product, a white solid, was used without further purification (102.4 mg, 0.16 mmol, crude 86%).

SPPS of Fmoc-Gly-D-Glu(O^{t}Bu)-L-Asp(O^{t}Bu)-OH (7)

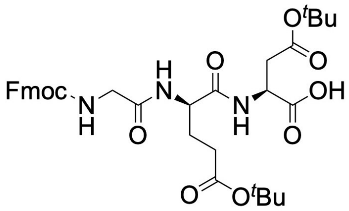

Tripeptide **7** was prepared using a preloaded H_2N-L-Asp(O^{t}Bu)-OCTC resin.

Table S11: Table of resin and amino acid quantities used per coupling step to yield tripeptide **7**.

**Amino acid**	***M* [g/mol]**	***m* [g]**	***n* [mmol]**	***V* [mL]**	***c* [M]**
**H_2N-L-Asp(O^{t}Bu)-OCTC**	0.58 mmol/g	0.30	0.17	-	-
**Fmoc-D-Glu(O^{t}Bu)-OH**	425.47	0.22	0.52	2.6	0.22
**Fmoc-Gly-OH**	297.31	0.16	0.51	2.6	0.22

The peptide was assembled using **General procedure 1** for coupling and **General procedure 2** for Fmoc-deprotection reactions, without *N*-terminal or side chain final deprotection. The peptide was cleaved off the resin following **General procedure 8**. The product was dried under a stream of nitrogen and washed with water and brine, before being dried over $MgSO_4$, filtered and the solvent evaporated under reduced pressure. The corresponding *m/z* in both LC-MS and HRMS analysis (mass error <1 ppm) confirmed the formation of the desired product (**Figure S16**). The final product, a white solid, was used without further purification (102.1 mg, 0.16 mmol, crude 90%).

SPPS of Fmoc-Gly-L-Glu(O^{t}Bu)-L-Asp(O^{t}Bu)-OH (8)

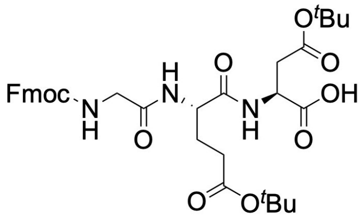

Tripeptide **8** was prepared using a preloaded H_2N-L-Asp(O^{t}Bu)-OCTC resin.

Table S12: Table of resin and amino acid quantities used per coupling step to yield tripeptide **8**.

**Amino acid**	***M* [g/mol]**	***m* [g]**	***n* [mmol]**	***V* [mL]**	***c* [M]**
**H_2N-L-Asp(O^{t}Bu)-OCTC**	0.58 mmol/g	0.22	0.13	-	-
**Fmoc-L-Glu(O^{t}Bu)-OH**	425.47	0.14	0.37	2.0	0.22
**Fmoc-Gly-OH**	297.31	0.12	0.38	2.0	0.22

The peptide was assembled using **General procedure 1** for coupling and **General procedure 2** for Fmoc-deprotection reactions, without *N*-terminal or side chain final deprotection. The peptide was cleaved off the resin following **General procedure 8**. The product was dried under a stream of nitrogen and washed with water and brine, before being dried over $MgSO_4$, filtered and the solvent evaporated under reduced pressure. The corresponding *m/z* in both LC-MS and HRMS analysis (mass error <1 ppm) confirmed the formation of the desired product (**Figure S17**). The final product, a white solid, was used without further purification (81.2 mg, 0.12 mmol, crude 95%).

SPPS of Fmoc-Gly-D-Glu(O^{t}Bu)-Gly-L-Glu(O^{t}Bu)-OH (9)

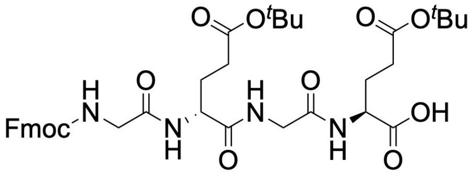

Tetrapeptide **9** was prepared using a preloaded H_2N-L-Glu(O^{t}Bu)-OCTC resin.

Table S13: Table of resin and amino acid quantities used per coupling step to yield tetrapeptide **9**.

**Amino acid**	***M* [g/mol]**	***m* [g]**	***n* [mmol]**	***V* [mL]**	***c* [M]**
**H_2N-L-Glu(O^{t}Bu)-OCTC**	0.79 mmol/g	0.34	0.22	-	-
**Fmoc-Gly-OH**	297.31	0.19	0.55	2.8	0.22
**Fmoc-D-Glu(O^{t}Bu)-OH**	425.47	0.28	0.65	2.8	0.22
**Fmoc-Gly-OH**	297.31	0.19	0.55	2.8	0.22

The peptide was assembled using **General procedure 1** for coupling and **General procedure 2** for Fmoc-deprotection reactions, without *N*-terminal or side chain final deprotection. The peptide was cleaved off the resin following **General procedure 8**. The product was dried under a stream of nitrogen and washed with water and brine, before being dried over $MgSO_4$, filtered and the solvent evaporated under reduced pressure. The corresponding *m/z* in both LC-MS and HRMS analysis (mass error <1 ppm) confirmed the formation of the desired product (**Figure S18**).The final product, a white solid, was used without further purification (124.1 mg, 0.17 mmol, crude 79%).

SPPS of Fmoc-Gly-L-Asp(O^{t}Bu)-(Dmb)Gly-L-Asp(O^{t}Bu)-OH (10)

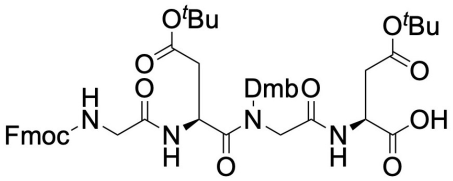

Tetrapeptide **10** was prepared using a preloaded H_2N-L-Asp(O^{t}Bu)-OCTC resin.

Table S14: Table of resin and amino acid quantities used per coupling step to yield tetrapeptide **10**.

Amino acid	*M* [g/mol]	*m* [g]	*n* [mmol]	*V* [mL]	*c* [M]
**H_2N-L-Asp(O^{t}Bu)-OCTC**	0.58 mmol/g	0.30	0.17	-	-
**Fmoc- L-Asp(O^{t}Bu)-(Dmb)Gly-OH**	297.31	0.34	0.53	2.8	0.22
**Fmoc-Gly-OH**	297.31	0.16	0.53	2.8	0.22

The peptide was assembled using **General procedure 1** for coupling and **General procedure 2** for Fmoc-deprotection reactions, without *N*-terminal or side chain final deprotection. The peptide was cleaved off the resin following **General procedure 8**. The product was dried under a stream of nitrogen and washed with water and brine, before being dried over $MgSO_4$, filtered and the solvent evaporated under reduced pressure. The corresponding *m/z* in both LC-MS and HRMS analysis (mass error <1 ppm) confirmed the formation of the desired product (**Figure S19**). The final product, a white solid, was used without further purification (87.8 mg, 0.13 mmol, crude 72%).

(3*S*)-3-(allyloxycarbonyl)amino-2-methylpropanoic acid (11) (Alloc-L-BAIBA-OH)

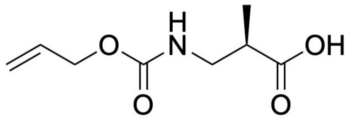

H_2N-L-*β*-amino isobutyric acid-OH (0.23 g, 1.77 mmol, 1 eq) and Na_2CO_3 (0.22 g, 1.90 mmol, 1 eq) were dissolved in water (3.5 mL) and allyl chloroformate (0.57 mL, 2.13 mmol, 1.2 eq) in dioxane (3.5 mL) was added. The mixture was stirred at room temperature for 48 h. The reaction was diluted with water (15 mL) and acidified with HCl (2.0 M) to a pH ~2. The organic phase was extracted with dichloromethane (3 x 30 mL) and the combined organic phase washed with water (4 x 30 mL), dried over $MgSO_4$, filtered and the solvent evaporated under reduced pressure. The product was then dry loaded onto silica gel and purified using the Biotage Selekt with a Biotage Sfär Silica Duo (60 µm, 25 g) flash column with a dichloromethane/methanol solvent system. The pure fractions were pooled, and the solvent removed under reduced pressure, yielding the product **10** as a light-yellow oil (0.17 g, 0.91 mmol, 51% yield). $[a]_D^{25}$ -19 (*c* = 1, $CHCl_3$);

^1H (CD_3OD, 400 MHz): δ 5.99-5.89 (m, 2H, CH_2=C**H**-), 5.31 (dd, 1H, *J* = 17.27 Hz, *J* = 15.92 Hz, C**H₂**=CH-), 5.19 (dd, 1H,*J* = 10.51 Hz, *J* = 9.61 Hz, C**H₂**=CH-), 4.54 (d, 2H, *J* = 5.37 Hz, CH_2=CH-C**H₂**-), 3.35 (dd, 1H, *J* = 13.70 Hz, *J* = 7.33 Hz, -NH-C**H₂**-), 3.20 (dd, 1H,*J* = 13.76 Hz, *J* = 6.49 Hz, -NH-C**H₂**-), 2.65 (sx, 1H, *J* = 6.89 Hz, -C**H**-CH_3), 1.17 (d, 3H, *J* = 7.11 Hz, -CH-C**H₃**);

^{13}C (CD_3OD, 100 MHz): δ 177.11 (-**C**O-O-), 157.38 (-NH-**C**O-), 130.06 (-**C**H=CH_2), 116.01 (-CH=**C**H_2), 64.096 (-**C**H_2-CH=), 43.21 (-**C**H_2-NH-), 39.64 (-CH_2-**C**H-), 13.70 (-**C**H_3-);

LCMS (ESI+ and ESI-) spectrum at 2.906-2.911 of **10**, *m/z* (ESI-MS) calculated for $C_8H_{13}NO_4$ at an exact mass of 187.0845: $[M+H]^+$ 188.1 *m/z*, $[M+ACN+H]^+$ 229.1 *m/z*, $[M-H]^-$ 186.1 *m/z*. Found: $[M+H]^+$ 187.5 *m/z*, $[M+ACN+H]^+$ 228.5 *m/z,* $[M-H]^-$ 186.5 *m/z*; HRMS (ESI+) spectrum of **10**; *m/z* (ESI+) calculated for $C_8H_{13}NO_4$ at an exact mass of 187.0845: $[M+H]^+$ 188.0918 *m/z*. Found: $[M+H]^+$ 188.0915 *m/z*, mass error <1 ppm.

3-[(2-(trimethylsilyl)ethoxy)carbonyl]amino-2-methylpropanoic acid (12) (Teoc-L-BAIBA-OH)

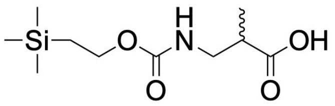

H_2N-D/L-*β*-amino isobutyric acid-OH (0.50 g, 3.80 mmol, 1 eq) was dissolved in water (3.50 mL) and triethylamine (0.80 mL, 5.74 mmol, 1.5 eq) was added in dioxane (4.5 mL). To the solution, Teoc-OSu (1.08 g, 4.18 mmol, 1.1 eq) was added. The mixture was stirred at room temperature overnight. The reaction was diluted with water (15 mL) and acidified with HCl (2.0 M) to a pH ~2. The organic phase was extracted with diethyl ether (3 x 50 mL) and the organic extracts washed with water (4 x 60 mL), dried over $MgSO_4$, filtered and the solvent evaporated under reduced pressure. The product was then dry loaded onto silica gel and purified using the Biotage Selekt with a Biotage Sfär Silica Duo (60 µm, 25 g) flash column with a dichloromethane/EtOAc solvent system. The pure fractions were pooled, and the solvent removed under reduced pressure, yielding the product **04** as a light-yellow oil (0.65 g, 2.64 mmol, 69% yield).

^{1}H ($CDCl_3$, 400 MHz): δ 8.68 (s, 1H, -CO-O**H**), 6.32 (s, 0.3H, -N**H**-), 5.38 (s, 0.7H, -N**H**-), 4.12-4.03 (m, 2H, -Si-CH_2-C**H₂**-), 3.30-3.17 (m, CH-C**H₂**-), 2.61 (s, 1H, -CH_2-C**H**-), 1.11 (d, 3H, *J* = 7.35 Hz, -CH-C**H₃**), 0.94-0.86 (m, 2H, -Si-C**H₂**-), -0.06 (s, 9H, (C**H₃**)$_3$-Si-);

^{13}C ($CDCl_3$, 100 MHz): δ 180.41 (-**C**O-OH), 157.13 (-NH-**C**O-O-), 63.40 (-Si-CH_2-**C**H_2-), 43.16 (-NH-**C**H_2-), 39.98 (-CH_2-**C**H-), 17.83 (Si-**C**H_2-CH_2-), 14.73 (-CH-**C**H_3), -1.37 ((**C**H_3)$_3$-Si-);

LCMS (ESI+ and ESI-) spectrum at 0.289-0.320 of **04**, *m/z* (ESI-MS) calculated for $C_{10}H_{21}NO_4Si$ at an exact mass of 247.1240: $[M+H]^+$ 248.1 *m/z*, $[2M+ACN+H]^+$ 311.1 *m/z*, $[2M+Na]^+$ 517.2 *m/z*, $[M-H]^-$ 246.1 *m/z*, $[2M-H]^-$ 493.2 *m/z*. Found: $[M-H]^-$ 246.3 *m/z*, $[2M+ACN+H]^+$ 311.4 *m/z*, $[2M-H]^-$ 492.5 *m/z*, $[2M+Na]^+$ 517.0 *m/z*; HRMS (ESI+) spectrum of **04**; *m/z* calculated for $C_{10}H_{21}NO_4Si$ at an exact mass of 247.1240: $[M+Na]^+$ 270.1092 *m/z*. Found: $[M+Na]^+$ 270.1132 *m/z*, mass error <1 ppm.

4. Total Synthesis of Cadaside analogues

Table S15: Summary of isolated yields, analytical HPLC purities, analytical HPLC retention times and mass errors of cadaside analogues synthesised in this work. *Determined only by gravimetric weighing.

	Yield [%]	Yield [mg]	Purity [%]	Retention time [min]	Mass error [ppm]
**C1a**	0.31	0.19	94	24.912	<1
**C1b**	0.19	0.13	98	25.125	<1
**C2a**	0.60	0.37	99	25.069	<1
**C2b**	0.20	0.16	98	25.311	<1
**C2c**	0.19	0.15	97	25.376	<1
**C3**	0.31	0.35	100	24.776	<1
**C4**	0.37	0.42	100	24.943	<1
**C5**	0.30	0.34	100	25.053	<1
**C6**	0.18	0.21	100	24.651	<1
**C7a**	0.33	0.38	100	24.893	<1
**C7b**	0.13	0.15	100	24.711	<1
**C8**	0.21	0.24	100	24.893	<1
**C9**	0.33	0.21	100	24.840	<1
**C10**	0.32	0.2*	99	24.564	n.d.

Cadaside Test Analogue C0

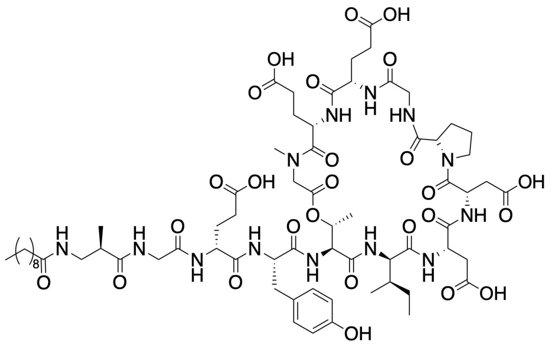

Table S16: Table of resin and amino acid quantities used per coupling step to yield cadaside analogue **C0**.

Amino acid	*M* [g/mol]	*m* [mg]	*n* [mmol]	*Equiv.*	*V* [mL]	*c* [M]
**HN-L-Pro-OCTC**	0.82 mmol/g	176.9	0.15	1	-	-
**Fmoc-L-Asp(O^{t}Bu)-OH**	439.50	181.7	0.44	3	2	0.2
**Fmoc-L-Asp(O^{t}Bu)-OH**	439.50	182.0	0.44	3	2	0.2
**Fmoc-D-Ile-OH**	353.41	155.1	0.44	3	2	0.2
**Fmoc-L-Thr-OH**	341.36	149.7	0.44	3	2	0.2
**Fmoc-L-Tyr(^{t}Bu)-OH**	459.53	206.0	0.44	3	2	0.2
**Fmoc-D-Glu(^{t}Bu)-OH**	425.47	188.9	0.44	3	2	0.2
**Fmoc-Gly-OH**	297.31	131.5	0.44	3	2	0.2
**Alloc-L-*β*Aib-OH (10)**	325.13	104.9	0.44	3	2	0.2
**Fmoc-Sar-OH**	311.33	906.3	1.46	3	2	0.7
**Tripeptide 01**	668.76	294.4	0.44	3	2	0.2
**Decanoic acid**	172.26	75.6	0.44	3	2	0.2

Preloaded resin HN-L-Pro-OCTC (loading = 0.82 mmol/g) was elongated using **General procedure 1** for coupling and **General procedure 2** for Fmoc-deprotection reactions, to attach the first 8 amino acid residues.

The ester was installed using **General procedure 4**, using Fmoc-Sar-OH (5.72 g, 18.4 mmol, 20 equiv.), DIC (2.9 mL, 18.4 mmol, 20 equiv.) and DMAP (45.2 mg, 0.37 mmol, 0.4 equiv.). Due to the volume of reagents, solvents and resin, this reaction was split among three reactors. Upon confirmation of the successful reaction, the Fmoc-removal was carried out via **General procedure 3**. We then followed **General procedure 11** to yield the final peptide. The peptide was successfully synthesised (**Figure S20**), but was not purified via prep-HPLC.

Preparation of Branched Precursor Cl2 for Cadaside Analogues C1-C10

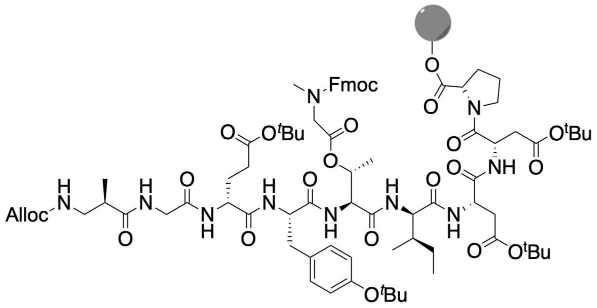

Table S17: Table of resin and amino acid quantities used per coupling step to yield the branched precursor for cadaside analogues **C1-C10**.

Amino acid	*M* [g/mol]	*m* [g]	*n* [mmol]	*Equiv.*	*V* [mL]	*c* [M]
**HN-L-Pro-OCTC**	0.82 mmol/g	1.09	0.92	1	-	-
**Fmoc-L-Asp(O^{t}Bu)-OH**	439.50	1.13	2.76	3	14	0.2
**Fmoc-L-Asp(O^{t}Bu)-OH**	439.50	1.13	2.76	3	14	0.2
**Fmoc-D-Ile-OH**	353.41	0.97	2.76	3	14	0.2
**Fmoc-L-Thr-OH**	341.36	0.94	2.76	3	14	0.2
**Fmoc-L-Tyr(^{t}Bu)-OH**	459.53	1.27	2.76	3	14	0.2
**Fmoc-D-Glu(^{t}Bu)-OH**	425.47	1.17	2.76	3	14	0.2
**Fmoc-Gly-OH**	297.31	0.82	2.76	3	14	0.2
**Alloc-L-βAib-OH (11)**	325.13	0.52	2.76	3	14	0.2

Preloaded resin HN-L-Pro-OCTC (loading = 0.82 mmol/g) was elongated using **General procedure 1** for coupling and **General procedure 2** for Fmoc-deprotection reactions, to attach the first 8 amino acid residues.

The ester was installed using **General procedure 4**, using Fmoc-Sar-OH (5.72 g, 18.4 mmol, 20 equiv.), DIC (2.9 mL, 18.4 mmol, 20 equiv.) and DMAP (45.2 mg, 0.37 mmol, 0.4 equiv.). Upon confirmation of the successful reaction, the Fmoc-removal was carried out via **General procedure 3** and analysed via LC-MS to confirm conversion (**Figure S9**). After this, the resin was split into aliquots for further reactions to yield cadaside analogues **C1-C10**.

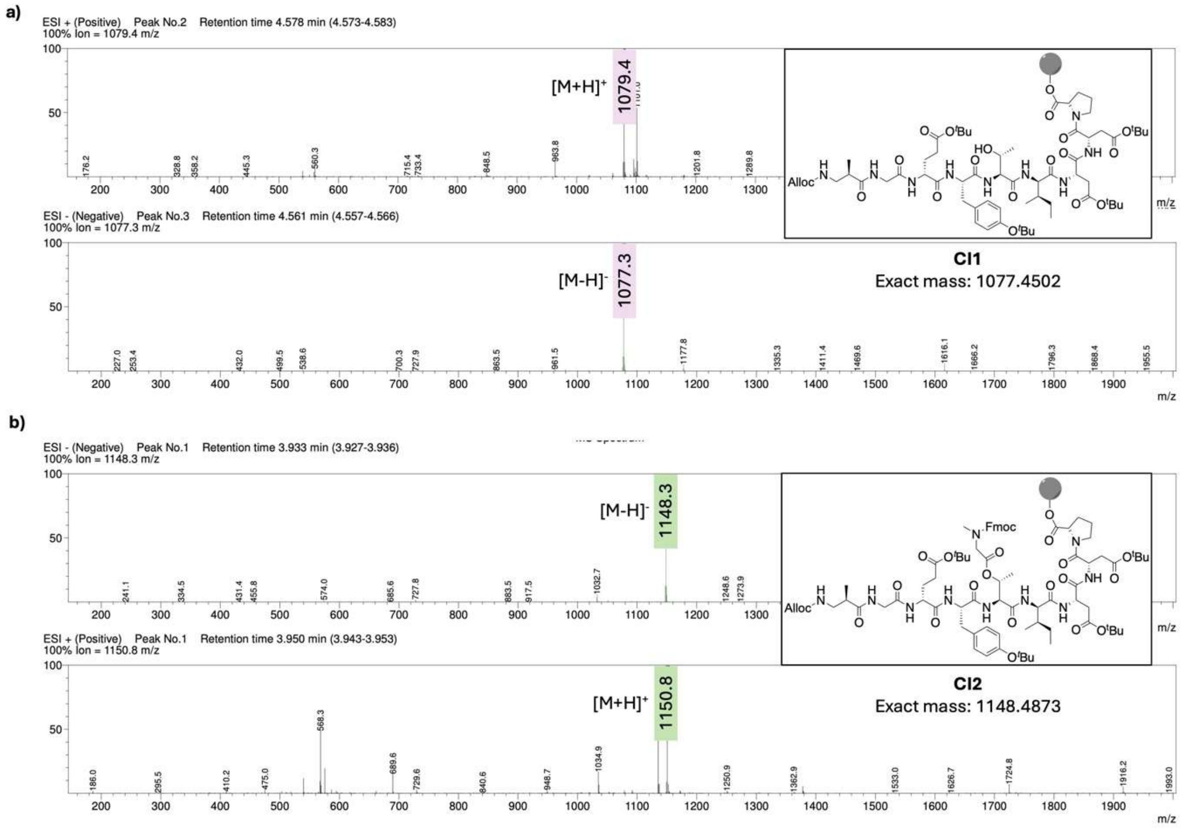

Figure S9: LC-MS analysis of cadaside branched precursors **CI1** and **CI2**. **a)** Mass spectra (ESI+, ESI-) of at 5.58 min of linear precursor peptide **CI1** (Alloc-protected, resin cleaved and side-chain deprotected) at an exact mass of 1077.4502 for $C_{47}H_{67}N_9O_{20}$; Expected: [M+H]$^+$ 1078.5 *m/z*, [M-H]$^-$ 1076.4 *m/z*; Found: [M+H]$^+$ 1079.4 *m/z*, [M-H]$^-$ 1077.3 *m/z*. **b)** Mass spectra (ESI+, ESI-) of at 5.58 min of branched precursor peptide **CI2** (Alloc-protected, resin cleaved and Fmoc- and side-chain deprotected) at an exact mass of 1148.4873 for $C_{50}H_{72}N_{10}O_{21}$; Expected: [M+H]$^+$ 1149.5 *m/z*, [M-H]$^-$ 1147.5 *m/z*; Found: [M+H]$^+$ 1150.8 *m/z*, [M-H]$^-$ 1148.3 *m/z*.

Synthesis of Analogues

SPPS of C1 (simplified cadaside)

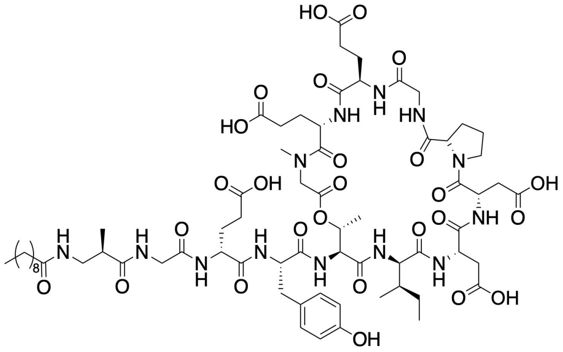

Table S18: Table of resin and amino acid quantities used per coupling step to yield cadaside analogue **C1**.

**Amino acid**	***M*** **[g/mol]**	***m*** **[mg]**	***n*** **[mmol]**	***Equiv.***	***V*** **[mL]**	***c*** **[M]**
**Precursor CI2**		45.9	0.04	1	-	-
**Tripeptide 2**	668.76	76.3	0.12	3	0.5	0.12
**Decanoic acid**	172.26	20.8	0.12	3	0.5	0.12

To synthesise cadaside analogue **C1** from precursor **CI2**, we employed **General Procedure 11** using residues as displayed in **Table S18**.

The peptide was successfully synthesised, and two isolates were collected by prep-HPLC on a 5-95% B gradient over 27 minutes (A = 0.1% TFA in H_2O, B = 0.1% TFA in ACN). Isolate **C1a** eluted at 13.7 min and isolate **C1b** at 14.0 min. HRMS of both samples confirmed the correct *m/z* corresponding to the exact mass of the peptide. Analytical HPLC determined purity at 94% for **C1a** (24.912 min) and 97% for **C1b** (25.125 min). Due to the low yield, the mass was calculated through **Mass Calculation via Tyrosine Absorption at 280 nm**.

This yielded pure **C1a** (0.19 mg, 0.12 µmol, 0.31%) and **C1b** (0.13 µg, 0.08 µmol, 0.19%) as white solids.

SPPS of C2 (simplified cadaside, D-Glu11 → L-Glu11)

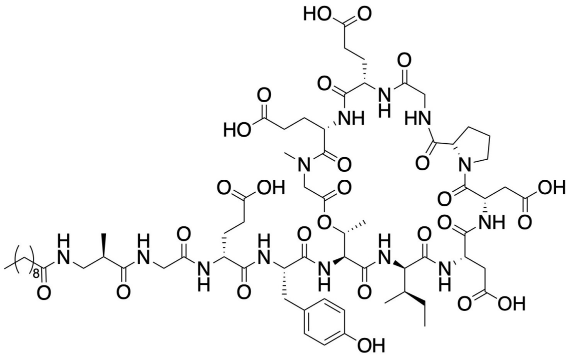

Table S19: Table of resin and amino acid quantities used per coupling step to yield cadaside analogue **C2**.

Amino acid	*M* [g/mol]	*m* [mg]	*n* [mmol]	*Equiv.*	*V* [mL]	*c* [M]
**Precursor CI2**		49.6	0.04	1	-	-
**Tripeptide 1**	668.76	77.4	0.12	3	0.5	0.12
**Decanoic acid**	172.26	21.3	0.12	3	0.5	0.12

To synthesise cadaside analogue **C2** from precursor **CI2**, we employed **General Procedure 11** using residues as displayed in **Table S19**.

The peptide was successfully synthesised, and three isolates were collected by prep-HPLC on a 5-95% B gradient over 27 minutes (A = 0.1% TFA in H_2O, B = 0.1% TFA in ACN). Isolate **C2a** eluted at 13.6 min, isolate **C2b** at 14.0 min and isolate **C2c** at 14.2 min. HRMS of all samples confirmed the correct *m/z* corresponding to the exact mass of the peptide. Analytical HPLC determined purity at 94% for **C2a** (24.912 min) and 97% for **C2b** (25.125 min). Due to the low yield, the mass was calculated through **Mass Calculation via Tyrosine Absorption at 280 nm**.

This yielded pure **C2a** (0.37 mg, 0.24 µmol, 0.60%), **C2b** (0.16 µg, 0.08 µmol, 0.20%) and **C2c** (0.15 µg, 0.08 µmol, 0.19%) as white solids.

SPPS of C3 (simplified cadaside, D-Glu11 → D-Asp11, L-Glu12 → L-Asp12)

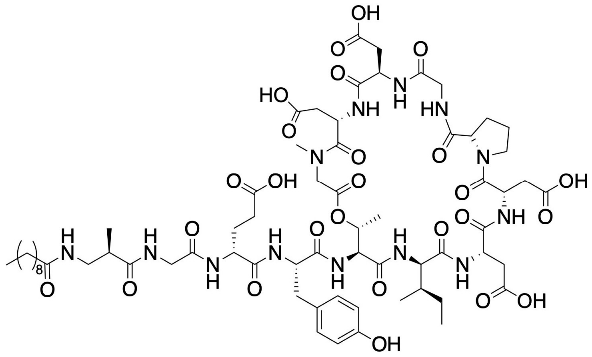

Table S20: Table of resin and amino acid quantities used per coupling step to yield cadaside analogue **C3**.

**Amino acid**	***M* [g/mol]**	***m* [mg]**	***n* [mmol]**	***Equiv.***	***V* [mL]**	***c* [M]**
**Precursor CI2**		101.2	0.08	1	-	-
**Tripeptide 3**	639.70	153.7	0.24	3	0.5	0.2
**Decanoic acid**	172.26	41.9	0.24	3	0.5	0.2

To synthesise cadaside analogue **C3** from precursor **CI2**, we employed **General Procedure 11** using residues as displayed in **Table S20**.

The peptide was successfully synthesised, and pure product was collected by prep-HPLC on a 5-95% B gradient over 27 minutes (A = 0.1% TFA in H_2O, B = 0.1% TFA in ACN), eluting at 14.2 min. HRMS of the sample confirmed the correct *m/z* corresponding to the exact mass of the peptide. Analytical HPLC determined purity at 100% for **C3** (24.776 min). Due to the low yield, the mass was calculated through **Mass Calculation via Tyrosine Absorption at 280 nm**.

This yielded pure **C3** (0.35 mg, 0.23 µmol, 0.31%) as a white solid.

SPPS of C4 (simplified cadaside, D-Glu11 → L-Asp11, L-Glu12 → L-Asp12)

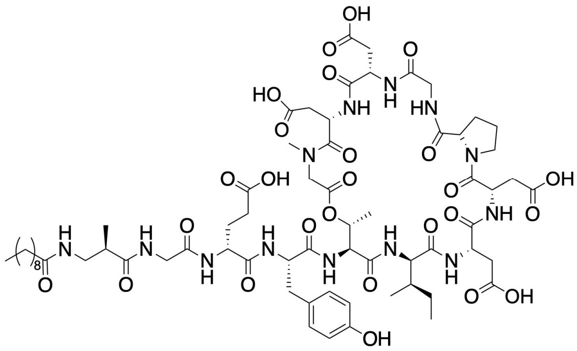

Table S21: Table of resin and amino acid quantities used per coupling step to yield cadaside analogue **C4**.

Amino acid	*M* [g/mol]	*m* [mg]	*n* [mmol]	*Equiv.*	*V* [mL]	*c* [M]
**Precursor CI2**		95.9	0.08	1	-	-
**Tripeptide 4**	639.70	154.1	0.24	3	0.5	0.2
**Decanoic acid**	172.26	41.7	0.24	3	0.5	0.2

To synthesise cadaside analogue **C4** from precursor **CI2**, we employed **General Procedure 11** using residues as displayed in **Table S21**.

The peptide was successfully synthesised, and pure product was collected by prep-HPLC on a 5-95% B gradient over 27 minutes (A = 0.1% TFA in H_2O, B = 0.1% TFA in ACN), eluting at 13.9 min. HRMS of the sample confirmed the correct *m/z* corresponding to the exact mass of the peptide. Analytical HPLC showed an inseparable shoulder for **C4** (24.776 min), making exact purity determination unreliable. No further impurities were detected. Due to the low yield, the mass was calculated through **Mass Calculation via Tyrosine Absorption at 280 nm**.

This yielded **C4** (0.42 mg, 0.28 µmol, 0.37%) as a white solid.

SPPS of C5 (simplified cadaside, D-Glu11 → D-Asp11)

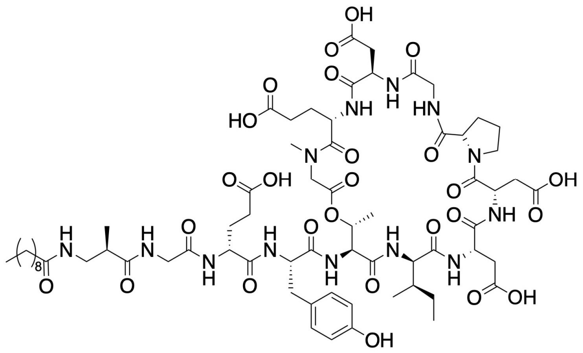

Table S22: Table of resin and amino acid quantities used per coupling step to yield cadaside analogue **C5**.

Amino acid	*M* [g/mol]	*m* [mg]	*n* [mmol]	*Equiv.*	*V* [mL]	*c* [M]
**Precursor CI2**		96.5	0.08	1	-	-
**Tripeptide 5**	653.73	157.3	0.24	3	0.5	0.2
**Decanoic acid**	172.26	42.0	0.24	3	0.5	0.2

To synthesise cadaside analogue **C5** from precursor **CI2**, we employed **General Procedure 11** using residues as displayed in **Table S22**.

The peptide was successfully synthesised, and pure product was collected by prep-HPLC on a 5-95% B gradient over 27 minutes (A = 0.1% TFA in H_2O, B = 0.1% TFA in ACN), eluting at 14.5 min. HRMS of the sample confirmed the correct *m/z* corresponding to the exact mass of the peptide. Analytical HPLC determined purity at 100% for **C5** (25.053 min). Due to the low yield, the mass was calculated through **Mass Calculation via Tyrosine Absorption at 280 nm**.

This yielded pure **C5** (0.34 mg, 0.23 µmol, 0.30%) as a white solid.

SPPS of C6 (simplified cadaside, D-Glu11 → L-Asp11)

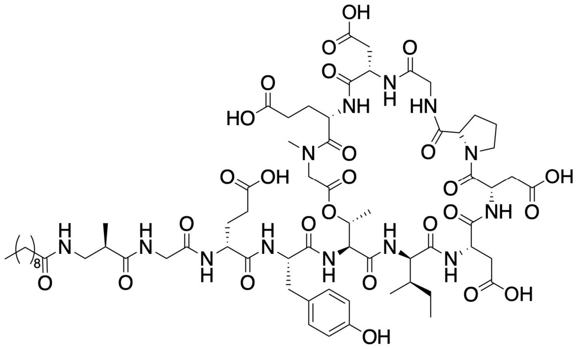

Table S23: Table of resin and amino acid quantities used per coupling step to yield cadaside analogue **C6**.

Amino acid	*M* [g/mol]	*m* [mg]	*n* [mmol]	*Equiv.*	*V* [mL]	*c* [M]
**Precursor CI2**		97.3	0.08	1	-	-
**Tripeptide 6**	653.73	157.1	0.24	3	0.5	0.2
**Decanoic acid**	172.26	41.9	0.24	3	0.5	0.2

To synthesise cadaside analogue **C6** from precursor **CI2**, we employed **General Procedure 11** using residues as displayed in **Table S23**.

The peptide was successfully synthesised, and pure product was collected by prep-HPLC on a 5-95% B gradient over 27 minutes (A = 0.1% TFA in H_2O, B = 0.1% TFA in ACN), eluting at 14.5 min. HRMS of the sample confirmed the correct *m/z* corresponding to the exact mass of the peptide. Analytical HPLC determined purity at 100% for **C6** (24.461 min). Due to the low yield, the mass was calculated through **Mass Calculation via Tyrosine Absorption at 280 nm**.

This yielded pure **C6** (0.21 mg, 0.14 µmol, 0.18%) as a white solid.

SPPS of C7 (simplified cadaside, L-Glu12 → L-Asp12)

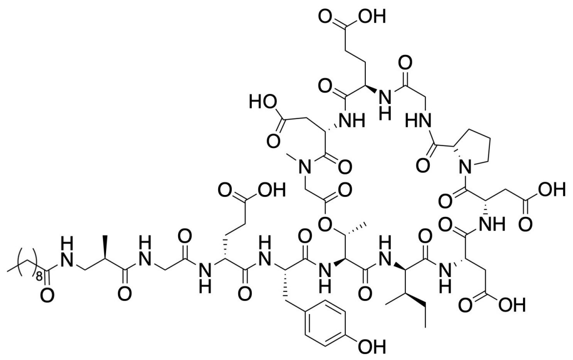

Table S24: Table of resin and amino acid quantities used per coupling step to yield cadaside analogue **C7**.

Amino acid	*M* [g/mol]	*m* [mg]	*n* [mmol]	*Equiv.*	*V* [mL]	*c* [M]
**Precursor Cl2**		94.5	0.08	1	-	-
**Tripeptide 7**	653.73	157.2	0.24	3	0.5	0.2
**Decanoic acid**	172.26	41.6	0.24	3	0.5	0.2

To synthesise cadaside analogue **C7** from precursor **Cl2**, we employed **General Procedure 11** using residues as displayed in **Table S24**.

The peptide was successfully synthesised, and two isolates were collected by prep-HPLC on a 5-95% B gradient over 27 minutes (A = 0.1% TFA in H_2O, B = 0.1% TFA in ACN). Isolate **C7a** eluted at 14.2 min and isolate **C7b** at 14.6 min. HRMS of both samples confirmed the correct *m/z* corresponding to the exact mass of the peptide. Analytical HPLC determined purity at 100% for **C7a** (24.711 min) and 100% for **C7b** (24.892 min). Due to the low yield, the mass was calculated through **Mass Calculation via Tyrosine Absorption at 280 nm**.

This yielded pure **C7a** (0.38 mg, 0.25 µmol, 0.33%) and **C7b** (0.15 µg, 0.10 µmol, 0.13%) as white solids.

SPPS of C8 (simplified cadaside, D-Glu11 → L-Glu11, L-Glu12 → L-Asp12)

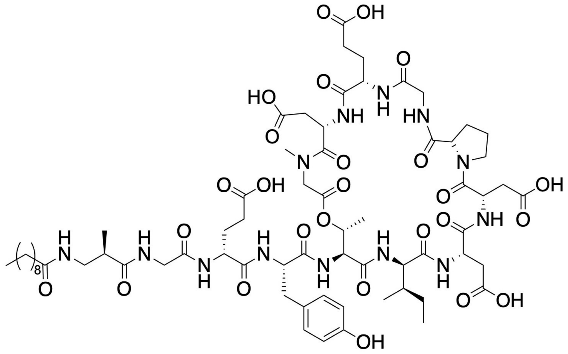

Table S25: Table of resin and amino acid quantities used per coupling step to yield cadaside analogue **C8**.

Amino acid	*M* [g/mol]	*m* [mg]	*n* [mmol]	*Equiv.*	*V* [mL]	*c* [M]
**Precursor Cl2**		107.9	0.09	1	-	-
**Tripeptide 8**	653.73	176.7	0.24	3	0.5	0.2
**Decanoic acid**	172.26	41.8	0.24	3	0.5	0.2

To synthesise cadaside analogue **C8** from precursor **Cl2**, we employed **General Procedure 11** using residues as displayed in **Table S25**.

The peptide was successfully synthesised, and pure product was collected by prep-HPLC on a 5-95% B gradient over 27 minutes (A = 0.1% TFA in H_2O, B = 0.1% TFA in ACN), eluting at 13.8 min. HRMS of the sample confirmed the correct *m/z* corresponding to the exact mass of the peptide. Analytical HPLC determined purity at 100% for **C8** (24.893 min). Due to the low yield, the mass was calculated through **Mass Calculation via Tyrosine Absorption at 280 nm**.

This yielded pure **C8** (0.24 mg, 0.16 µmol, 0.21%) as a white solid.

SPPS of C9 (simplified cadaside, Gly insertion pos12)

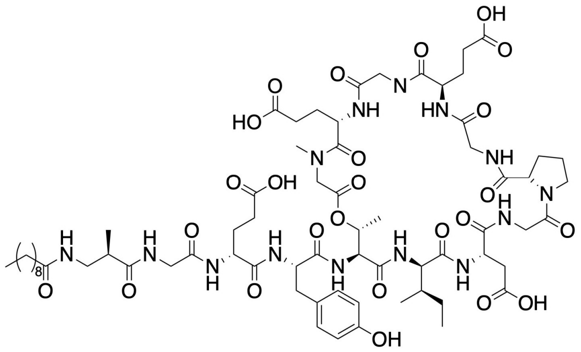

Table S26: Table of resin and amino acid quantities used per coupling step to yield cadaside analogue **C9**.

Amino acid	*M* [g/mol]	*m* [mg]	*n* [mmol]	*Equiv.*	*V* [mL]	*c* [M]
**Precursor Cl2**		50.2	0.04	1	-	-
**Tetrapeptide 9**	724.81	89.7	0.12	3	0.5	0.12
**Decanoic acid**	172.26	21.8	0.12	3	0.5	0.12

To synthesise cadaside analogue **C9** from precursor **Cl2**, we employed **General Procedure 11** using residues as displayed in **Table S26**.

The peptide was successfully synthesised, and pure product was collected by prep-HPLC on a 5-95% B gradient over 27 minutes (A = 0.1% TFA in H_2O, B = 0.1% TFA in ACN), eluting at 17.5 min. HRMS of the sample confirmed the correct *m/z* corresponding to the exact mass of the peptide. Analytical HPLC determined purity at 100% for **C9** (24.840 min). Due to the low yield, the mass was calculated through **Mass Calculation via Tyrosine Absorption at 280 nm**.

This yielded pure **C9** (0.21 mg, 0.13 µmol, 0.33%) as a white solid.

SPPS of C10 (simplified cadaside, Gly insertion pos12, D-Glu11 → L-Asp11, L-Glu13 → L-Asp13)

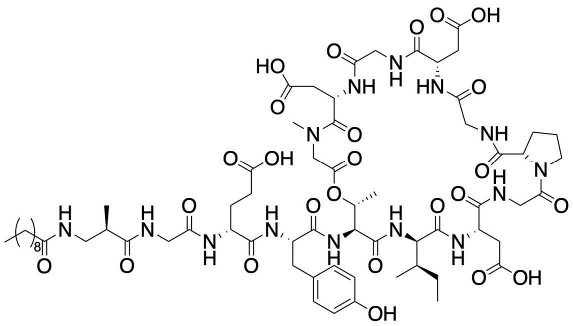

Table S27: Table of resin and amino acid quantities used per coupling step to yield cadaside analogue **C10**.

Amino acid	*M* [g/mol]	*m* [mg]	*n* [mmol]	*Equiv.*	*V* [mL]	*c* [M]
**Precursor CI2**		51.1	0.04	1	-	-
**Tetrapeptide 10**	695.75	85.1	0.12	3	0.5	0.12
**Decanoic acid**	172.26	22.3	0.12	3	0.5	0.12

To synthesise cadaside analogue **C10** from precursor **CI2**, we employed **General Procedure 11** using residues as displayed in **Table S27**.

The peptide was successfully synthesised, and pure product was collected by prep-HPLC on a 5-95% B gradient over 27 minutes (A = 0.1% TFA in H_2O, B = 0.1% TFA in ACN), eluting at 16.3 min. Analytical HPLC showed an inseparable shoulder for **C10** (24.546 min), making exact purity determination unreliable. No further impurities were detected. The mass of obtained peptide was determined by gravimetric weighing.

This yielded **C10** (0.2 mg, 0.13 µmol, 0.32%) as a white solid.

5. Total Synthesis of Malacidin analogues M1-M5

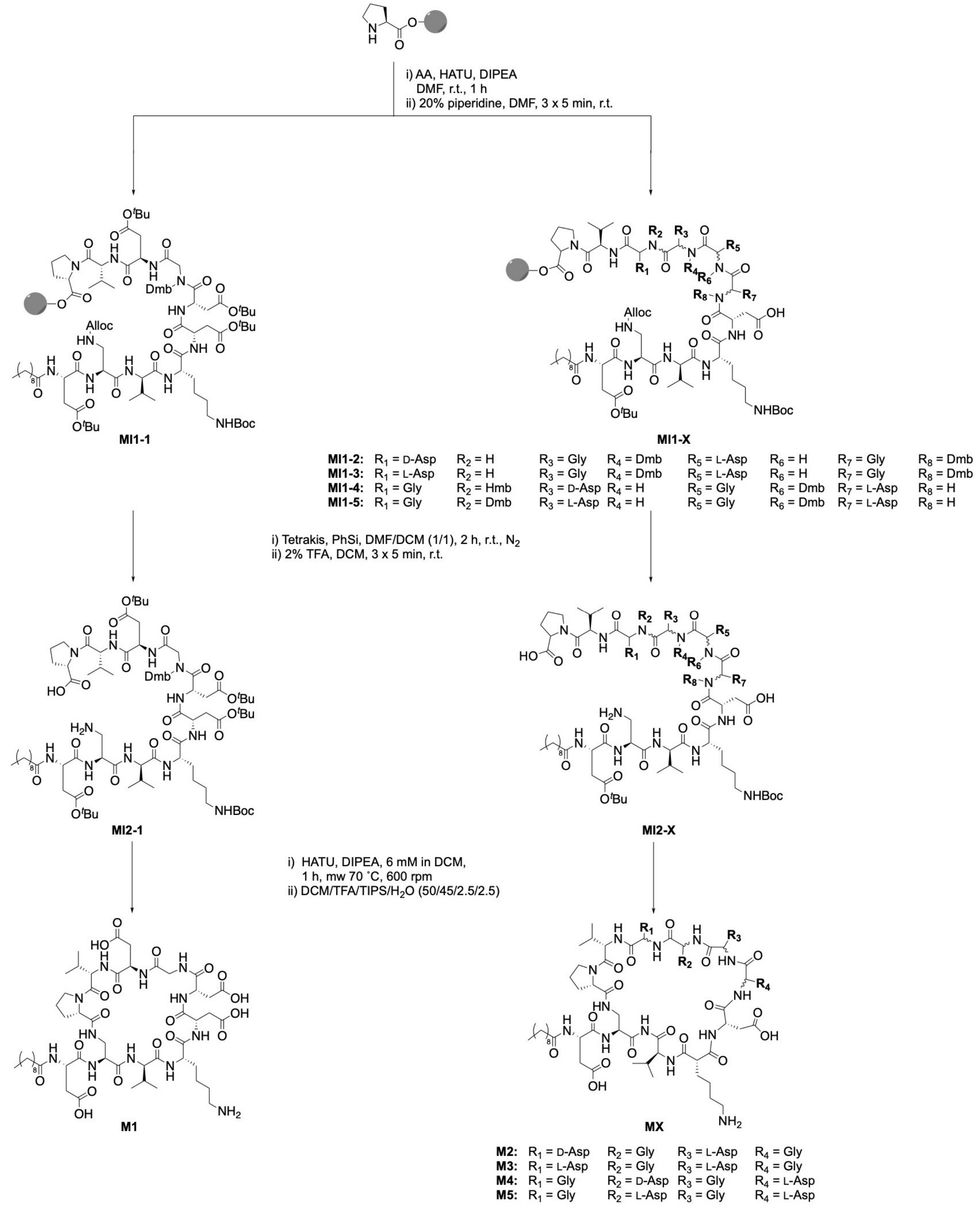

Scheme S3: Total synthesis of malacidin analogues.

M1 (simplified malacidin)

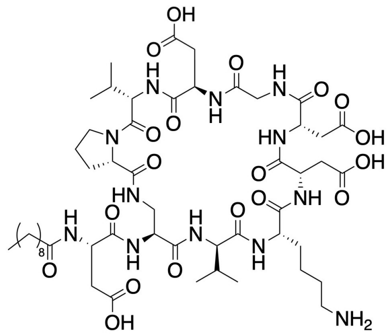

Table S28: Table of resin and amino acid quantities used per coupling step to yield linear precursor **MI1-1**.

**Amino acid**	***M* [g/mol]**	***m* [mg]**	***n* [mmol]**	***V* [mL]**	***c* [M]**
**H-L-Pro-OCTC**	0.82 mmol/g	209.5	0.17	-	-
**Fmoc-D-Val-OH**	339.39	179.3	0.53	2.4	0.22
**Fmoc-D-Asp(OtBu)-OH**	411.45	219.9	0.53	2.4	0.22
**Fmoc-L-Asp(OtBu)-(Dmb)Gly-OH**	618.67	326.0	0.53	2.4	0.22
**Fmoc-L-Asp(OtBu)-OH**	411.45	221.1	0.54	2.4	0.22
**Fmoc-L-Lys(Boc)-OH**	468.54	248.0	0.53	2.4	0.22
**Fmoc-L-Val-OH**	339.39	181.0	0.53	2.4	0.22
**Fmoc-L-Dap(Alloc)-OH**	410.42	200.0	0.49	2.4	0.22
**Fmoc-L-Asp(OtBu)-OH**	411.45	224.6	0.55	2.4	0.23
**Decanoic acid**	172.26	215.5	1.26	4.8	0.26

M1 was synthesised using **General procedure 12** using residues displayed in **Table S28**.

The peptide was successfully synthesised, and pure product was collected by prep-HPLC on a 5-95% B gradient over 28 minutes (A = 0.1% TFA in H_2O, B = 0.1% TFA in ACN), eluting at 14.8 min. HRMS of the sample confirmed the correct *m/z* corresponding to the exact mass of the peptide. Analytical HPLC determined purity at 96% for **M1** (24.408 min).

The final peptide was yielded as a white powder (2.8 mg, 2.2 µmol, 1.3%).

M2 (simplified malacidin, Gly6 insertion)

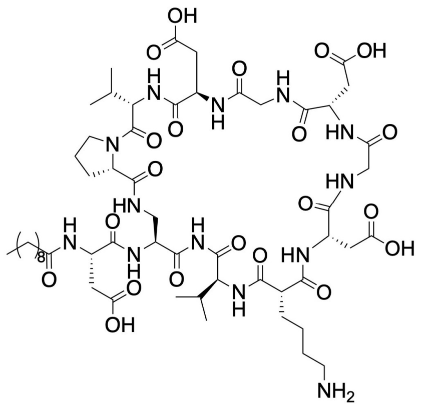

Table S29: Table of resin and amino acid quantities used per coupling step to yield linear precursor **Ml1-2**.

**Amino acid**	***M* [g/mol]**	***m* [mg]**	***n* [nmol]**	***V* [mL]**	***c* [M]**
**H-L-Pro-OCTC**	0.82 mmol/g	92.1	75.4	-	-
**Fmoc-D-Val-OH**	339.39	77.1	0.23	1	0.22
**Fmoc-D-Asp(OtBu)-OH**	411.45	94.1	0.23	1	0.22
**Fmoc-L-Asp(OtBu)-(Dmb)Gly-OH**	618.67	140.3	0.23	1	0.22
**Fmoc-L-Lys(Boc)-OH**	468.54	106.8	0.23	1	0.22
**Fmoc-L-Val-OH**	339.39	77.2	0.23	1	0.22
**Fmoc-L-Dap(Alloc)-OH**	410.42	93.1	0.23	1	0.22
**Fmoc-L-Asp(OtBu)-OH**	411.45	93.4	0.23	1	0.22
**Decanoic acid**	172.26	79.1	0.46	2	0.22

M2 was synthesised using **General procedure 12** using residues displayed in **Table S29**.

The peptide was successfully synthesised, and pure product was collected by prep-HPLC on a 5-95% B gradient over 28 minutes (A = 0.1% TFA in H_2O, B = 0.1% TFA in ACN), eluting at 14.8 min. HRMS of the sample confirmed the correct *m/z* corresponding to the exact mass of the peptide. Analytical HPLC determined purity at 99% for **M2** (24.094 min).

The final peptide was yielded as a white powder (3.8 mg, 2.8 μmol, 3.7%).

M3 (simplified malacidin, Gly6 insertion, D-Asp9 → L-Asp9)

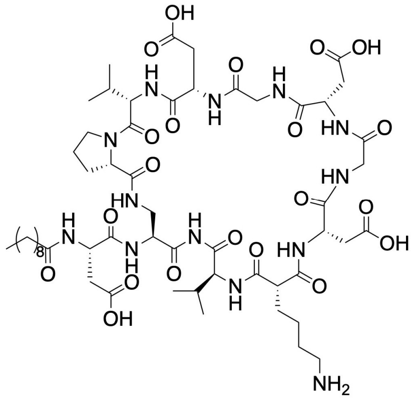

Table S30: Table of resin and amino acid quantities used per coupling step to yield linear precursor **MI1-3**.

Amino acid	*M* [g/mol]	*m* [mg]	*n* [nmol]	*V* [mL]	*c* [M]
**H-L-Pro-OCTC**	0.82 mmol/g	91.9	75.3	-	-
**Fmoc-D-Val-OH**	339.39	77.0	0.23	1	0.22
**Fmoc-L-Asp(OtBu)-OH**	411.45	94.2	0.23	1	0.22
**Fmoc-L-Asp(OtBu)-(Dmb)Gly-OH**	618.67	140.1	0.23	1	0.22
**Fmoc-L-Lys(Boc)-OH**	468.54	106.4	0.23	1	0.22
**Fmoc-L-Val-OH**	339.39	77.1	0.23	1	0.22
**Fmoc-L-Dap(Alloc)-OH**	410.42	92.7	0.23	1	0.22
**Decanoic acid**	172.26	78.8	0.46	2	0.22

M3 was synthesised using **General procedure 12** using residues displayed in **Table S30**.

The peptide was successfully synthesised, and pure product was collected by prep-HPLC on a 5-95% B gradient over 28 minutes (A = 0.1% TFA in H_2O, B = 0.1% TFA in ACN), eluting at 14.8 min. HRMS of the sample confirmed the correct *m/z* corresponding to the exact mass of the peptide. Analytical HPLC determined purity at 91% for **M3** (23.681 min).

The final peptide was yielded as a white powder (1.9 mg, 1.4 µmol, 1.9%).

M4 (simplified malacidin, Gly9 insertion)

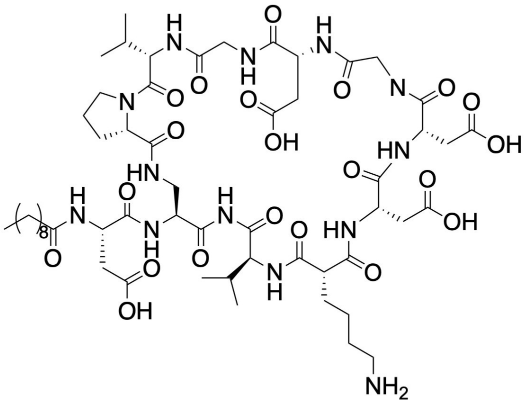

Table S31: Table of resin and amino acid quantities used per coupling step to yield linear precursor **MI1-4**.

**Amino acid**	***M*** **[g/mol]**	***m*** **[mg]**	***n*** **[nmol]**	***V*** **[mL]**	***c*** **[M]**
**H-L-Pro-OCTC**	0.82 mmol/g	90.8	74.5	-	-
**Fmoc-D-Val-OH**	339.39	76.1	0.22	1	0.22
**Fmoc-L-Asp(OtBu)-(Dmb)Gly-OH**	618.67	138.8	0.22	1	0.22
**Fmoc-D-Asp(OtBu)-(Hmb)Gly-OH**	604.67	135.6	0.22	1	0.22
**Fmoc-L-Asp(OtBu)-OH**	411.45	92.2	0.22	1	0.22
**Fmoc-L-Lys(Boc)-OH**	468.54	104.9	0.22	1	0.22
**Fmoc-L-Val-OH**	339.39	76.2	0.22	1	0.22
**Fmoc-L-Dap(Alloc)-OH**	410.42	92.1	0.22	1	0.22
**Decanoic acid**	172.26	77.4	0.44	2	0.22

M4 was synthesised using **General procedure 12** using residues displayed in **Table S31**.

The peptide was successfully synthesised, and pure product was collected by prep-HPLC on a 5-95% B gradient over 28 minutes (A = 0.1% TFA in H_2O, B = 0.1% TFA in ACN), eluting at 14.8 min. HRMS of the sample confirmed the correct *m/z* corresponding to the exact mass of the peptide. Analytical HPLC determined purity at 100% for **M4** (23.923 min).

The final peptide was yielded as a white powder (3.5 mg, 2.6 µmol, 3.5%).

M5 (simplified malacidin, Gly9 insertion, D-Asp8 → L-Asp8)

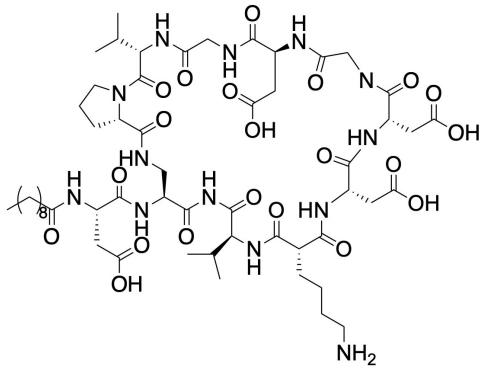

Table S32: Table of resin and amino acid quantities used per coupling step to yield linear precursor **MI1-5**.

Amino acid	*M* [g/mol]	*m* [mg]	*n* [nmol]	*V* [mL]	*c* [M]
**H-L-Pro-OCTC**	0.82 mmol/g	91.1	74.7	-	-
**Fmoc-D-Val-OH**	339.39	76.2	0.22	1	0.22
**Fmoc-L-Asp(OtBu)-(Dmb)Gly-OH**	618.67	138.9	0.22	1	0.22
**Fmoc-L-Asp(OtBu)-OH**	411.45	92.5	0.22	1	0.22
**Fmoc-L-Lys(Boc)-OH**	468.54	105.6	0.22	1	0.22
**Fmoc-L-Val-OH**	339.39	76.3	0.22	1	0.22
**Fmoc-L-Dap(Alloc)-OH**	410.42	92.3	0.22	1	0.22
**Decanoic acid**	172.26	77.3	0.44	2	0.22

M5 was synthesised using **General procedure 12** using residues displayed in **Table S32**.

The peptide was successfully synthesised, and pure product was collected by prep-HPLC on a 5-95% B gradient over 28 minutes (A = 0.1% TFA in H_2O, B = 0.1% TFA in ACN), eluting at 14.8 min. HRMS of the sample confirmed the correct *m/z* corresponding to the exact mass of the peptide. Analytical HPLC determined purity at 86% for **M5** (24.127 min).

The final peptide was yielded as a white powder (1.6 mg, 1.2 μmol, 1.6%).

6. Analytical Data

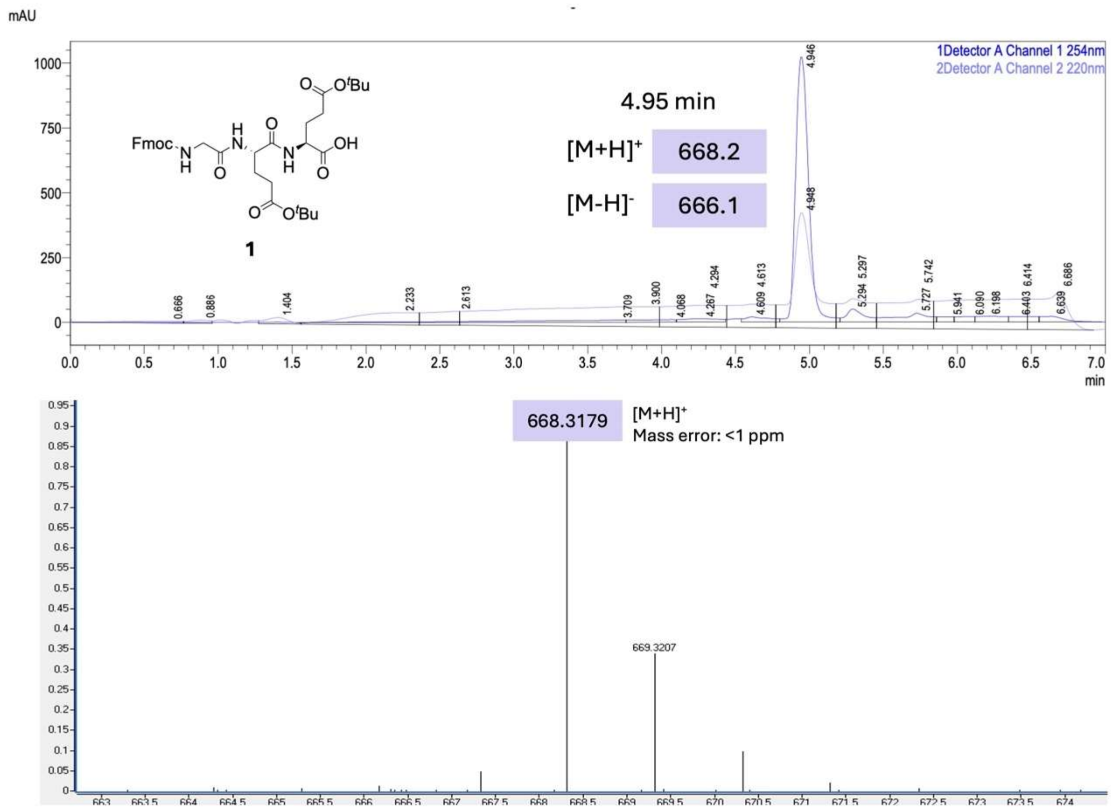

Figure S10: LC-MS chromatogram of crude tripeptide **1** (top); HRMS spectrum of crude tripeptide **1** (bottom), confirming the *m/z* corresponding to the exact mass of 667.3105; Expected: 668.3178 *m/z*, found: 668.3179 *m/z*, mass error <1 ppm.

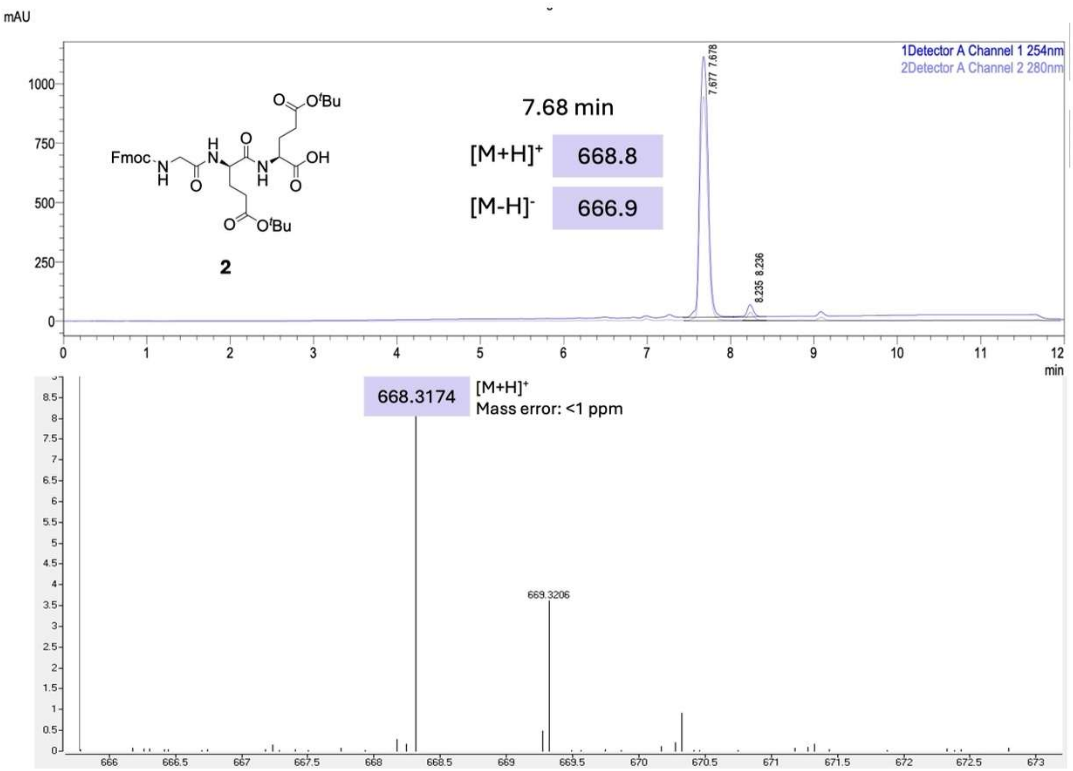

Figure S11: LC-MS chromatogram of crude tripeptide **2** (top); HRMS spectrum of crude tripeptide **2** (bottom), confirming the m/z corresponding to the exact mass of 667.3105; Expected: 668.3178 m/z, found: 668.3174 m/z, mass error <1 ppm.

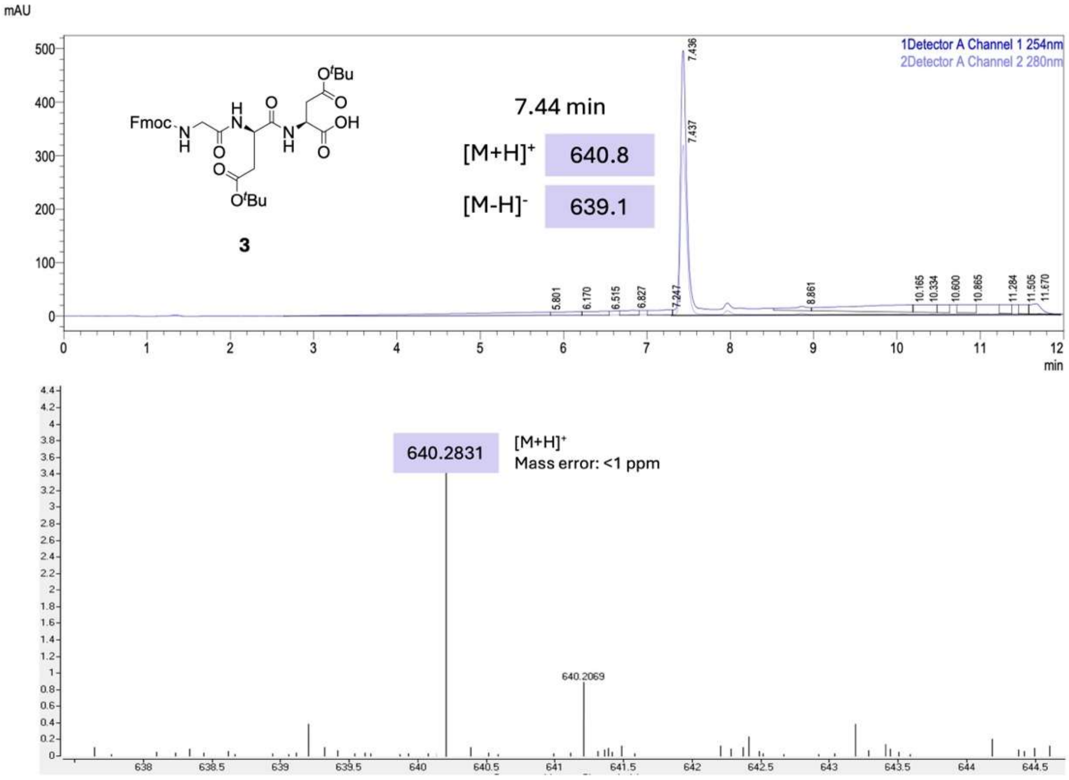

Figure S12: LC-MS chromatogram of crude tripeptide **3** (top); HRMS spectrum of crude tripeptide **3** (bottom), confirming the *m/z* corresponding to the exact mass of 639.2792; Expected: 640.2865 *m/z*, found: 640.2831 *m/z*, mass error <1 ppm.

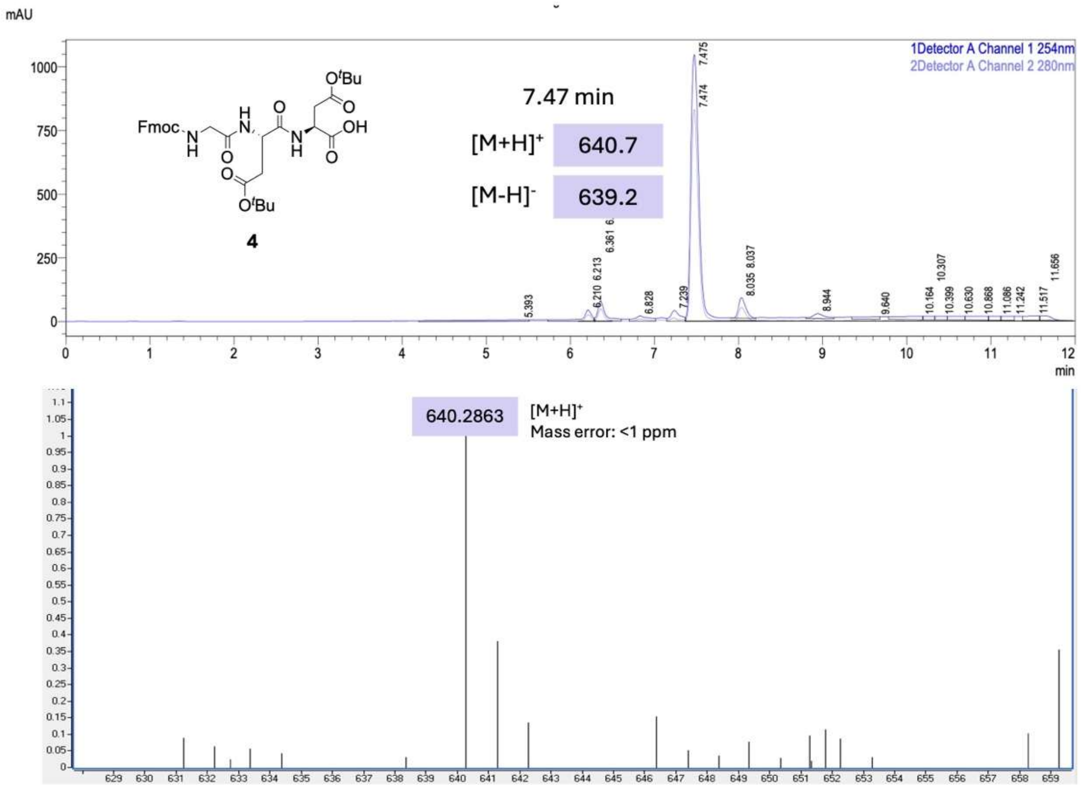

Figure S13: LC-MS chromatogram of crude tripeptide **4** (top); HRMS spectrum of crude tripeptide **4** (bottom), confirming the *m/z* corresponding to the exact mass of 639.2792; Expected: 640.2865 *m/z*, found: 640.2863 *m/z*, mass error <1 ppm.

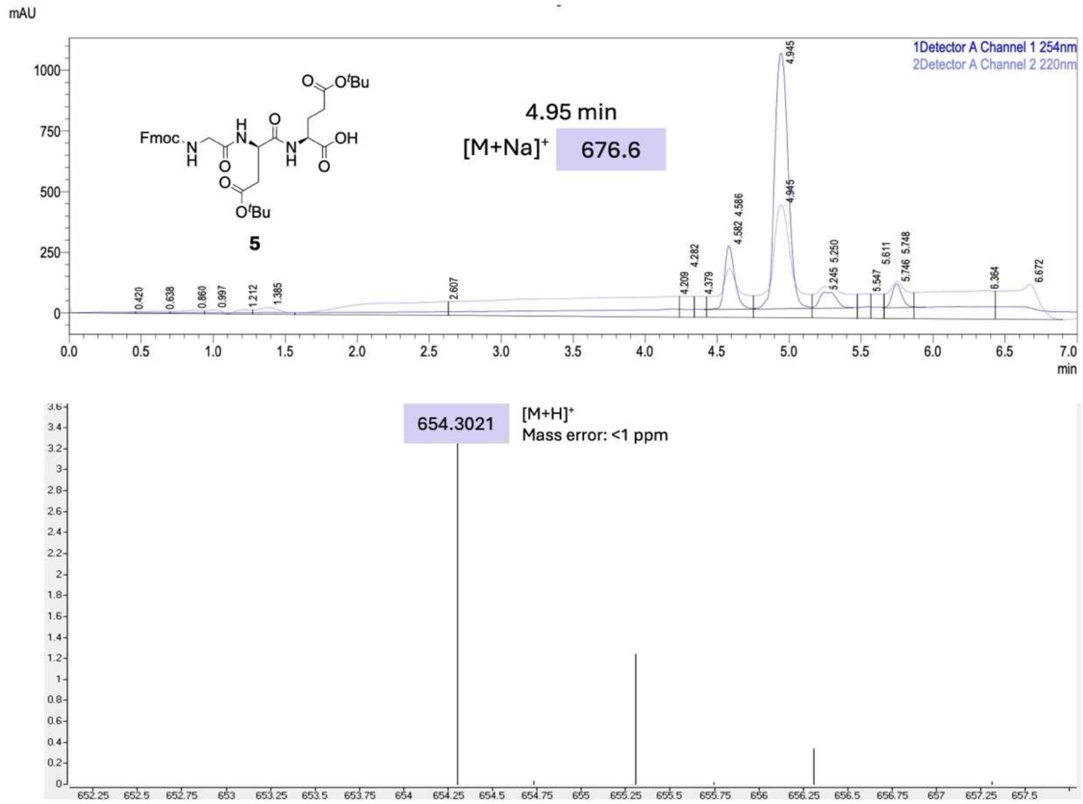

Figure S14: LC-MS chromatogram of crude tripeptide **5** (top); HRMS spectrum of crude tripeptide **5** (bottom), confirming the *m/z* corresponding to the exact mass of 653.2948; Expected: 654.3021 *m/z*, found: 654.3021 *m/z*, mass error <1 ppm.

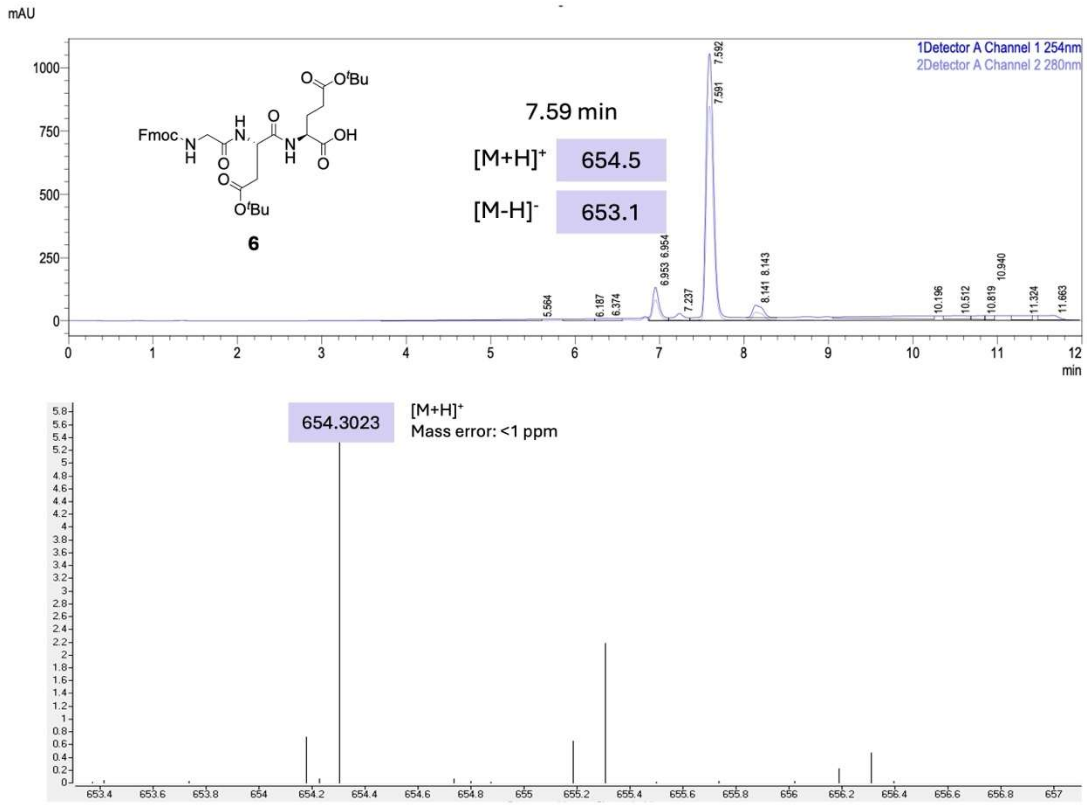

Figure S15: LC-MS chromatogram of crude tripeptide **6** (top); HRMS spectrum of crude tripeptide **6** (bottom), confirming the *m/z* corresponding to the exact mass of 653.2948; Expected: 654.3021 *m/z*, found: 654.3023 *m/z*, mass error <1 ppm.

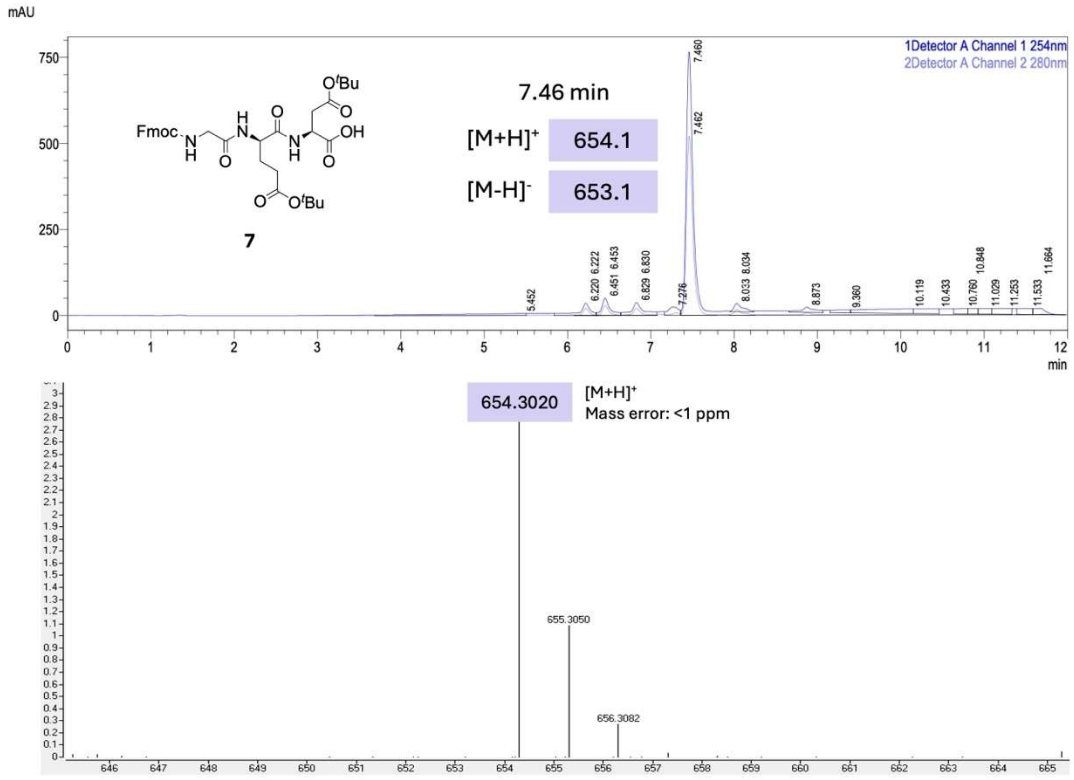

Figure S16: LC-MS chromatogram of crude tripeptide **7** (top); HRMS spectrum of crude tripeptide **7** (bottom), confirming the *m/z* corresponding to the exact mass of 653.2948; Expected: 654.3021 *m/z*, found: 654.3020 *m/z*, mass error <1 ppm.

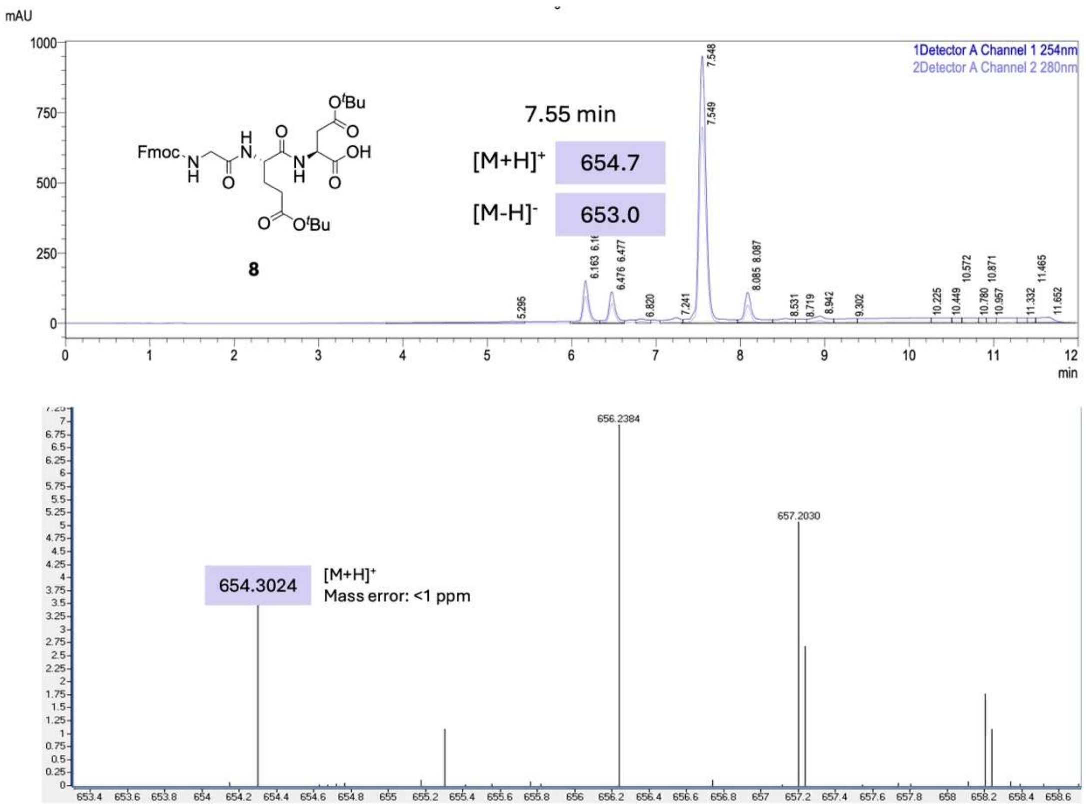

Figure S17: LC-MS chromatogram of crude tripeptide **8** (top); HRMS spectrum of crude tripeptide **8** (bottom), confirming the *m/z* corresponding to the exact mass of 653.2948; Expected: 654.3021 *m/z*, found: 654.3024 *m/z*, mass error <1 ppm.

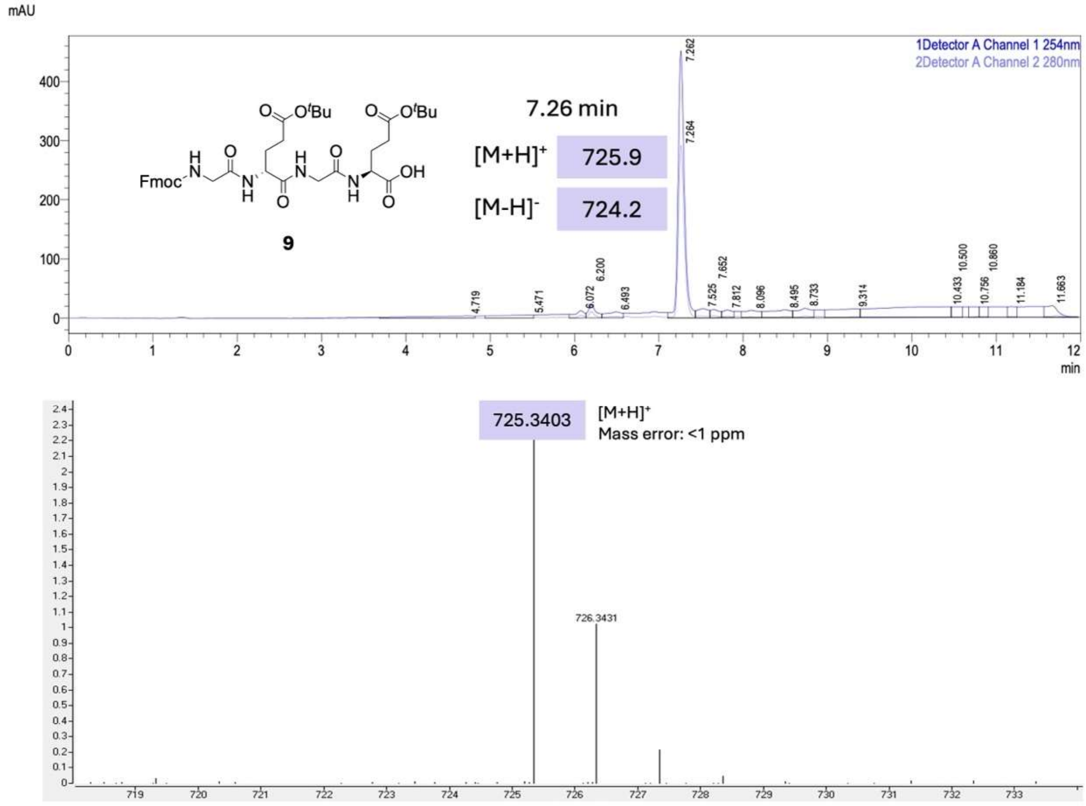

Figure S18: LC-MS chromatogram of crude tetrapeptide **9** (top); HRMS spectrum of crude tetrapeptide **9** (bottom), confirming the *m/z* corresponding to the exact mass of 724.3320; Expected: 725.3393 *m/z*, found: 725.3403 *m/z*, mass error <1 ppm.

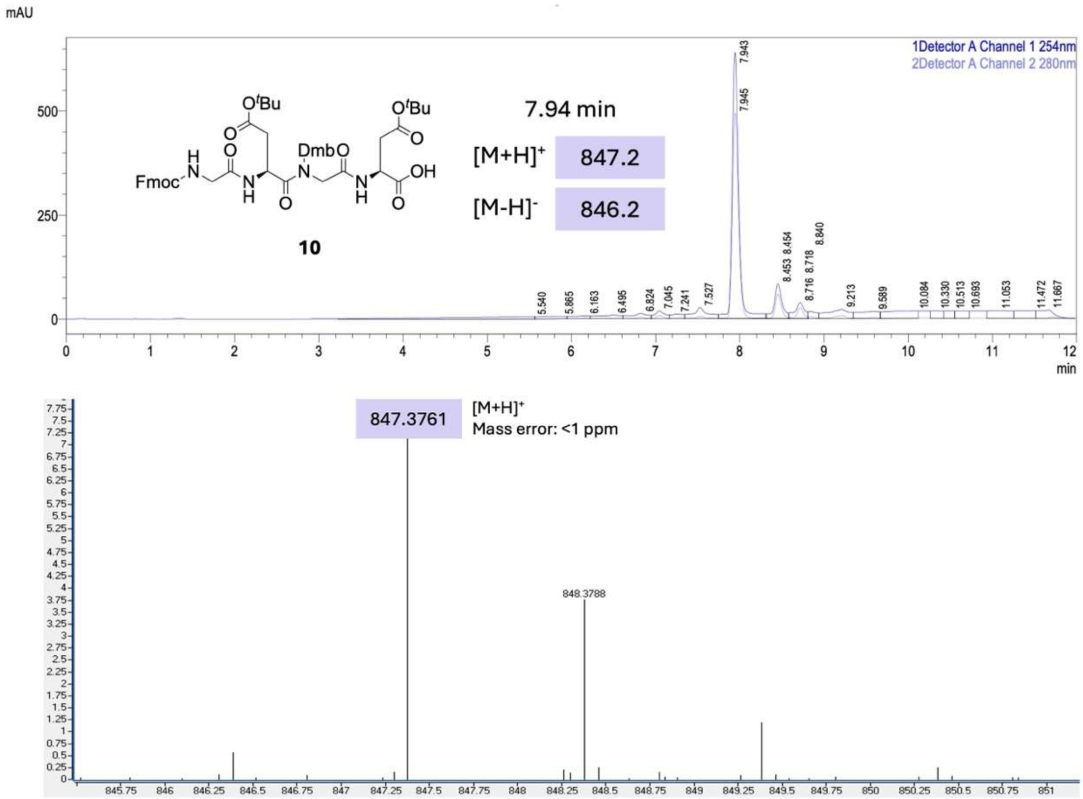

Figure S19: LC-MS chromatogram of crude tetrapeptide **10** (top); HRMS spectrum of crude tetrapeptide **10** (bottom), confirming the m/z corresponding to the exact mass of 846.3687; Expected: 847.3760 m/z, found: 847.3761 m/z, mass error <1 ppm.

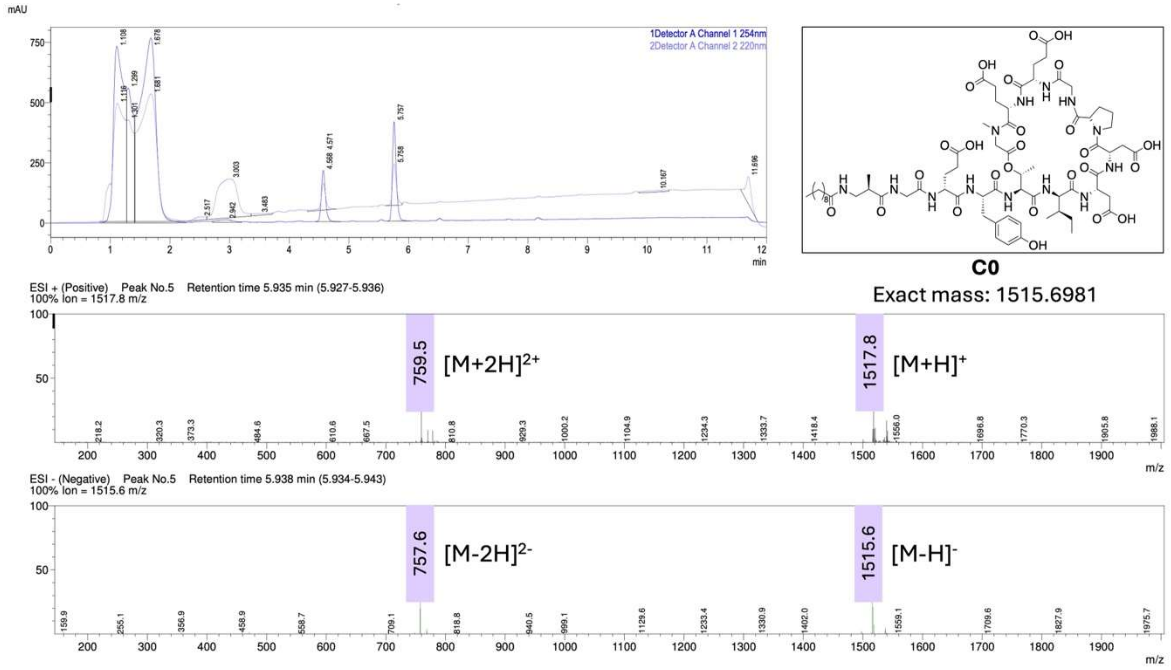

Figure S20: LC-MS spectrum of **C0**, confirming the *m/z* corresponding to the exact mass of 1515.6981; Expected: $[M+H]^+$ 1516.7 *m/z*, $[M+H]^{2+}$ 758.9, $[M-H]^-$ 1514.7 *m/z*, $[M-H]^{2-}$ 756.8 *m/z*, found: $[M+H]^+$ 1517.8 *m/z*, $[M+H]^{2+}$ 759.5, $[M-H]^-$ 1515.6 *m/z*, $[M-H]^{2-}$ 757.6 *m/z*.

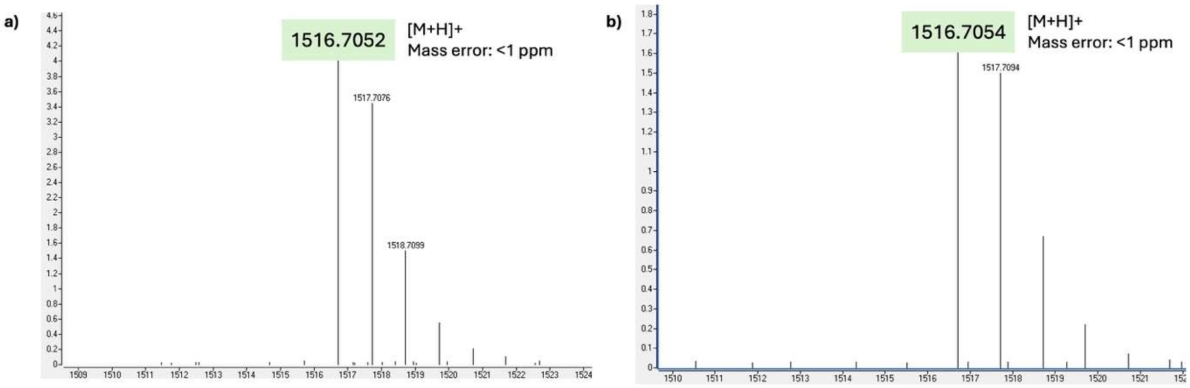

Figure S21: HRMS data for **C1**. **a)** HRMS spectrum of pure **C1a**, confirming the *m/z* corresponding to the exact mass of 1515.6981; Expected: 1516.7052 *m/z*, found: 1516.7052 *m/z*, mass error <1 ppm. **b)** HRMS spectrum of pure **C1b**, confirming the *m/z* corresponding to the exact mass of 1515.6981; Expected: 1516.7052 *m/z*, found: 1516.7054 *m/z*, mass error <1 ppm.

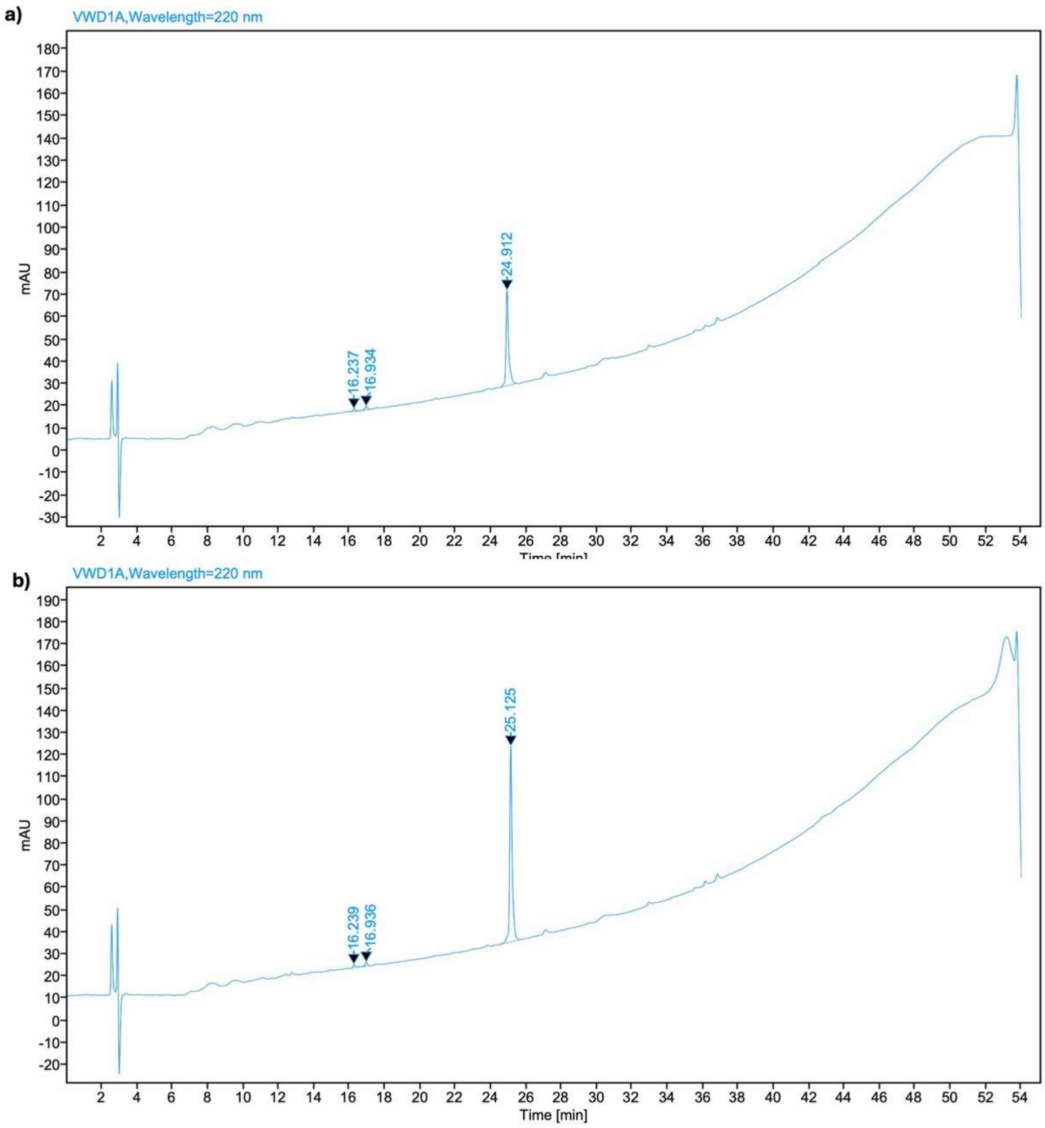

Figure S22: Analytical HPLC chromatograms of pure **a) C1a** (retention time: 24.912 min: purity: 94%); **b) C1b** (retention time: 25.125 min: purity: 97%).

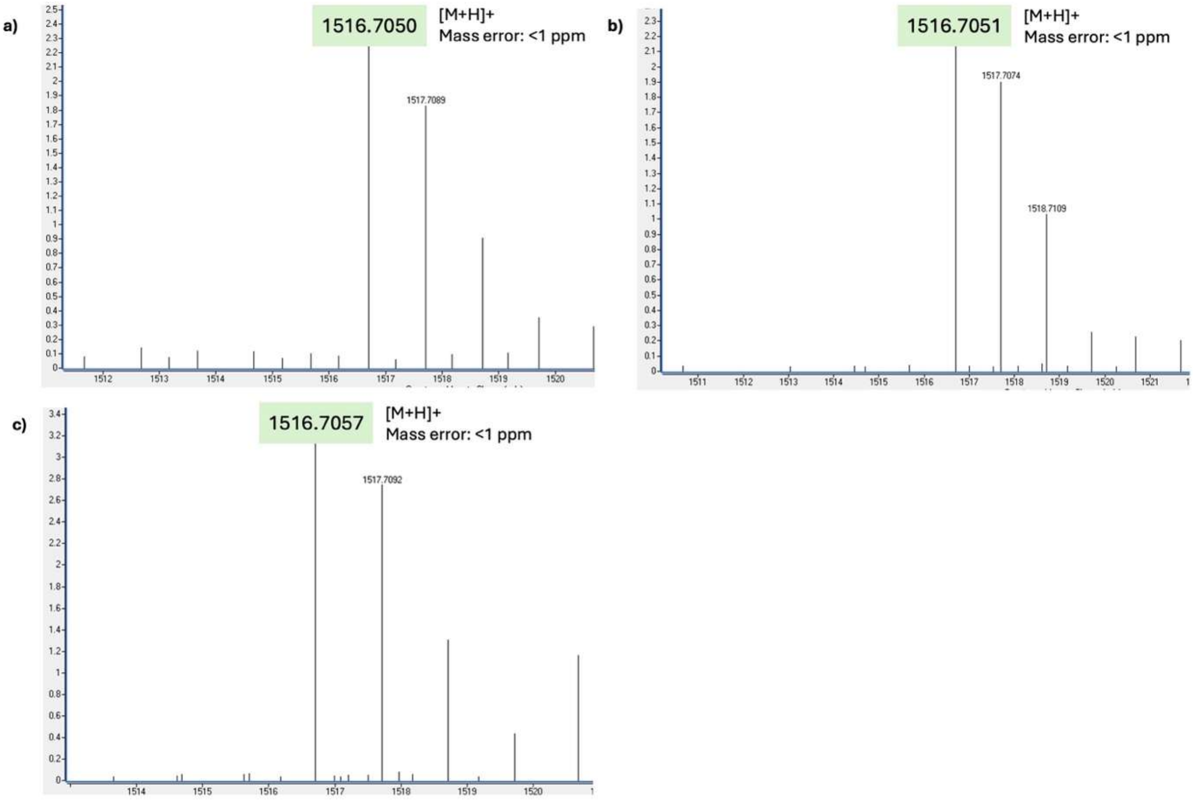

Figure S23: HRMS data for **C2**. **a)** HRMS spectrum of pure **C2a**, confirming the *m/z* corresponding to the exact mass of 1515.6981; Expected: 1516.7052 *m/z*, found: 1516.7050 *m/z*, mass error <1 ppm. **b)** HRMS spectrum of pure **C2b**, confirming the *m/z* corresponding to the exact mass of 1515.6981; Expected: 1516.7052 *m/z*, found: 1516.7051 *m/z*, mass error <1 ppm. **c)** HRMS spectrum of pure **C2c**, confirming the *m/z* corresponding to the exact mass of 1515.6981; Expected: 1516.7052 *m/z*, found: 1516.7057 *m/z*, mass error <1 ppm.

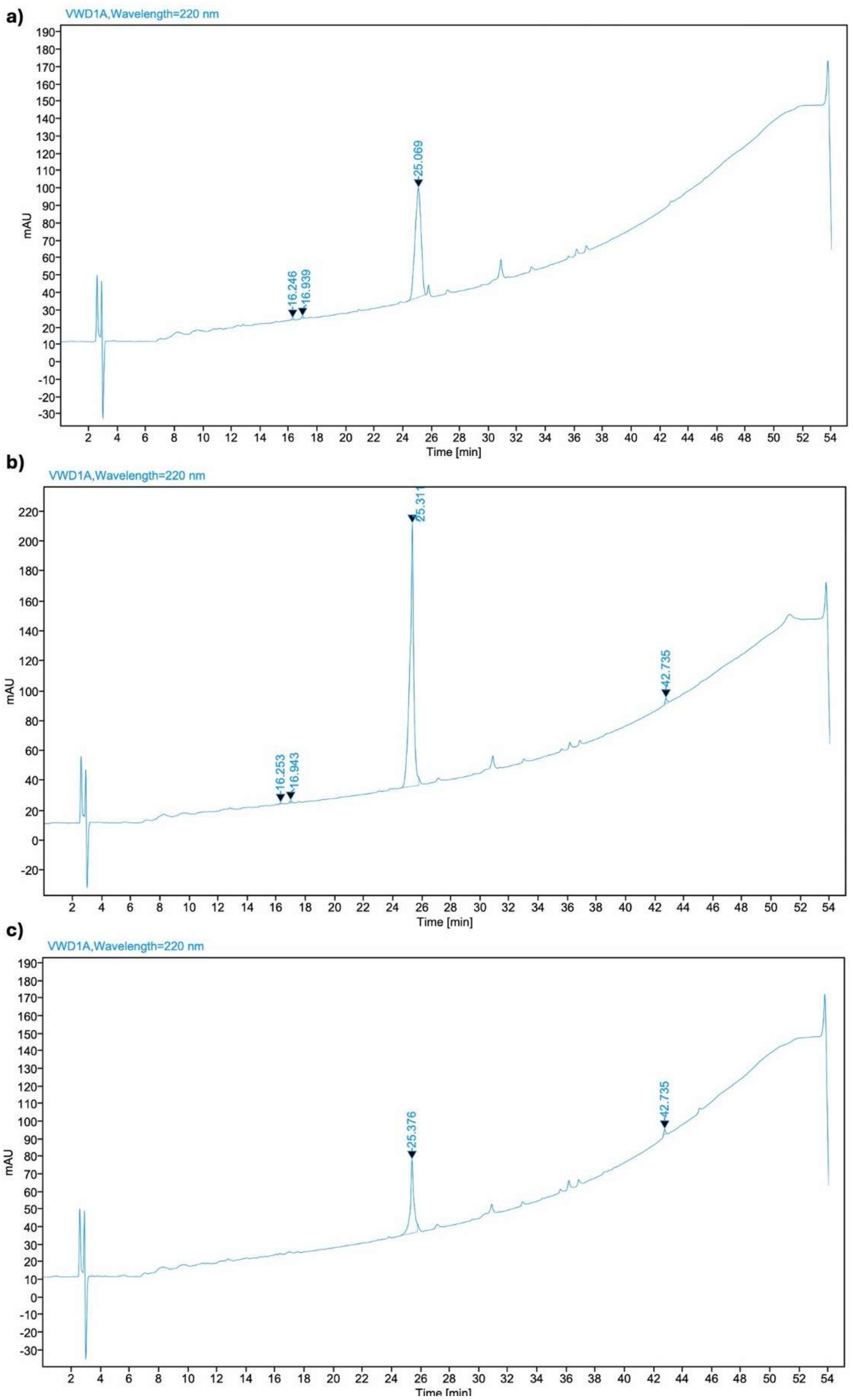

Figure S24: Analytical HPLC chromatograms of pure **a) C2a** (retention time: 25.069 min: purity: 99%); **b) C2b** (retention time: 25.311 min: purity: 98%); and **c) C2c** (retention time: 25.376 min: purity: 97%).

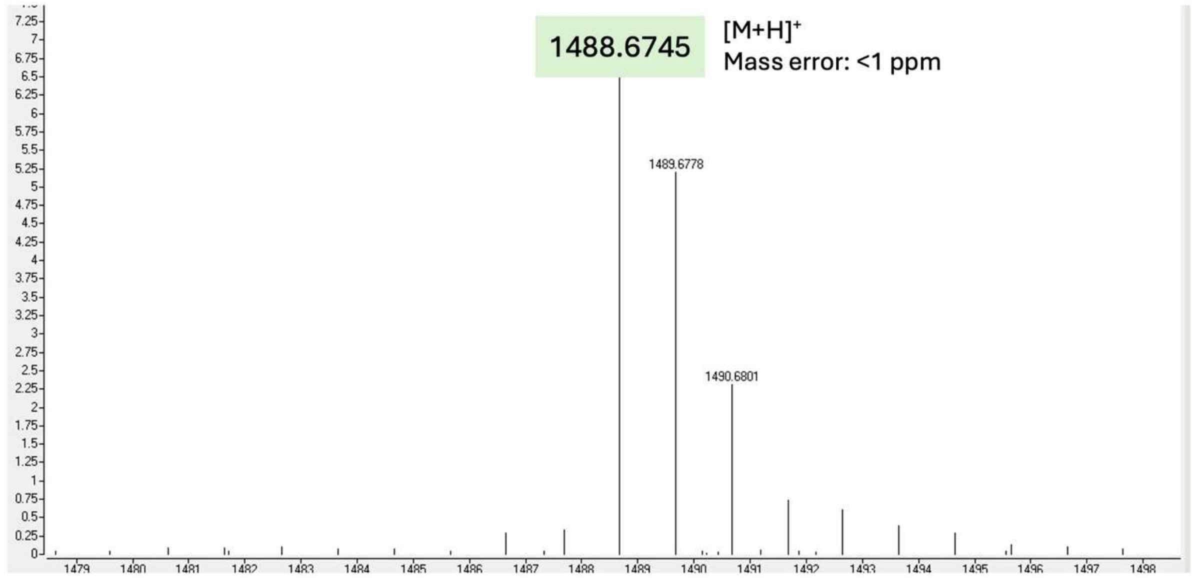

Figure S25: HRMS spectrum of pure **C3**, confirming the *m/z* corresponding to the exact mass of 1487.6668; Expected: 1488.6741 *m/z*, found: 1488.6745 *m/z*, mass error <1 ppm.

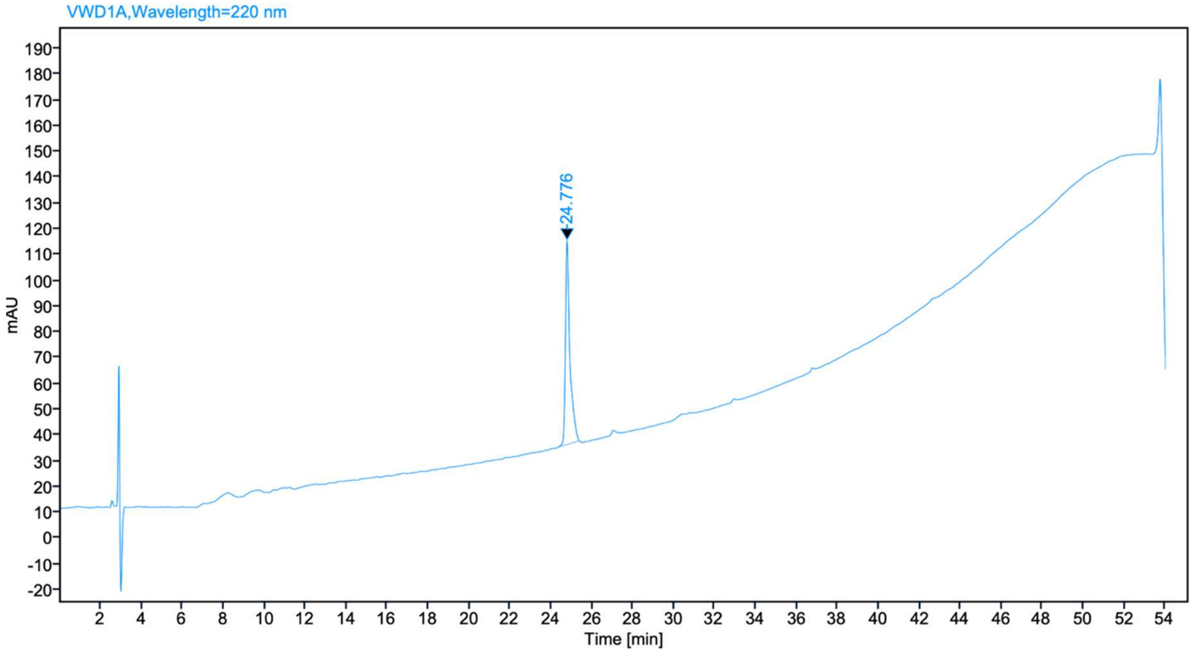

Figure S26: Analytical HPLC chromatogram of pure **C3** (retention time: 24.776 min: purity: 100%).

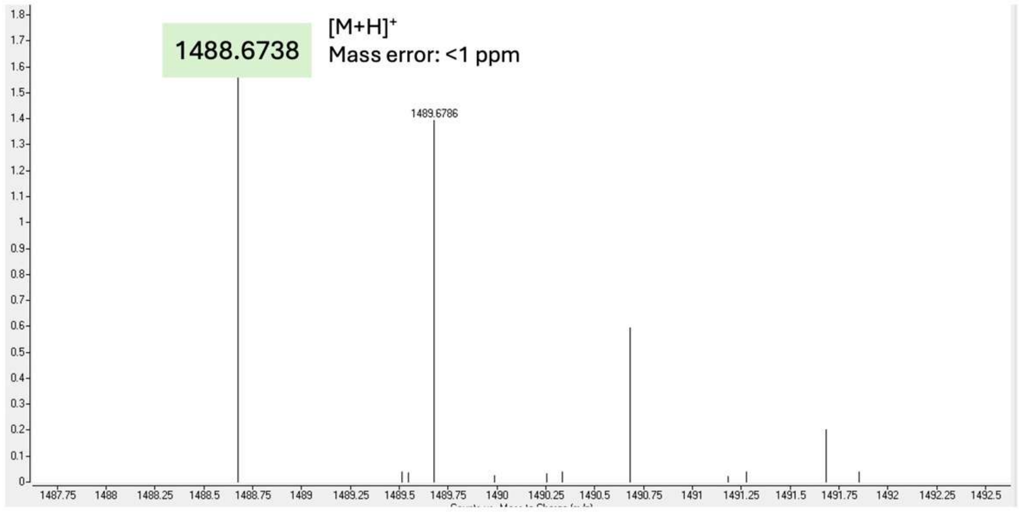

Figure S27: HRMS spectrum of **C4**, confirming the *m/z* corresponding to the exact mass of 1487.6668; Expected: 1488.6741 *m/z*, found: 1488.6738 *m/z*, mass error <1 ppm.

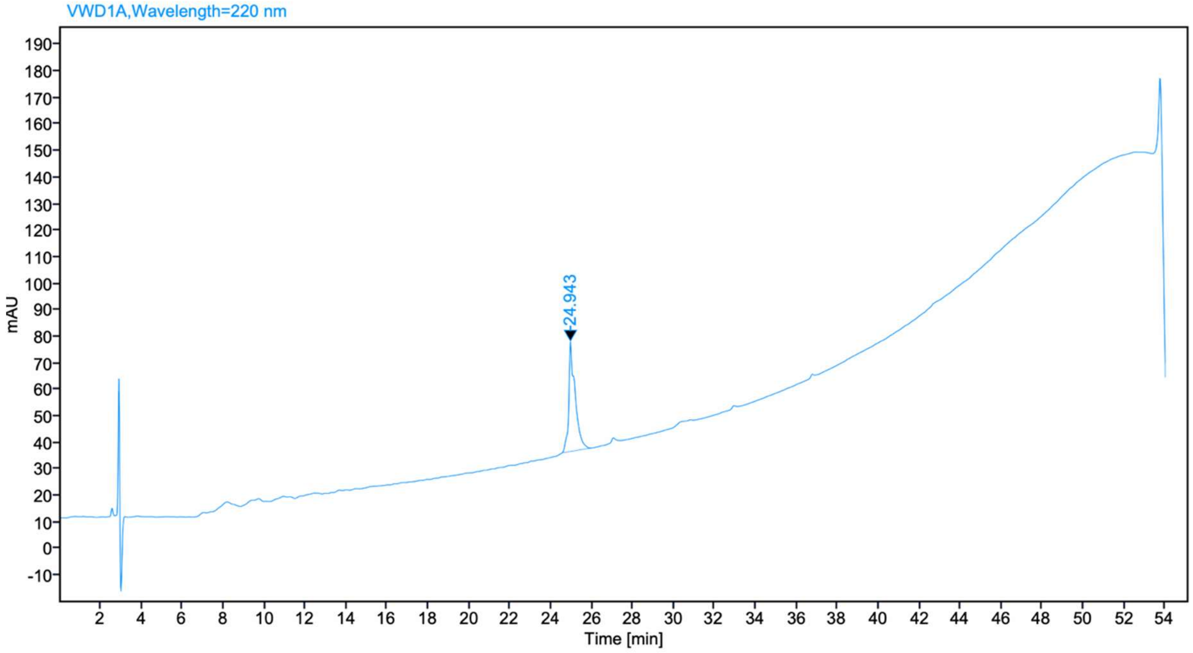

Figure S28: Analytical HPLC chromatogram of **C4** (retention time: 24.943 min).

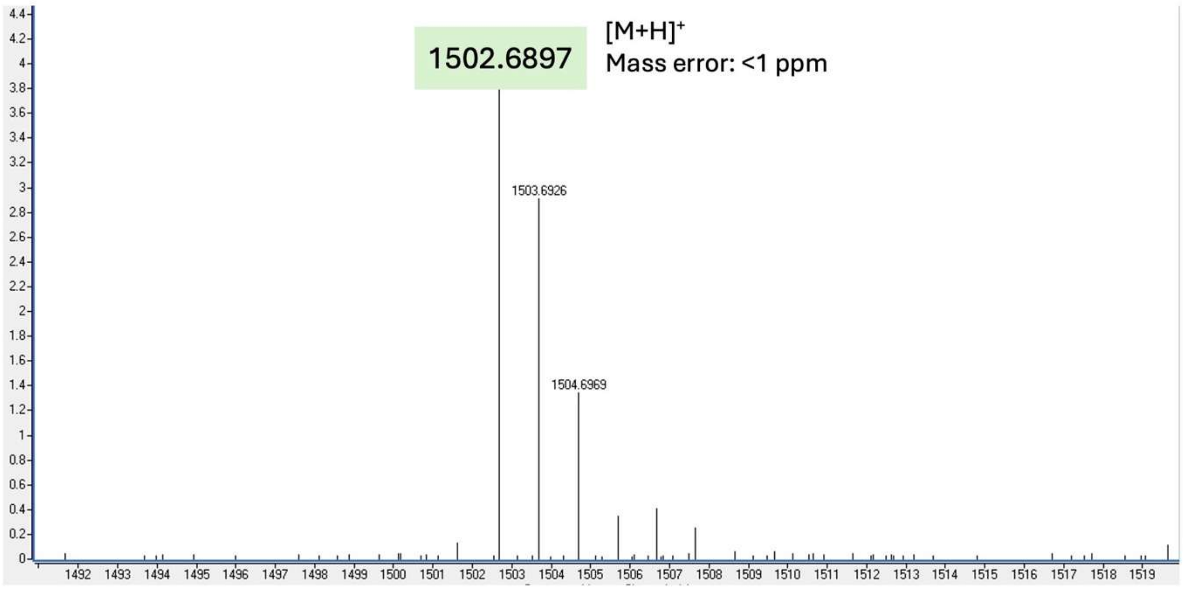

Figure S29: HRMS spectrum of pure **C5**, confirming the *m/z* corresponding to the exact mass of 1501.6824; Expected: 1502.6897 *m/z*, found: 1502.6897 *m/z*, mass error <1 ppm.

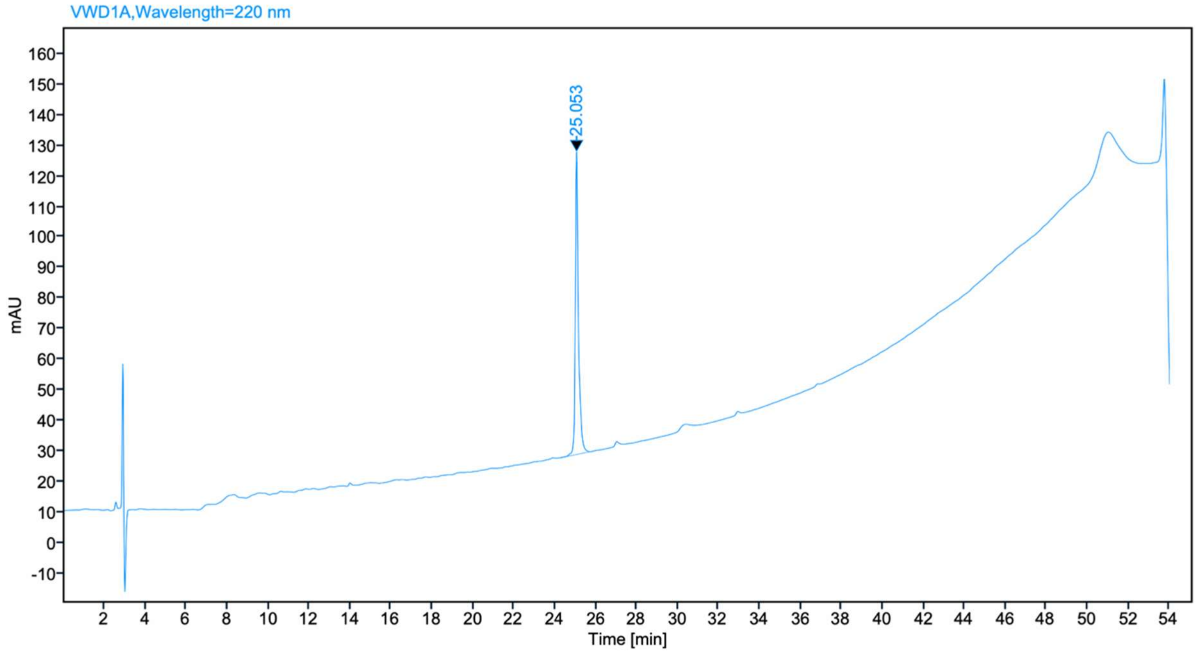

Figure S30: Analytical HPLC chromatogram of pure **C5** (retention time: 25.053 min: purity: 100%).

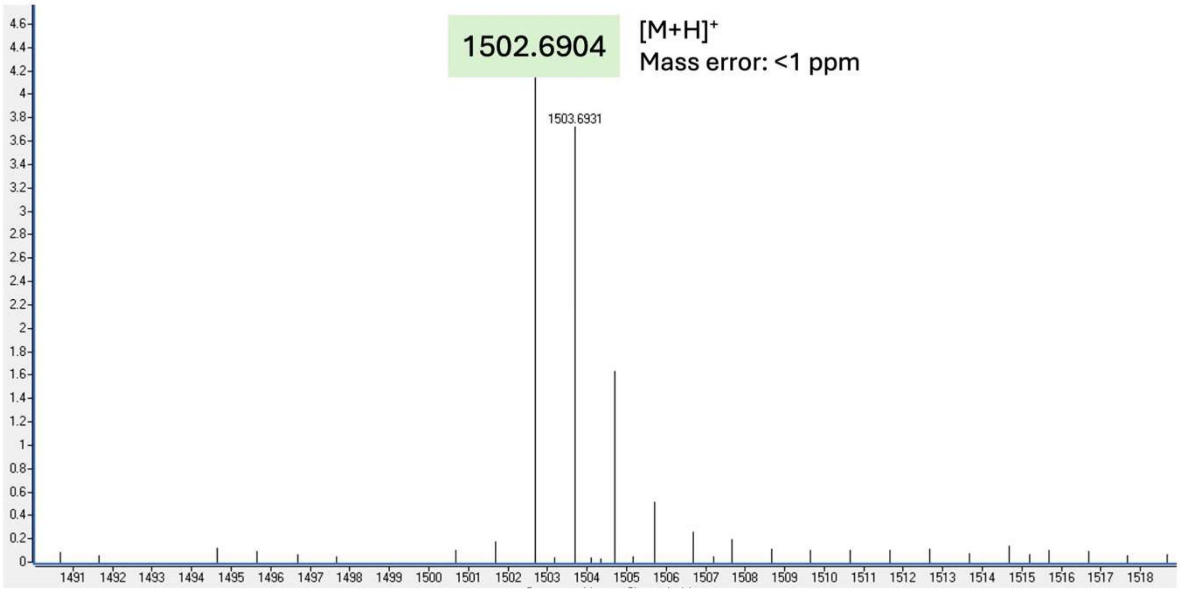

Figure S31: HRMS spectrum of pure **C6**, confirming the *m/z* corresponding to the exact mass of 1501.6824; Expected: 1502.6897 *m/z*, found: 1502.6904 *m/z*, mass error <1 ppm.

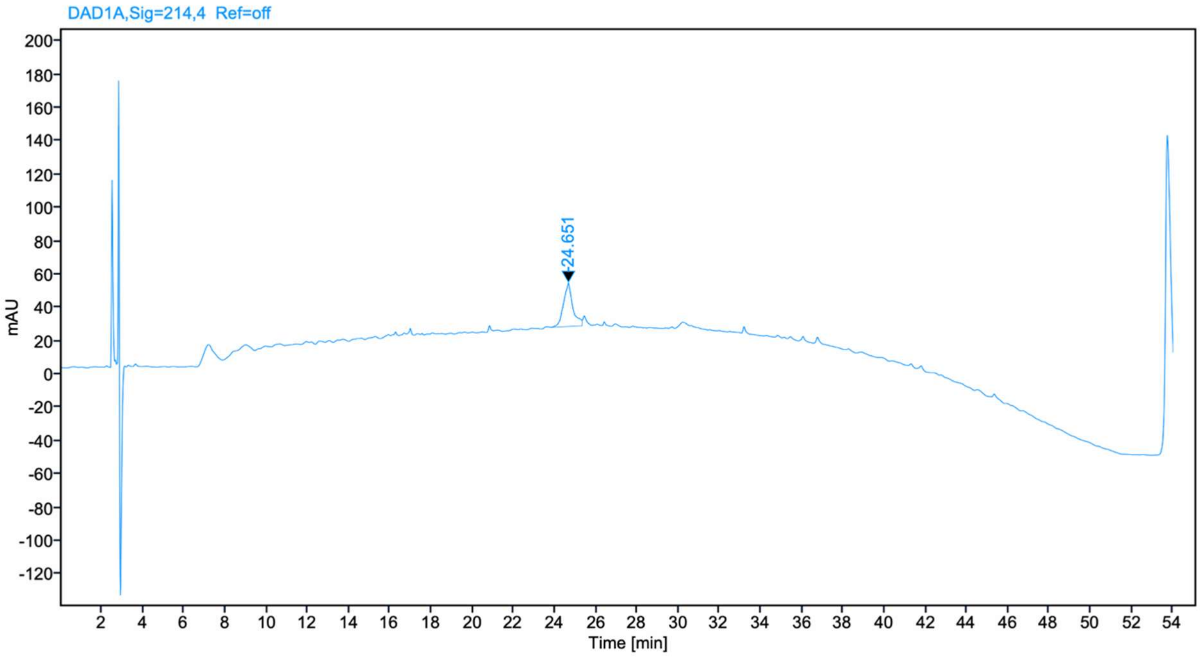

Figure S32: Analytical HPLC chromatogram of pure **C6** (retention time: 24.651 min: purity: 100%).

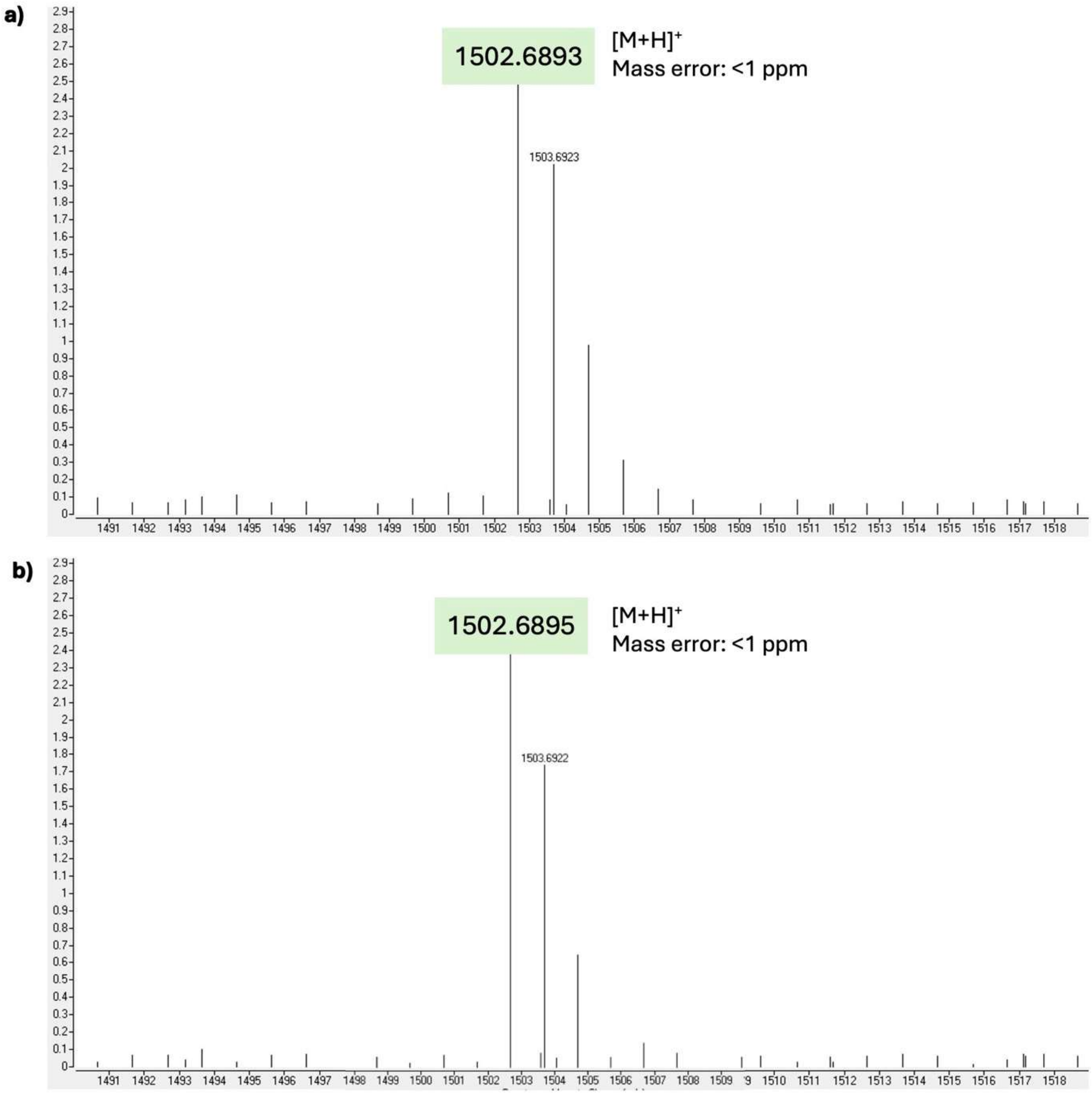

Figure S33: HRMS data for **C7**. **a)** HRMS spectrum of pure **C7a**, confirming the *m/z* corresponding to the exact mass of 1501.6824; Expected: 1502.6897 *m/z*, found: 1502.6893 *m/z*, mass error <1 ppm. **b)** HRMS spectrum of pure **C7b**, confirming the *m/z* corresponding to the exact mass of 1501.6824; Expected: 1502.6897 *m/z*, found: 1502.6895 *m/z*, mass error <1 ppm.

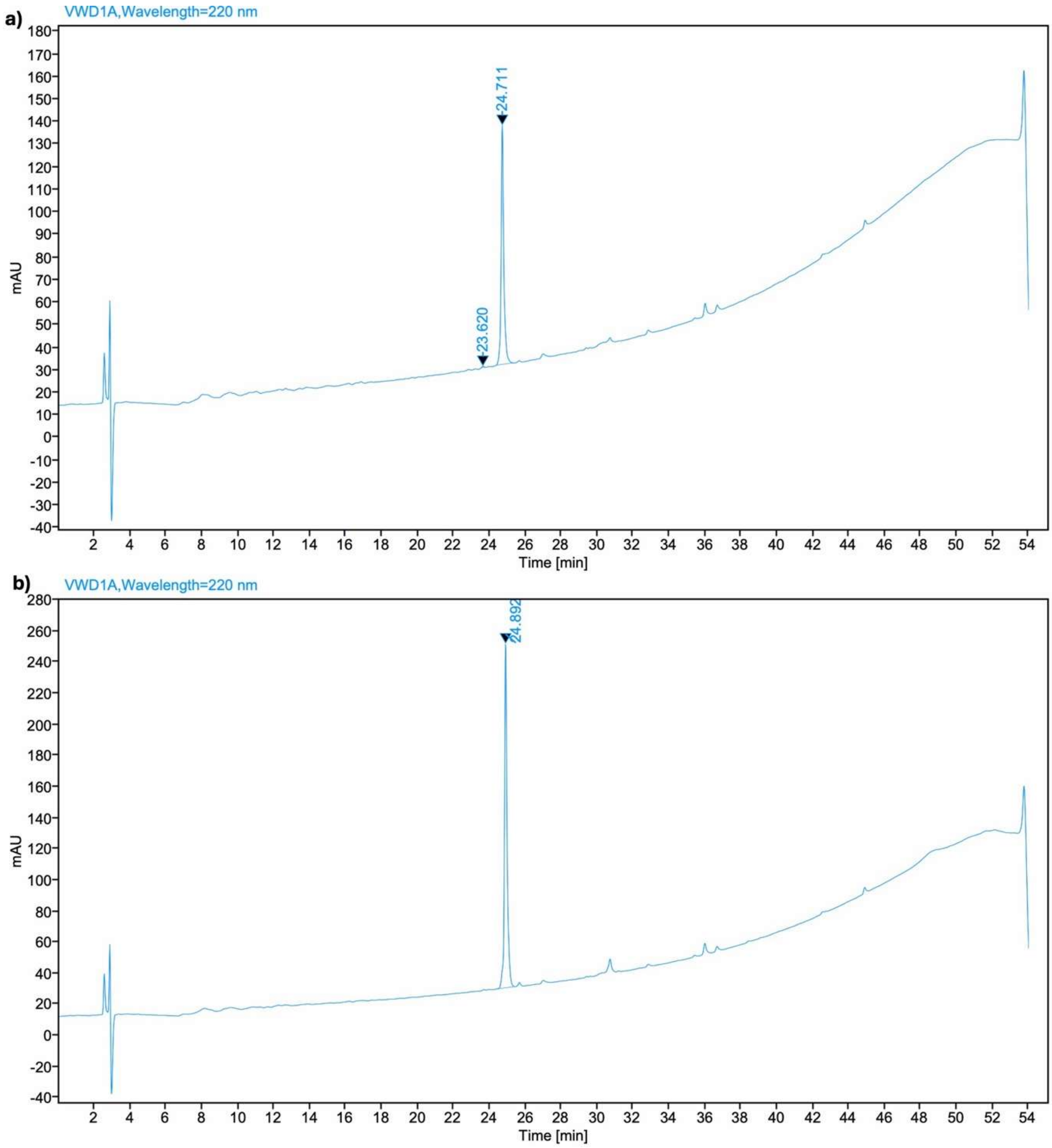

Figure S34: Analytical HPLC chromatograms of pure **a) C7a** (retention time: 24.711 min: purity: 100%); **b) C7b** (retention time: 24.892 min: purity: 100%).

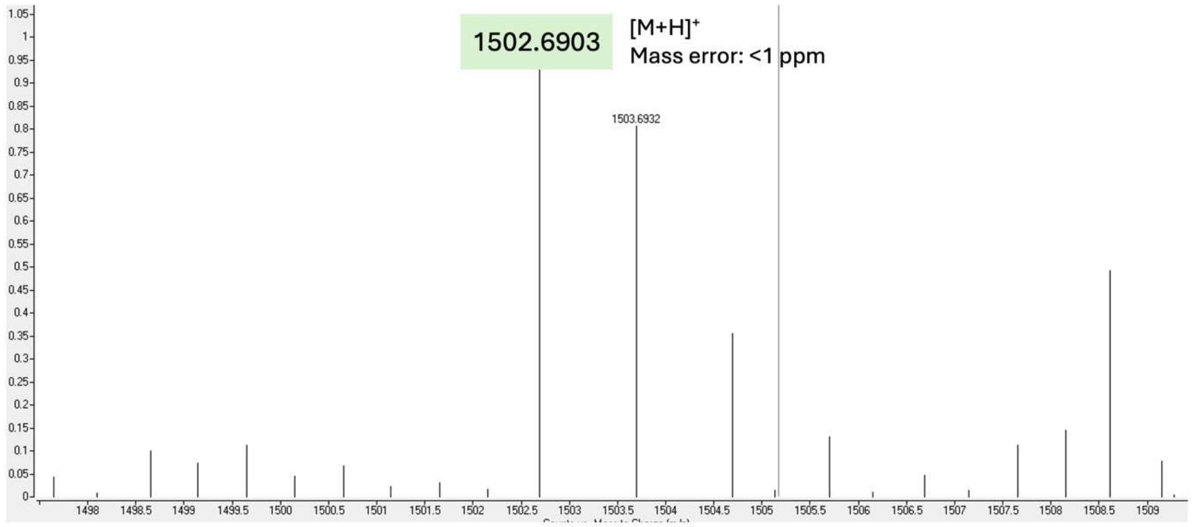

Figure S35: HRMS spectrum of pure **C8**, confirming the *m/z* corresponding to the exact mass of 1501.6824; Expected: 1502.6897 *m/z*, found: 1502.6903 *m/z*, mass error <1 ppm.

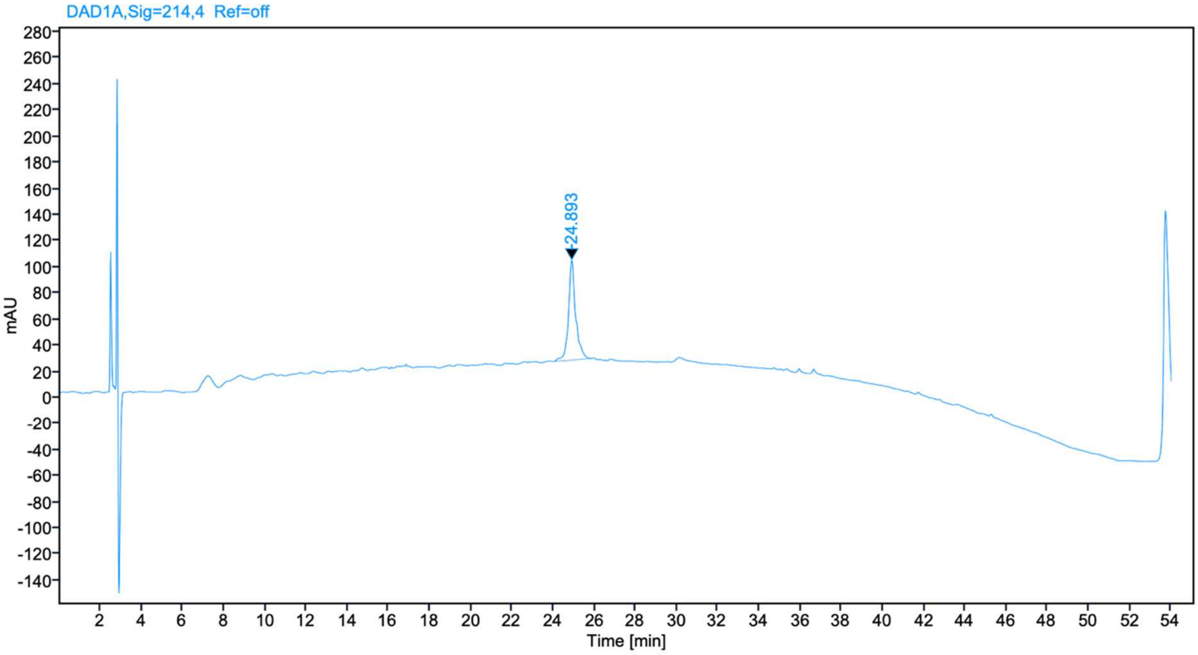

Figure S36: Analytical HPLC chromatogram of pure **C8** (retention time: 24.893 min: purity: 100%).

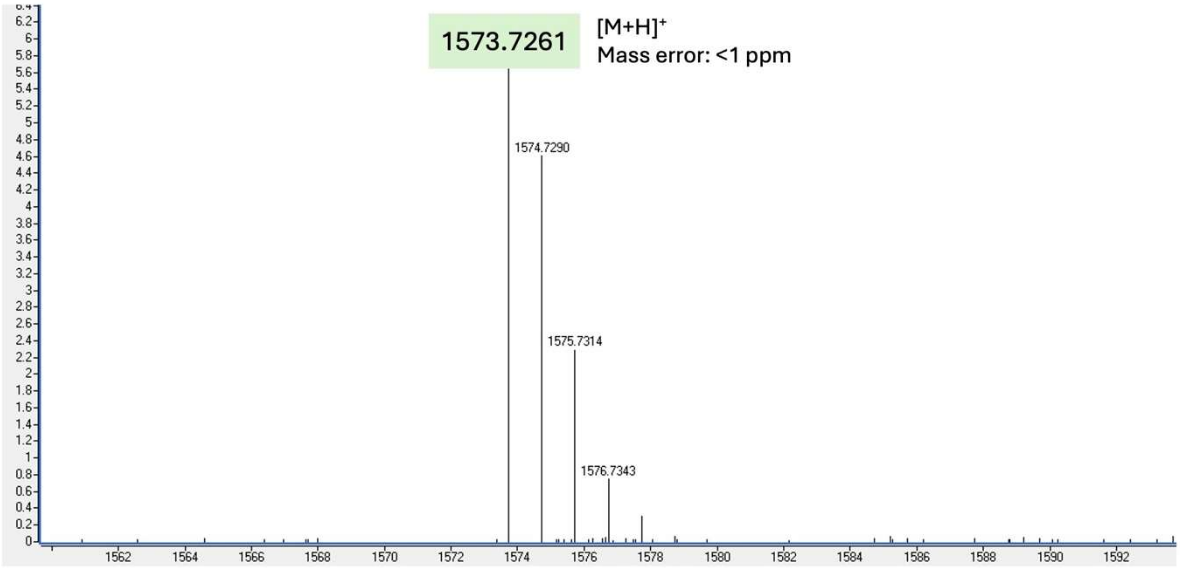

Figure S37: HRMS spectrum of pure **C9**, confirming the *m/z* corresponding to the exact mass of 1572.7195; Expected: 1573.7268 *m/z*, found: 1573.7261 *m/z*, mass error <1 ppm.

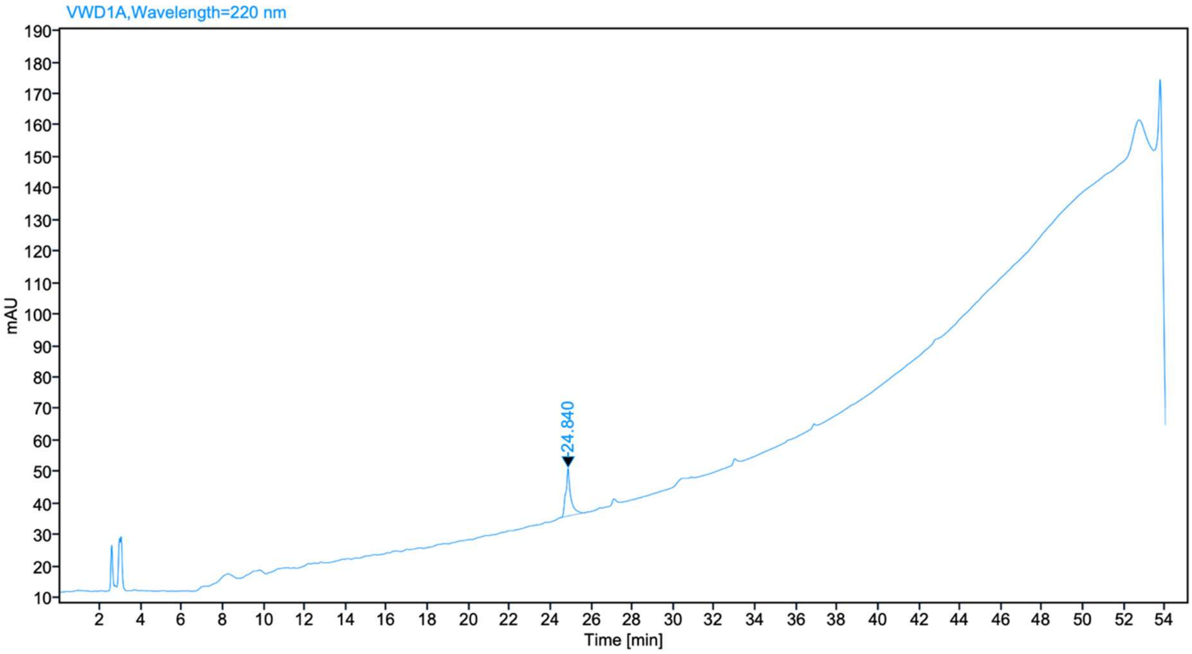

Figure S38: Analytical HPLC chromatogram of pure **C9** (retention time: 24.840 min: purity: 100%).

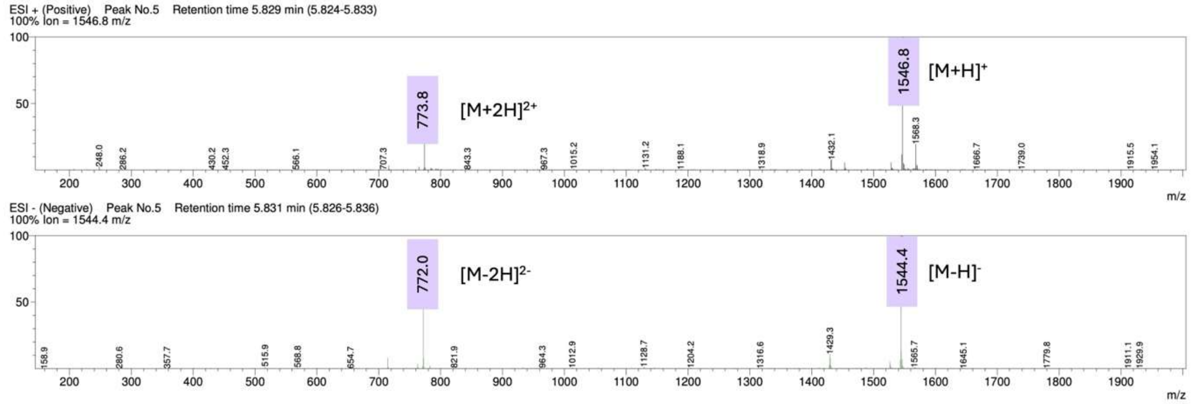

Figure S39: LC-MS spectrum of pure **C10**, confirming the *m/z* corresponding to the exact mass of 1544.6882; Expected: $[M+H]^+$ 1545.7 *m/z*, $[M+H]^{2+}$ 773.5, $[M-H]^-$ 1543.7 *m/z*, $[M-H]^{2-}$ 771.3 *m/z*, found: $[M+H]^+$ 1546.8 *m/z*, $[M+H]^{2+}$ 773.8, $[M-H]^-$ 1544.4 *m/z*, $[M-H]^{2-}$ 772.0 *m/z*.

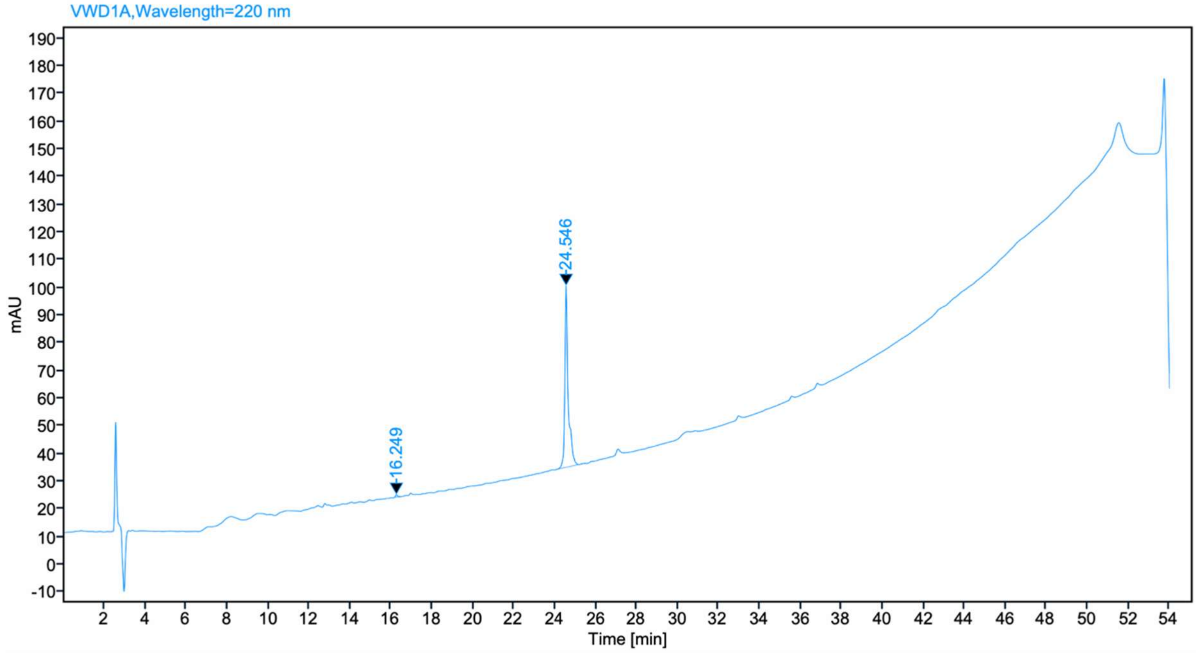

Figure S40: Analytical HPLC chromatogram of pure **C10** (retention time: 24.546 min: purity: 99%).

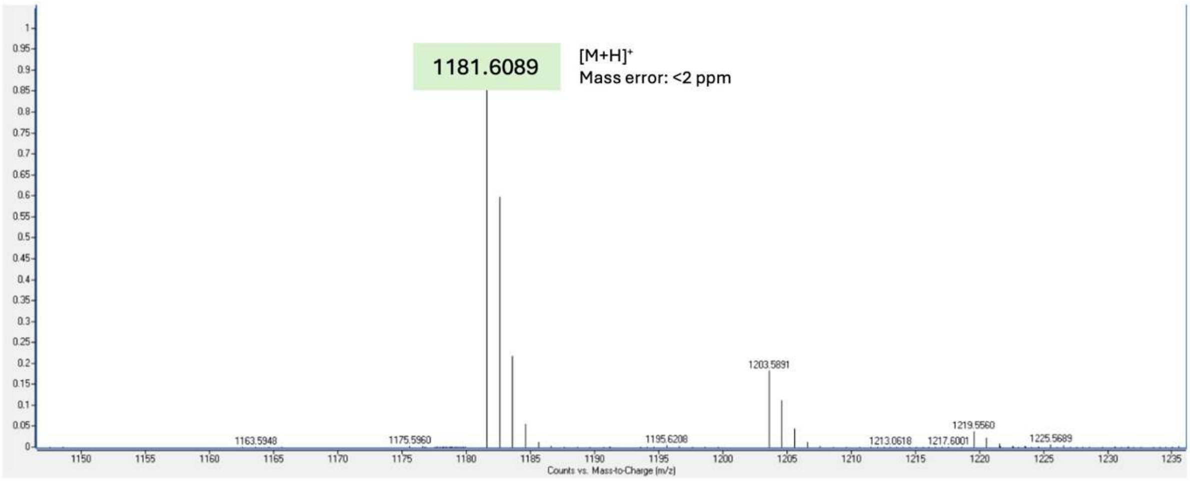

Figure S41: HRMS spectrum of pure **M1**, confirming the *m/z* corresponding to the exact mass of 1180.5976; Expected: 1181.6049 *m/z*, found: 1181.6089 *m/z*, mass error <2 ppm.

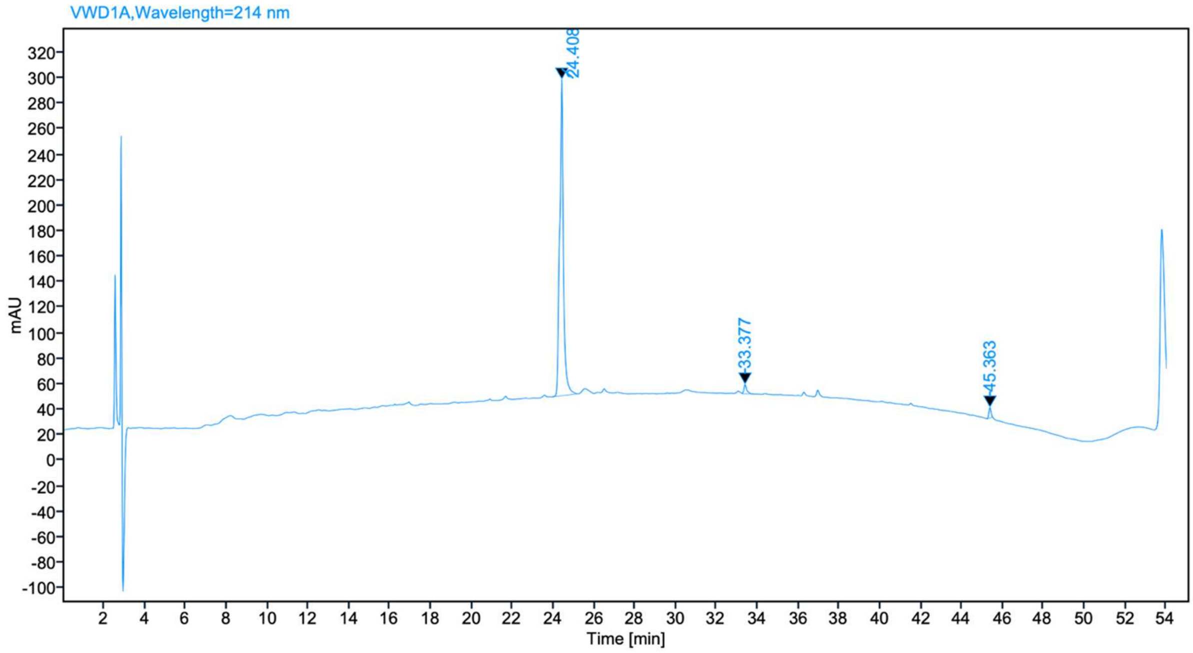

Figure S42: Analytical HPLC chromatogram of pure **M1** (retention time: 24.408 min: purity: 96%).

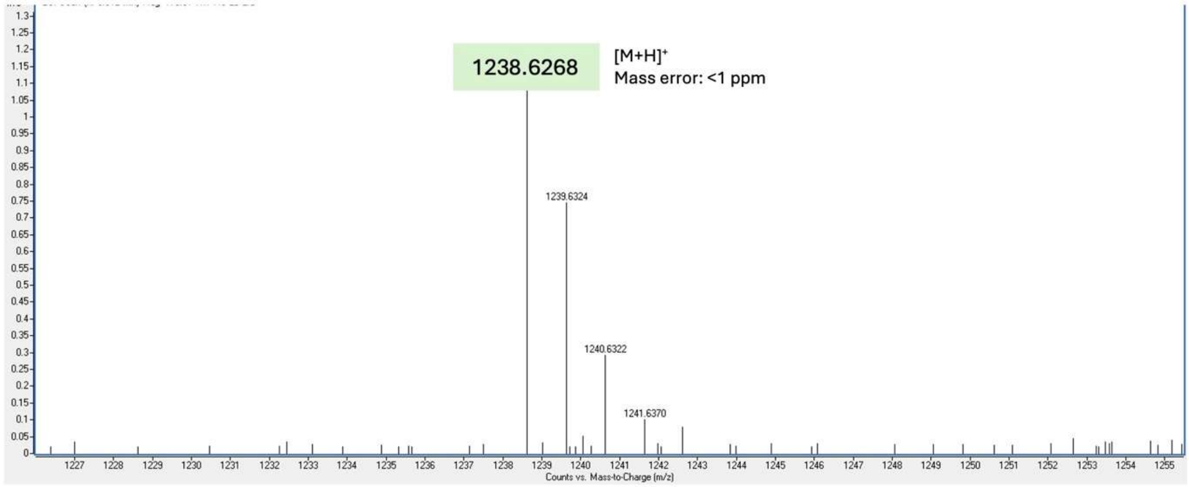

Figure S43: HRMS spectrum of pure **M2**, confirming the *m/z* corresponding to the exact mass of 1237.6190; Expected: 1238.6263 *m/z*, found: 1238.6268 *m/z*, mass error <1 ppm.

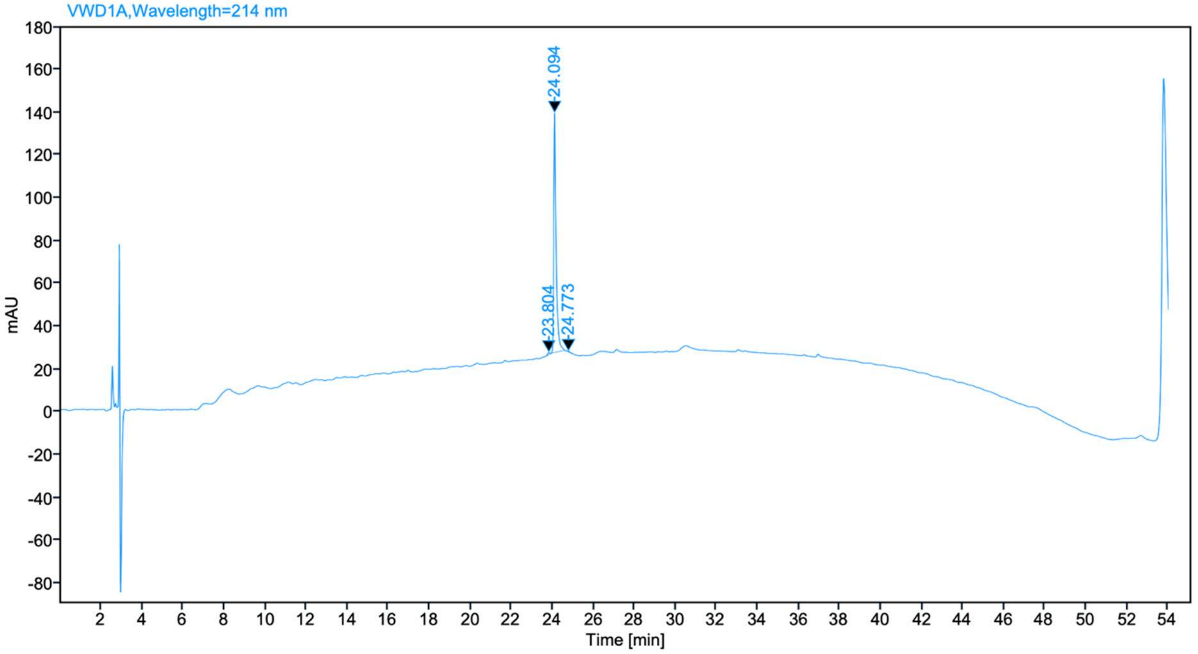

Figure S44: Analytical HPLC chromatogram of pure **M2** (retention time: 24.094 min: purity: 99%).

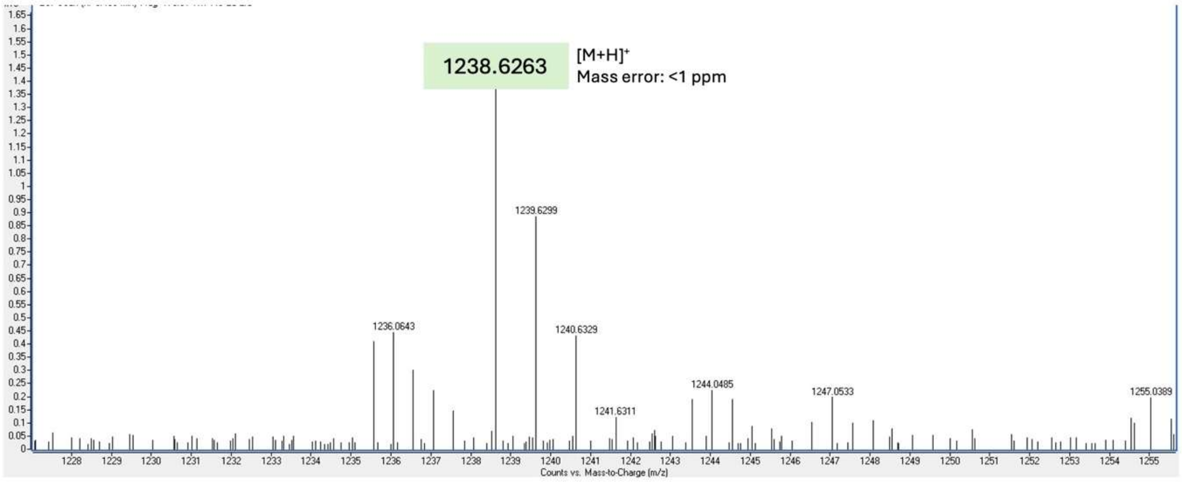

Figure S45: HRMS spectrum of pure **M3**, confirming the *m/z* corresponding to the exact mass of 1237.6190; Expected: 1238.6263 *m/z*, found: 1238.6263 *m/z*, mass error <1 ppm.

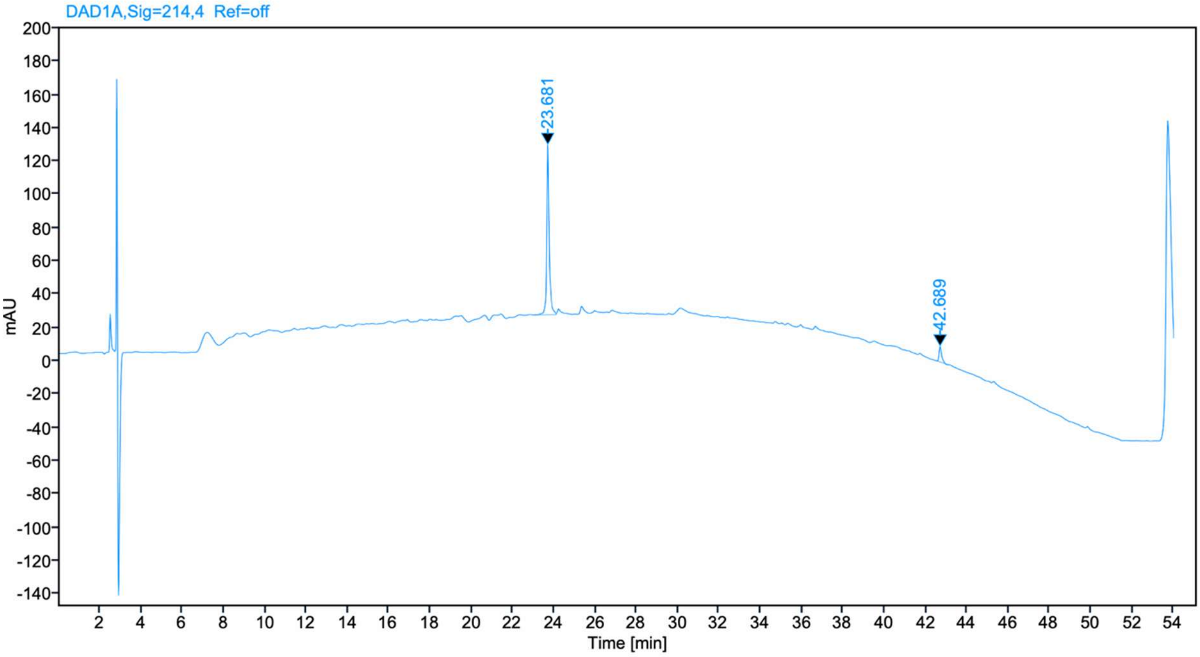

Figure S46: Analytical HPLC chromatogram of pure **M3** (retention time: 23.681 min: purity: 91%).

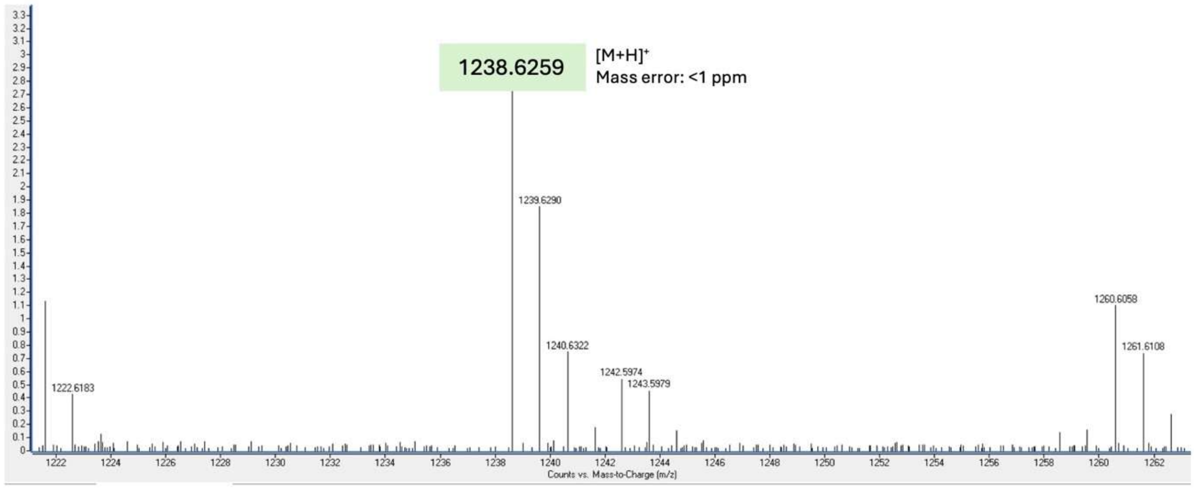

Figure S47: HRMS spectrum of pure **M4**, confirming the *m/z* corresponding to the exact mass of 1237.6190; Expected: 1238.6263 *m/z*, found: 1238.6259 *m/z*, mass error <1 ppm.

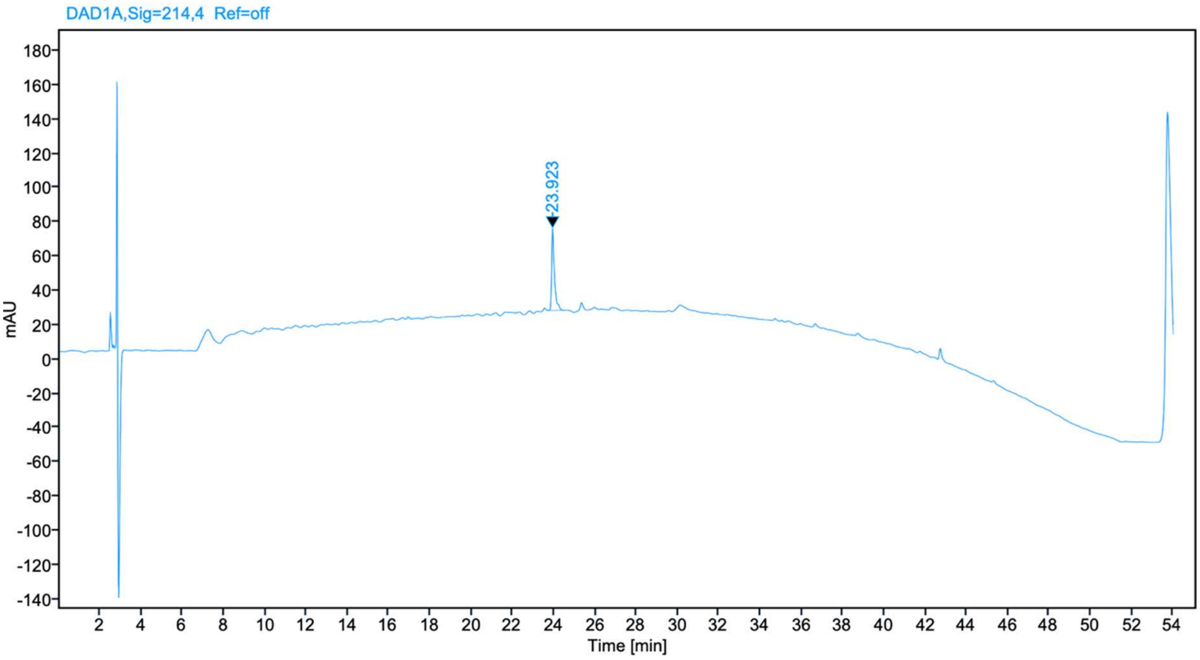

Figure S48: Analytical HPLC chromatogram of pure **M4** (retention time: 23.923 min: purity: 100%).

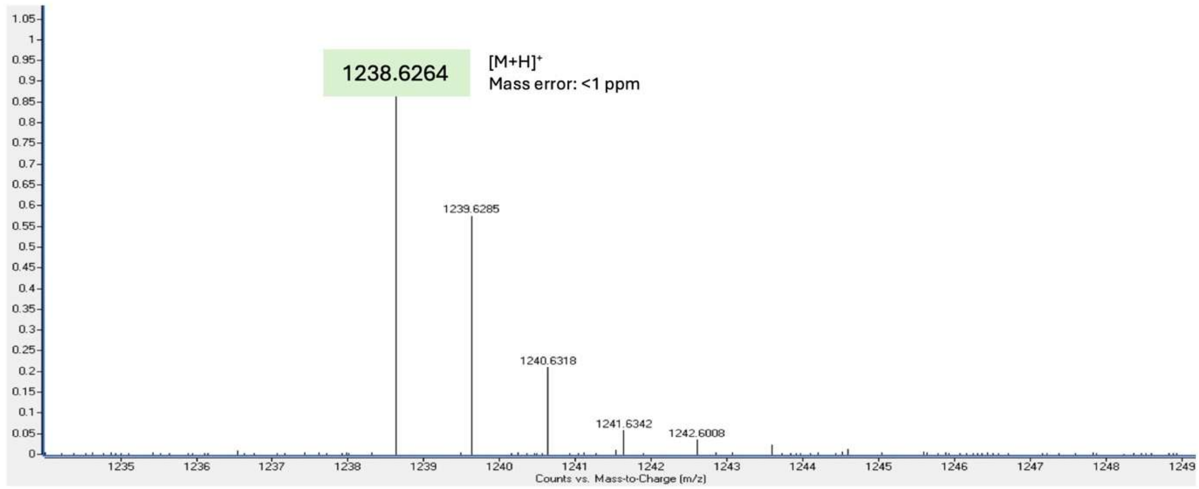

Figure S49: HRMS spectrum of pure **M5**, confirming the *m/z* corresponding to the exact mass of 1237.6190; Expected: 1238.6263 *m/z*, found: 1238.6264 *m/z*, mass error <1 ppm.

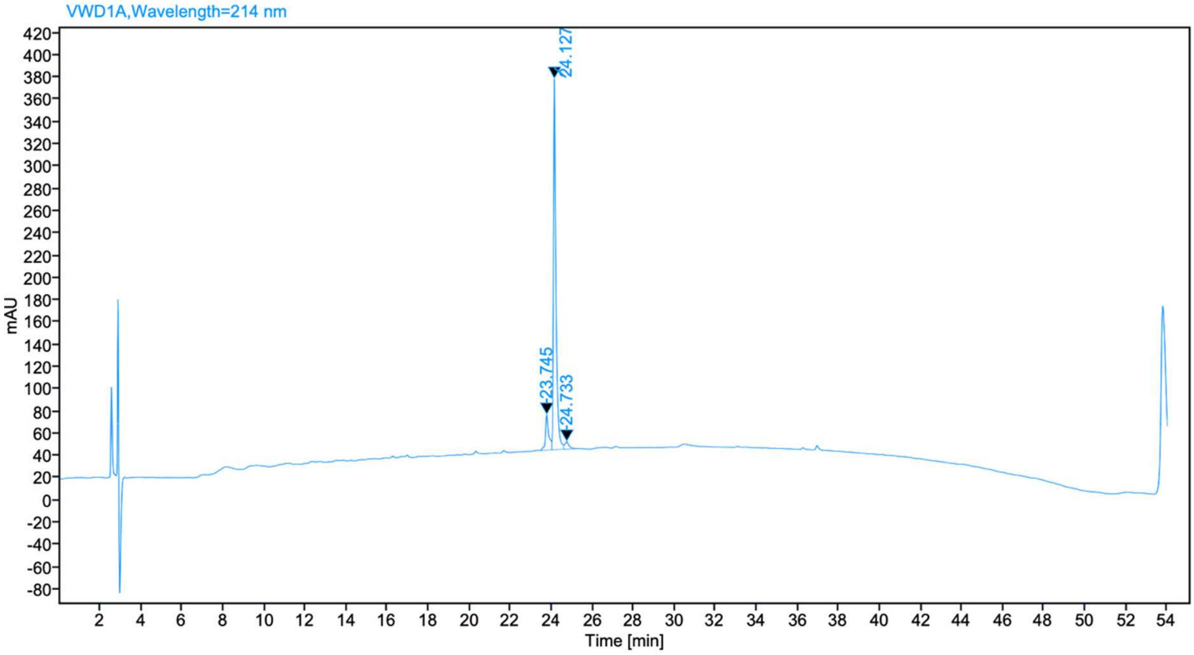

Figure S50: Analytical HPLC chromatogram of pure **M5** retention time: 24.127 min: purity: 86%).

(3*S*)-3-(allyloxycarbonyl)amino-2-methylpropanoic acid (11) (Alloc-L-BAIBA-OH)

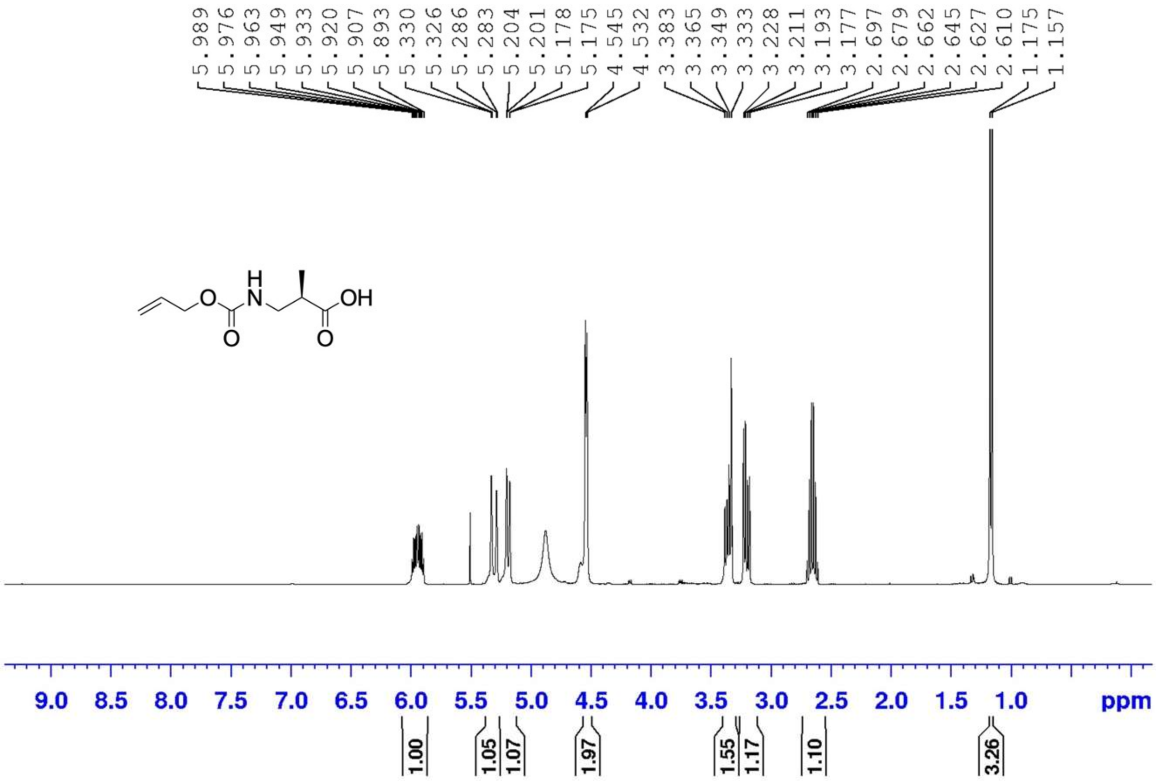

Figure S51: ^{1}H spectrum of Alloc-L-*β*-amino isobutyric acid-OH (**11**) (CD_3OD).

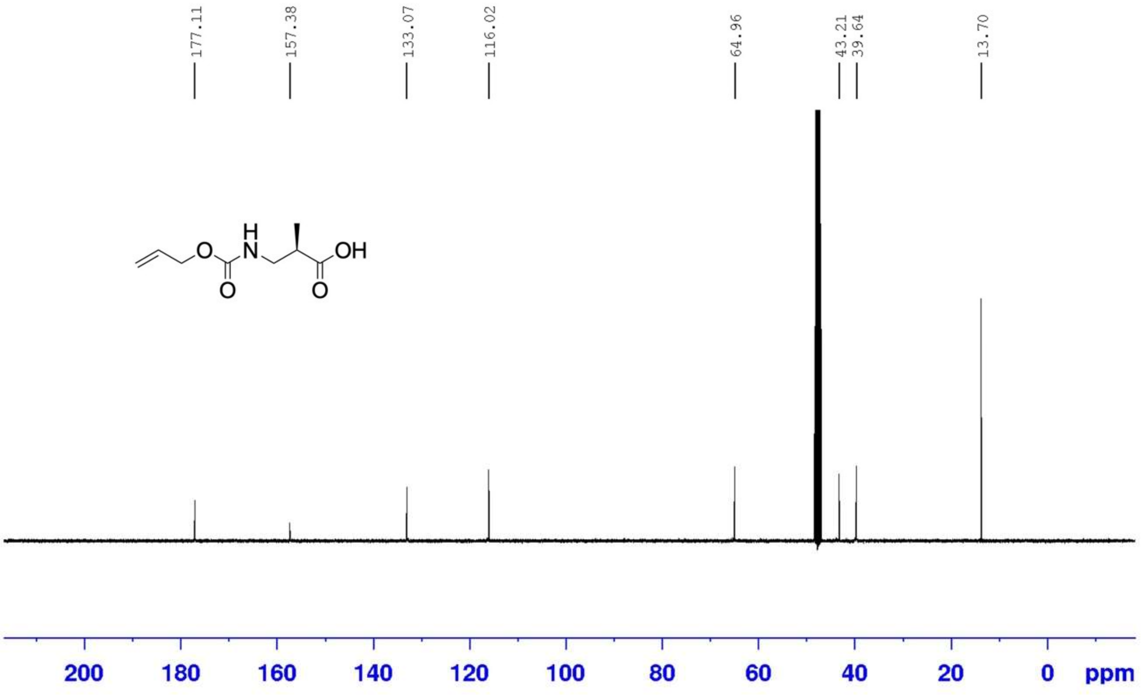

Figure S52: ^{13}C spectrum of Alloc-L-*β*-amino isobutyric acid-OH (**11**) (CD_3OD).

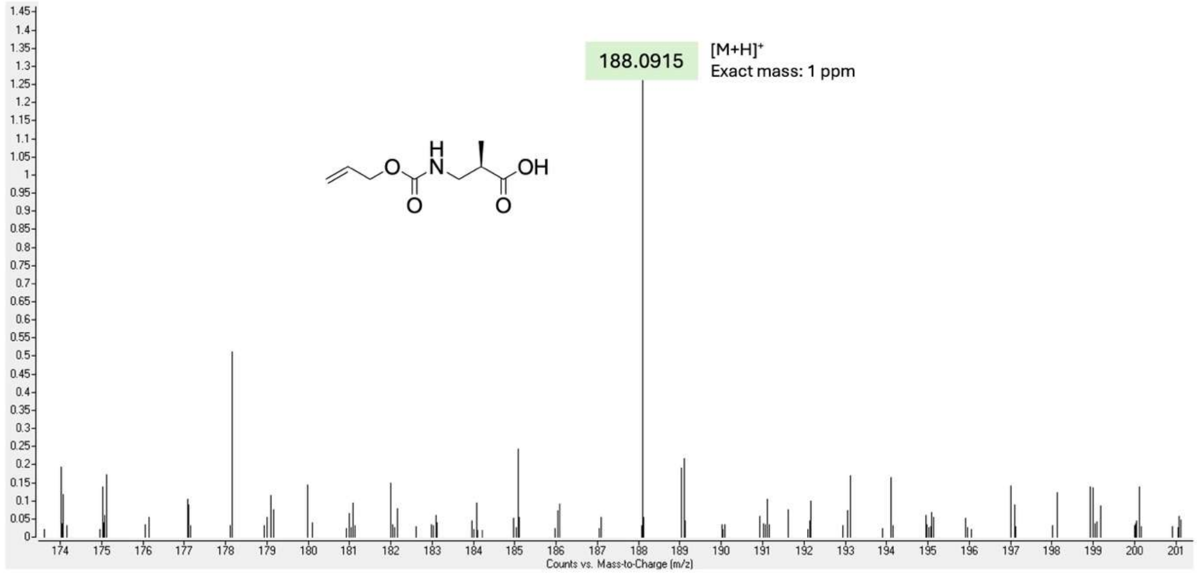

Figure S53: HRMS spectrum of pure **11**, confirming the *m/z* corresponding to the exact mass of 187.0845; Expected: 188.0917 *m/z*, found: 188.0915 *m/z*, mass error 1 ppm.

3-(allyloxycarbonyl)amino-2-methylpropanoic acid (12) (Teoc-L-BAIBA-OH)

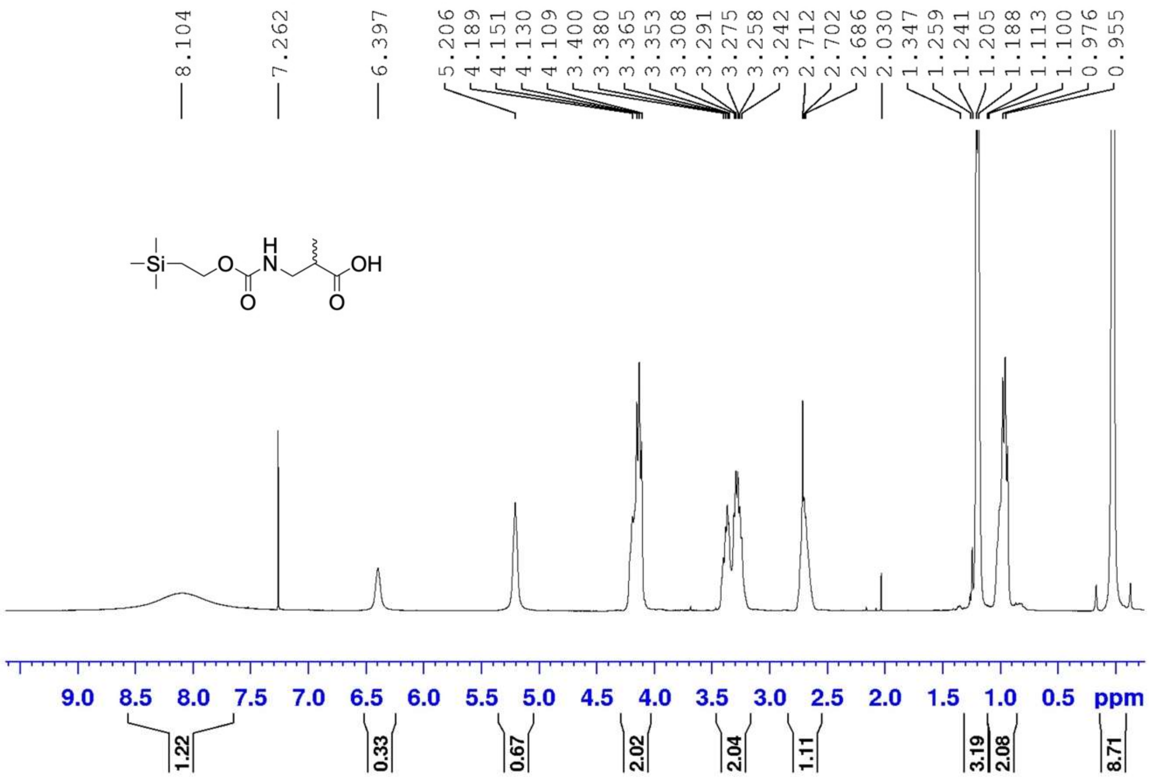

Figure S54: ^{1}H spectrum of Teoc-L-*β*-amino isobutyric acid-OH (**12**) ($CDCl_3$).

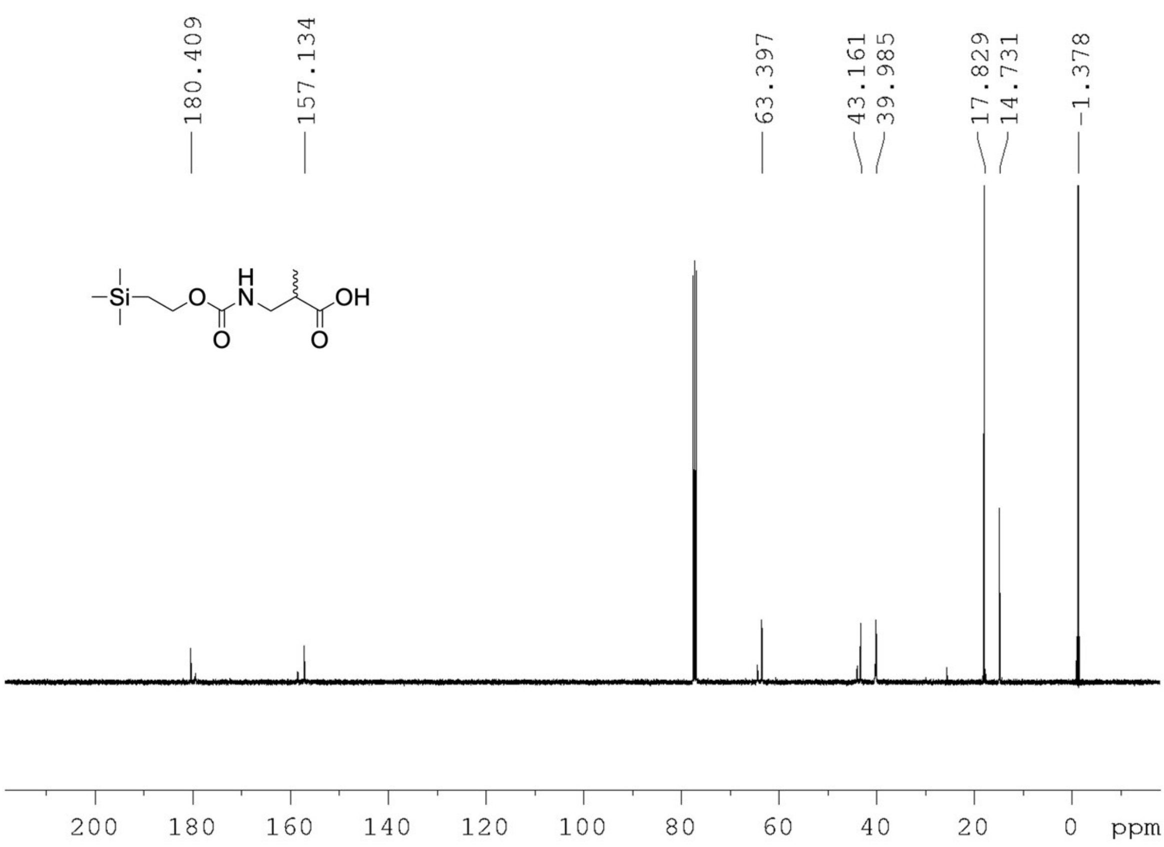

Figure S55: ^{13}C spectrum of Teoc-L-β-amino isobutyric acid-OH (**12**) ($CDCl_3$).

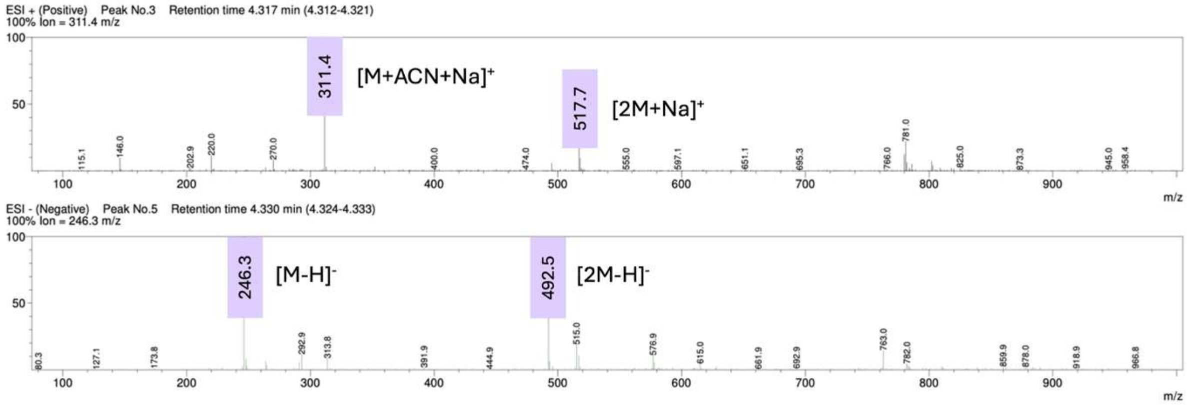

Figure S56: LC-MS spectrum of pure **12**, confirming the *m/z* corresponding to the exact mass of 247.1240; Expected: $[2M+Na]^+$ 517.2 *m/z*, $[M+ACN+NA]^+$ 311.1, $[2M-H]^-$ 493.2 *m/z*, $[M-H]^-$ 246.1 *m/z*, found: $[2M+Na]^+$ 517.7 *m/z*, $[M+ACN+NA]^+$ 311.4, $[2M-H]^-$ 492.5 *m/z*, $[M-H]^-$ 246.3 *m/z*.

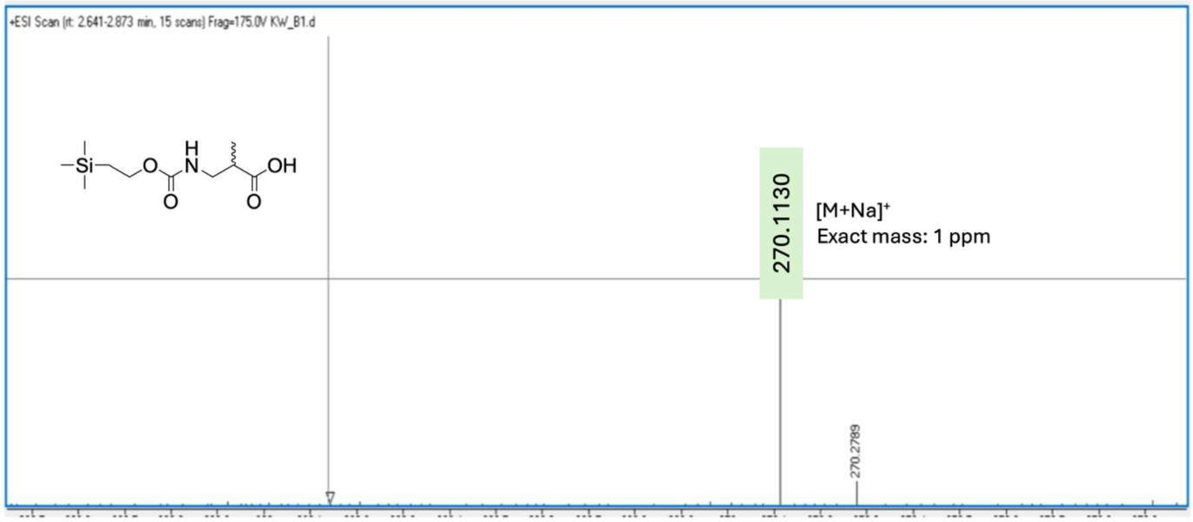

Figure S57: HRMS spectrum (ESI+) of **12**, confirming the *m/z* corresponding to the exact mass of 247.1240; Expected: 270.1132 *m/z*, found: 270.1130 *m/z*, mass error 1 ppm.

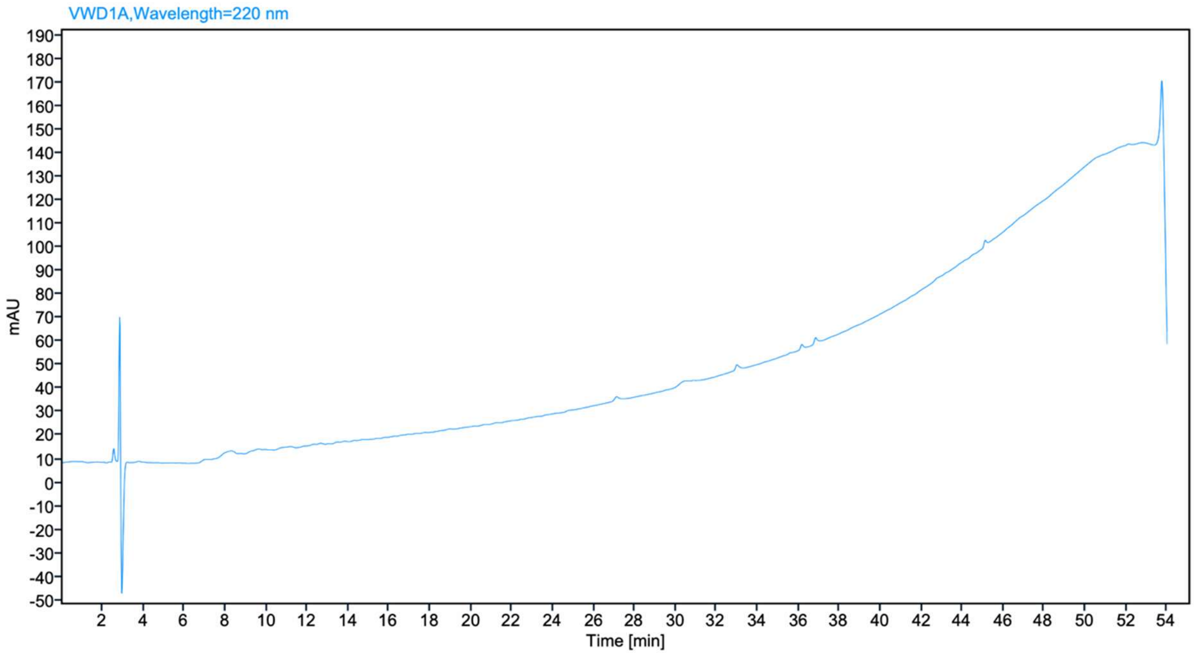

Figure S58: Analytical HPLC chromatogram at 220 nm of the blank measurement used for analysis of HPLC results for **C3, C4**.

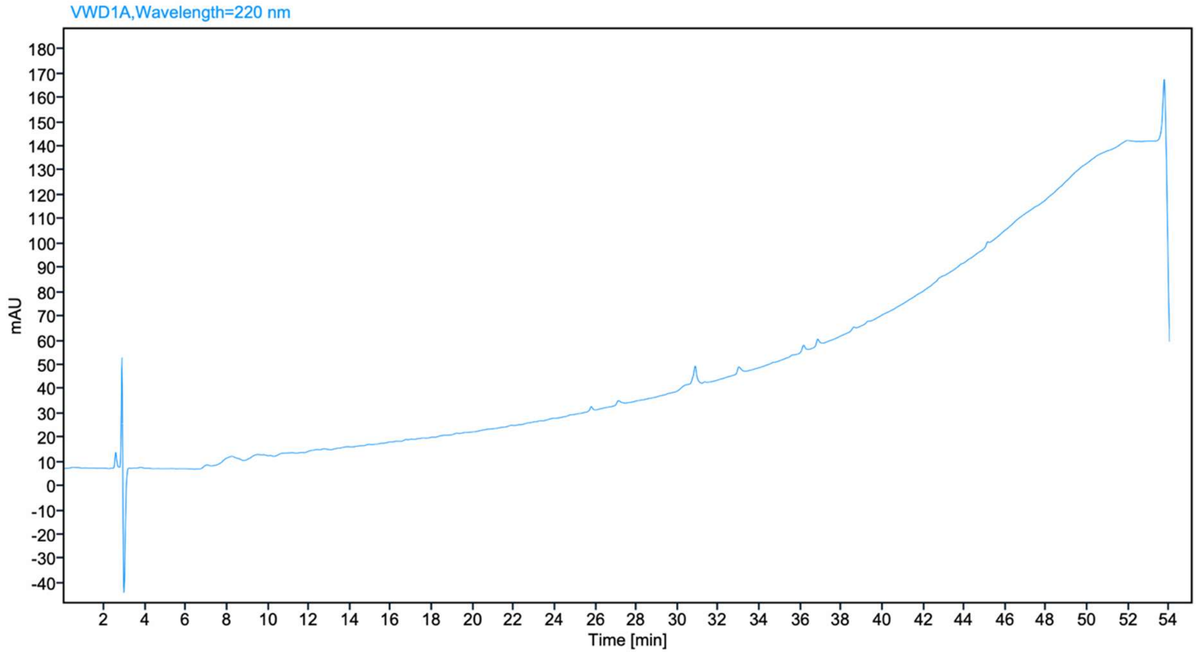

Figure S59: Analytical HPLC chromatogram at 220 nm of the blank measurement used for analysis of HPLC results for **C2**, **C9**.

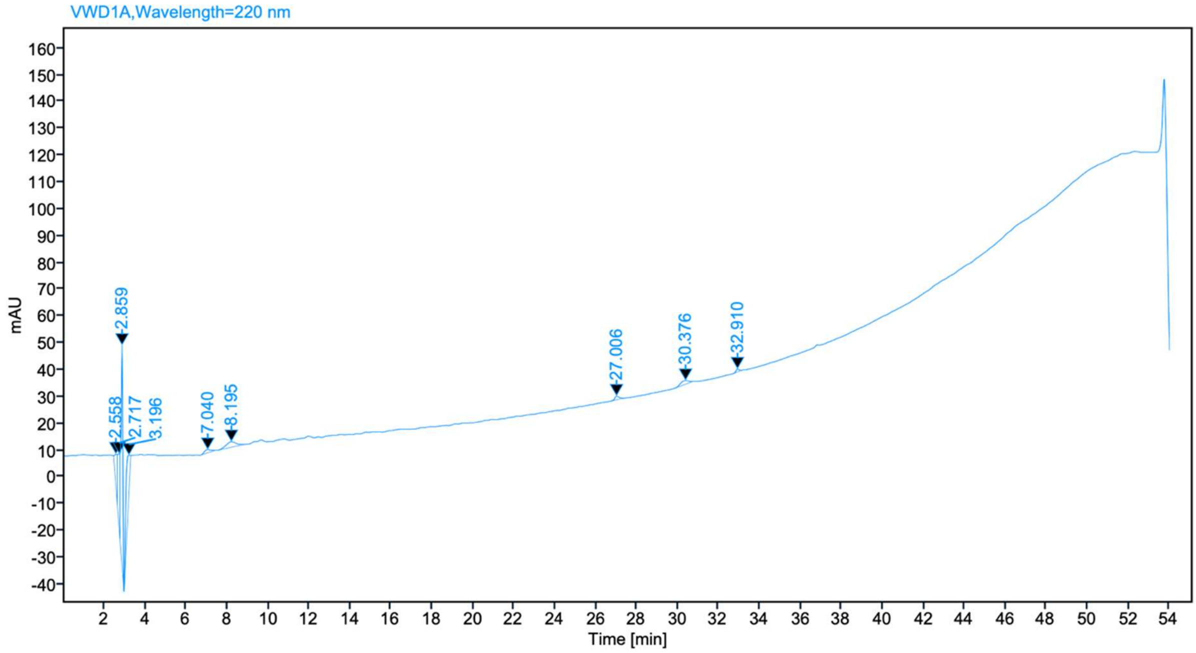

Figure S60: Analytical HPLC chromatogram at 220 nm of the blank measurement used for analysis of HPLC results for **C6**.

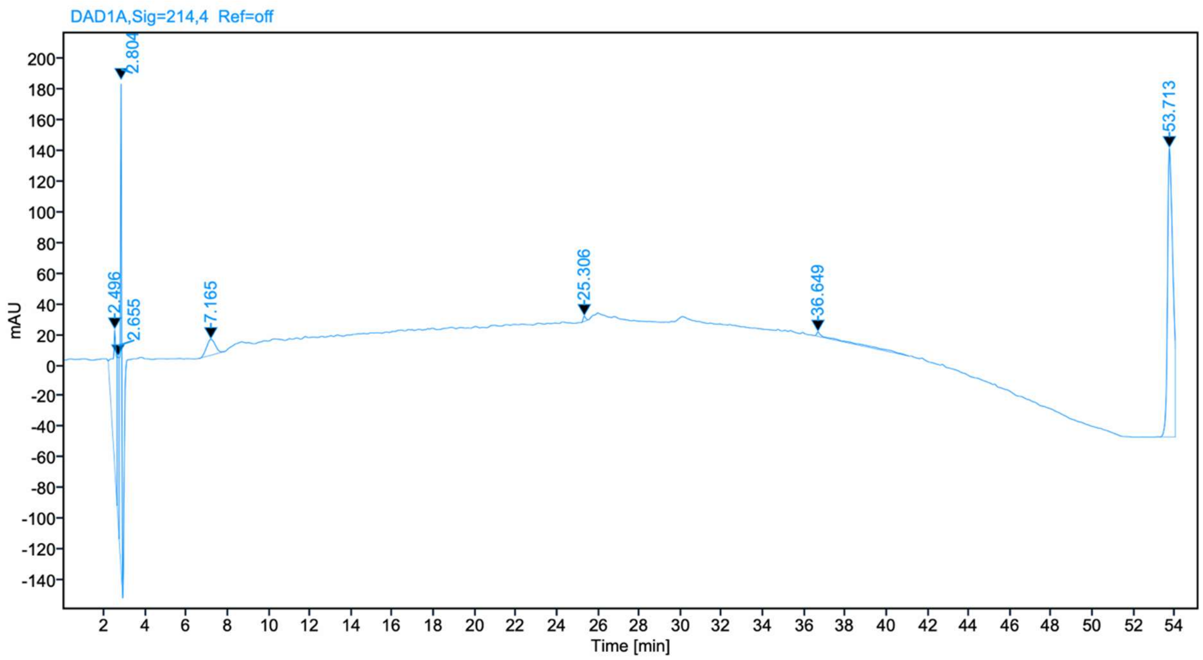

Figure S61: Analytical HPLC chromatogram at 220 nm of the blank measurement used for analysis of HPLC results for **C5**, **C8**.

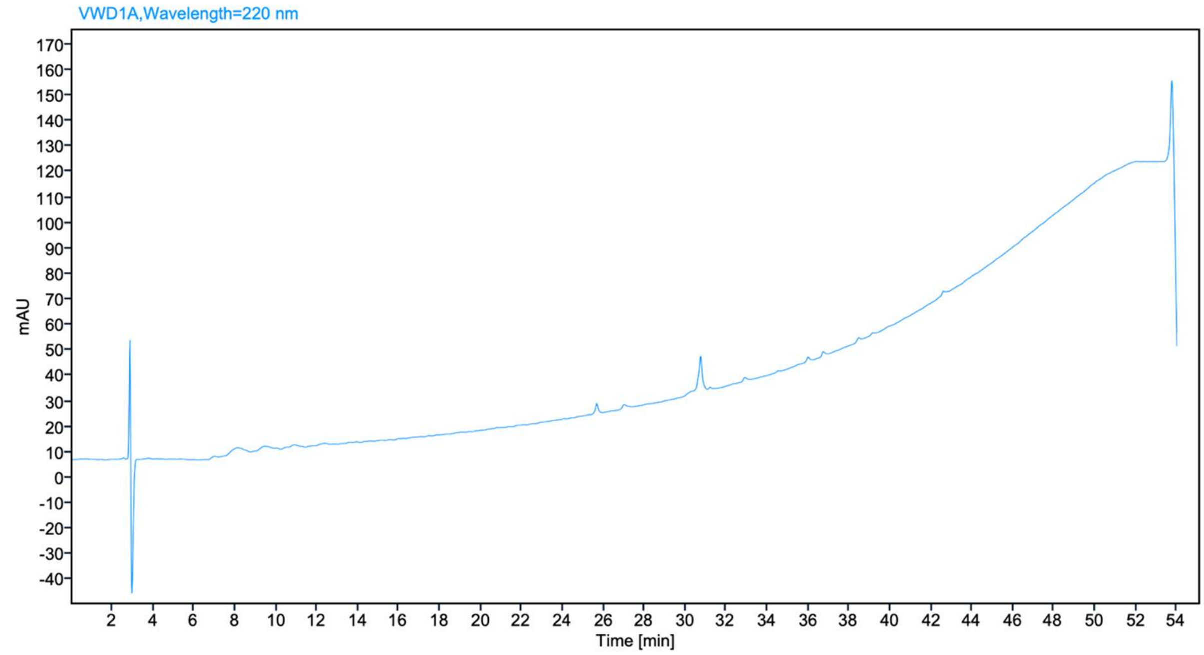

Figure S62: Analytical HPLC chromatogram at 220 nm of the blank measurement used for analysis of HPLC results for **C7**.

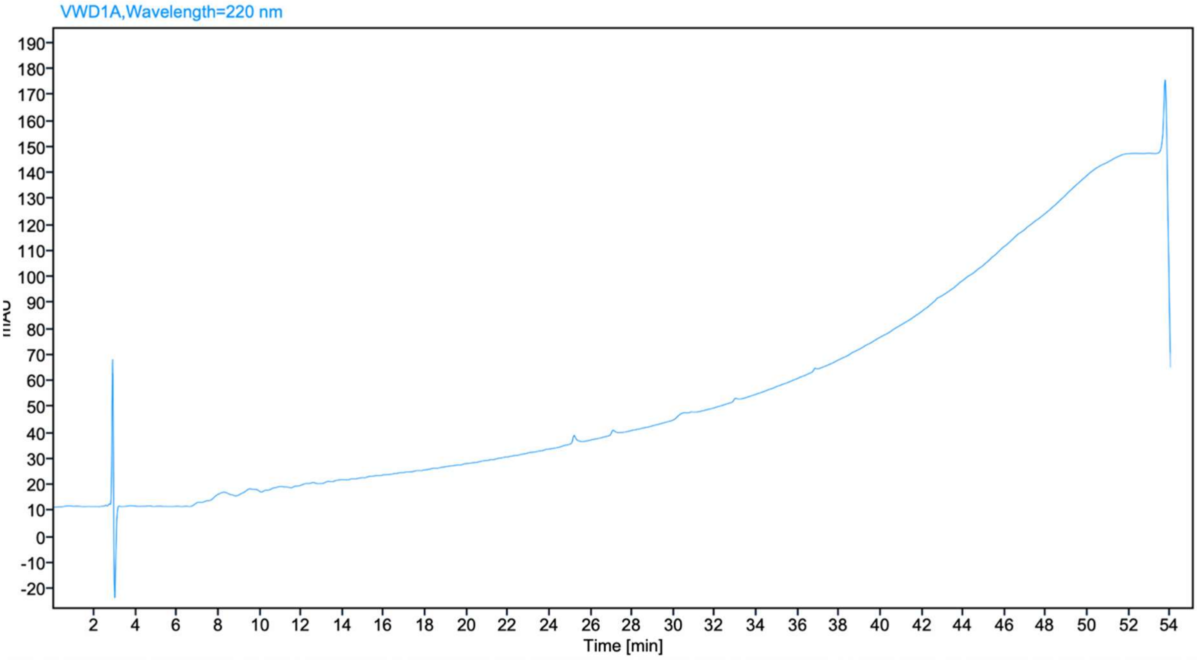

Figure S63: HPLC spectrum at 220 nm of the blank measurement used for analysis of HPLC results for **C1, C10**.

7. References

1. Grein, F.; Müller, A.; Scherer, K. M.; Liu, X.; Ludwig, K. C.; Klöckner, A.; Strach, M.; Sahl, H.-G.; Kubitscheck, U.; Schneider, T. Ca2+-Daptomycin Targets Cell Wall Biosynthesis by Forming a Tripartite Complex with Undecaprenyl-Coupled Intermediates and Membrane Lipids. Nat. Commun. 2020, 11 (1), 1455. https://doi.org/10.1038/s41467-020-15257-1
2. Srivastava, D.; Patra, N. Elucidating Daptomycin's Antibacterial Efficacy: Insights into the Tripartite Complex with Lipid II and Phospholipids in Bacterial Septum Membrane. J. Phys. Chem. B 2024, 128 (18), 4414–4427. https://doi.org/10.1021/acs.jpcb.4c00332
3. Silverman, J. A.; Perlmutter, N. G.; Shapiro, H. M. Correlation of Daptomycin Bactericidal Activity and Membrane Depolarization in Staphylococcus Aureus. Antimicrob. Agents Chemother. 2003, 47 (8), 2538–2544. https://doi.org/10.1128/AAC.47.8.2538-2544.2003
4. Oluwole, A. O.; Kalmankar, N. V; Guida, M.; Bennett, J. L.; Poce, G.; Bolla, J. R.; Robinson, C. V. Lipopeptide Antibiotics Disrupt Interactions of Undecaprenyl Phosphate with UptA. Proc. Natl. Acad. Sci. 2024, 121 (41), e2408315121. https://doi.org/10.1073/pnas.2408315121
5. Zhang, T.; Taylor, S. D.; Palmer, M.; Duhamel, J. Membrane Binding and Oligomerization of the Lipopeptide A54145 Studied by Pyrene Fluorescence. Biophys. J. 2016, 111 (6), 1267–1277. https://doi.org/https://doi.org/10.1016/j.bpj.2016.07.018
6. Kleijn, L. H. J.; Oppedijk, S. F.; 't Hart, P.; van Harten, R. M.; Martin-Visscher, L. A.; Kemmink, J.; Breukink, E.; Martin, N. I. Total Synthesis of Laspartomycin C and Characterization of Its Antibacterial Mechanism of Action. J. Med. Chem. 2016, 59 (7), 3569–3574. https://doi.org/10.1021/acs.jmedchem.6b00219
7. Schneider, T.; Gries, K.; Josten, M.; Wiedemann, I.; Pelzer, S.; Labischinski, H.; Sahl, H.-G. The Lipopeptide Antibiotic Friulimicin B Inhibits Cell Wall Biosynthesis through Complex Formation with Bactoprenol Phosphate. Antimicrob. Agents Chemother. 2009, 53 (4), 1610–1618. https://doi.org/10.1128/AAC.01040-08
8. Reynolds, K. A.; Luhavaya, H.; Li, J.; Dahesh, S.; Nizet, V.; Yamanaka, K.; Moore, B. S. Isolation and Structure Elucidation of Lipopeptide Antibiotic Taromycin B from the Activated Taromycin Biosynthetic Gene Cluster. J. Antibiot. (Tokyo). 2018, 71 (2), 333–338. https://doi.org/10.1038/ja.2017.146
9. Rubinchik, E.; Schneider T.; Elliott M.; Scott W. R. P.; Pan J., Anklin C.; Yang H.; Dugourd D.; Müller A.; Gries K.; et al. Mechanism of Action and Limited Cross-Resistance of New Lipopeptide MX-2401. Antimicrob. Agents Chemother. 2011, 55 (6), 2743–2754. https://doi.org/10.1128/aac.00170-11
10. Singh, M.; Chang, J.; Coffman, L.; Kim, S. J. Solid-State NMR Characterization of Amphomycin Effects on Peptidoglycan and Wall Teichoic Acid Biosyntheses in Staphylococcus Aureus. Sci. Rep. 2016, 6 (1), 31757. https://doi.org/10.1038/srep31757
11. Fernández-Pastor, I.; Ortiz-López, F. J.; Oves-Costales, D.; Martín, J.; Sánchez, P.; Melguizo, Á.; Reyes, F.; Weber, T.; Genilloud, O. Dilarmycins A–C, Calcium-Dependent Lipopeptide Antibiotics with a Non-Canonical Ca2+-Binding Motif. Org. Lett. 2024, 26 (7), 1343–1347. https://doi.org/10.1021/acs.orglett.3c04195

12. Lai, H.-E.; Woolner, V. H.; Little, R. F.; Woolly, E. F.; Keyzers, R. A.; Owen, J. G. Calcium-Dependent Lipopeptide Antibiotics against Drug-Resistant Pathogens Discovered via Host-Dependent Heterologous Expression of a Cloned Biosynthetic Gene Cluster. Angew. Chemie Int. Ed. 2024, 63 (48), e202410286. https://doi.org/https://doi.org/10.1002/anie.202410286
13. Makitrynskyy, R.; Keller, L.; Kaur, A.; Tsypik, O.; Munz, L.; Bechthold, A.; Müller, R. Olikomycin A–A Novel Calcium-Dependent Lipopeptide with Antibiotic Activity Against Multidrug-Resistant Bacteria. Chem. – A Eur. J. 2025, 31 (8), e202403985. https://doi.org/https://doi.org/10.1002/chem.202403985.
14. Bekiesch, P.; Zehl, M.; Domingo-Contreras, E.; Martín, J.; Pérez-Victoria, I.; Reyes, F.; Kaplan, A.; Rückert, C.; Busche, T.; Kalinowski, J.; Zotchev, S. B. Viennamycins: Lipopeptides Produced by a Streptomyces Sp. J. Nat. Prod. 2020, 83 (8), 2381–2389. https://doi.org/10.1021/acs.jnatprod.0c00152
15. Hover, B. M.; Kim, S.-H.; Katz, M.; Charlop-Powers, Z.; Owen, J. G.; Ternei, M. A.; Maniko, J.; Estrela, A. B.; Molina, H.; Park, S.; Perlin, D. S.; Brady, S. F. Culture-Independent Discovery of the Malacidins as Calcium-Dependent Antibiotics with Activity against Multidrug-Resistant Gram-Positive Pathogens. Nat. Microbiol. 2018, 3 (4), 415–422. https://doi.org/10.1038/s41564-018-0110-1
16. Wu, C.; Shang, Z.; Lemetre, C.; Ternei, M. A.; Brady, S. F. Cadasides, Calcium-Dependent Acidic Lipopeptides from the Soil Metagenome That Are Active against Multidrug-Resistant Bacteria. J. Am. Chem. Soc. 2019, 141 (9), 3910–3919. https://doi.org/10.1021/jacs.8b12087
